# Unravelling the Complexity of Lignosulfonates—Fractionation and Physicochemical Profiling

**DOI:** 10.1021/acs.biomac.5c02229

**Published:** 2025-12-09

**Authors:** Veslemøy Margrethe Selvik, Finn Lillelund Aachmann, Carlos Salas-Bringas, Vebjørn Eikemo

**Affiliations:** † Borregaard ASA, 1701 Sarpsborg, Norway; ‡ Ugelstad Laboratory, Department of Chemical Engineering, NTNU Norwegian University of Science and Technology, 7491 Trondheim, Norway; § Norwegian Biopolymer Laboratory (NOBIPOL), Department of Biotechnology and Food Science, NTNU Norwegian University of Science and Technology, 7491 Trondheim, Norway

## Abstract

Understanding the structural architecture of lignosulfonates is essential for optimizing their performance and enabling targeted modifications for sustainable applications. This study investigates six sodium lignosulfonate fractions (2,200–78,000 g/mol; dispersity 1.7–12.2) obtained via two-step ultrafiltration. Functional group analysis (sulfonation degree, phenolic hydroxyl, and carboxylic acid) using elemental analysis and NMR revealed minimal variation across fractions. Hydrophobic interaction chromatography and intrinsic viscosity measurements showed that increasing molecular weight correlates with reduced charge-to-size ratios and enhanced hydrophobicity. Rheological data showed that high-molecular-weight fractions exhibit greater conformational changes and compaction under high ionic strength. 2D NMR of purified fractions uncovered new structural features, including guaiacylethanol and fully characterized mono- and disulfonated bonding patterns. These findings advance the structural mapping of lignosulfonates and demonstrate that molecular weight is the dominant factor influencing the physical properties. The study highlights the value of combining fractionation, rheology, and NMR techniques to deepen our understanding of lignosulfonate structure and guide future lignin-based materials applications.

## Graphical abstract

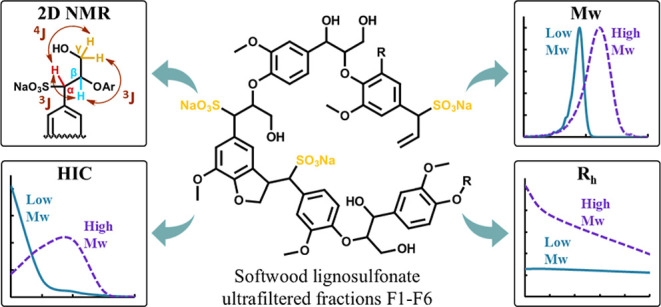

## Introduction

With increasing consideration of sustainability, lignosulfonates (LS) have emerged as sustainable and renewable^1^ alternatives to synthetic chemicals and products originating from the petroleum industry.^2^ Annually, an estimated 50 to 70 million tons^3^ technical lignin is produced, and lignosulfonate represents the second most abundant technical lignin, following kraft lignins.

Lignosulfonates are formed as coproducts with cellulose during the sulfite pulping process of wood. During the pulping process, natural lignin undergoes a series of chemical transformations, yielding randomly branched charged macromolecules with broad molecular weight distribution and diverse sulfonation patterns (Figure 1).^2^ The hydrophobic aromatic backbone combined with the hydrophilic sulfonate groups gives lignosulfonates their amphiphilic nature.^4^

**Figure 1:**
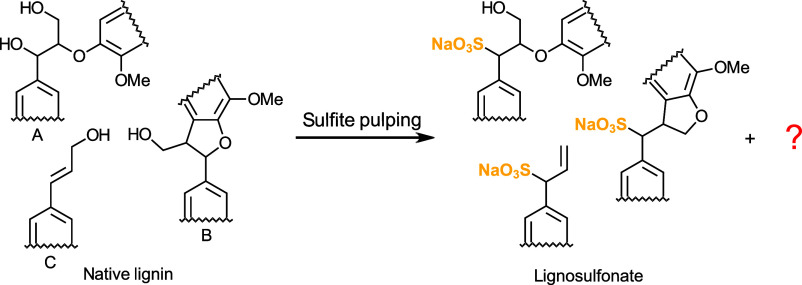
Sulfonation of typical lignin linkages. Suggested sulfonation reactions of lignin structures during the acidic sulfite pulping process: α-sulfonation of β-O-4 (A), rearrangement and α-sulfonation of β-5 (B), and rearrangement and α-sulfonation of coniferyl alcohol (C).^7–13^

The charged sulfonate groups provide water-solubility, which gives rise to a diverse set of functional properties and enables a wide variety of industrial applications.^5^ Lignosulfonates are commonly used as dispersants, binders, crystal growth modifiers, emulsion stabilizers, and complexing agents in industries such as construction, animal feed, lead acid batteries, and agriculture.^6^

Lignosulfonates exhibit substantial polydispersity in both functionality and molecular weight,^14^ which makes it challenging to directly correlate their chemical structure with application performance. One approach to addressing the challenge of polydispersity is to prepare samples with reduced variability; though some work has been done on fractionation on chemical characteristics,^14^ fractionation on molecular weight remains the most prevalent in the literature.^15–22^ Various technologies have been employed for this purpose^23^ including organic solvent extraction,^24^ gel column chromatography,^17^ and membrane separation.^19^ There is a broad agreement among authors using different starting materials and fractionation methods that the degree of sulfonation decreases with increasing molecular weight.^17,19–22,24^ Several studies, using ultrafiltration as the fractionation method, have shown that the carboxylic acid and methoxyl group content decreases with increasing molecular weight.^17–21^ However, the relationship between the phenolic hydroxyl content and molecular weight for fractionated samples is more disputed. Some authors report an increasing trend,^19,22^ while others observe a decreasing trend.^20,21^ In general, the variations between fractions are small, but some authors hypothesize that in larger molecular weight samples, a decrease in phenolic hydroxyl groups may result from cross-linking. Studies using hydrophobic interaction chromatography (HIC) have shown that the more hydrophobic fractions corresponded to a higher average molecular weight. Additionally, it was found that the charge-to-size ratio substantially influences the elution.^14^ This ratio is directly linked to the content of the hydrophilic functional group, particularly the degree of sulfonation. A lower degree of sulfonation leads to more exposed aromatic rings, resulting in increased hydrophobic characteristics.^2,17,20^

Since physicochemical properties are closely linked to chemical structure, it is essential to understand the functional groups and bonding patterns that constitute the lignosulfonate molecule. Structural elucidation using nuclear magnetic resonance (NMR) spectroscopy in lignosulfonates is mostly based on work done by Glennie in 1966 ^13^ and by Gellerstedt and co-workers from 1968 to 1977.^11,12,25–29^ Their studies employed model compound sulfonations representing typical bonding motifs, including coniferyl alcohol, β-aryl ether (β-O-4), phenylcoumaran (β-5), and pinoresinol (β–β) structures. These investigations explored key reactions such as α-sulfonation, aryl ether cleavage, and condensation, with α-sulfonation receiving particular attention in more recent literature.^7–10^ Notably, β-O-4 model compound sulfonation was extensively studied by Miles-Barrett and colleagues in 2017, wherein they elucidated diastereomers of the commonly occurring α-sulfonated analogue in technical lignosulfonates.^8^ Similarly, Musl and co-workers attributed characteristic NMR signals to the α-sulfonated β-O-4 motif, reinforcing its relevance in lignosulfonate structure analysis.^9,10^

The shape and conformation of lignosulfonates have been topics of interest for researchers in the past five decades. Myrvold (2008) shows, using experimental data from several sources, that lignosulfonate can be described as randomly branched polyelectrolytes.^30^ This was in contrast to the microgel model proposed by Rezanowich and Goring in 1960.^31^ While both models predict similar behavior, the branched polyelectrolyte model also accounts for molecular weight and solvent condition. Low-molecular-weight lignosulfonates are expected to adopt a more spherical shape, whereas higher-molecular-weight lignosulfonates tend to exhibit a more elongated shape. The polyelectrolyte model also accounts for reduced molecular expansion upon the addition of salt. With increased ionic strength, the Debye length is reduced, and screening of ionic functional groups results in a more compact structure.^2^ Mafé et al. proposed the existence of two hydration layers around the lignosulfonate,^32^ showing that the secondary layer is more easily lost at elevated ionic strength. Fredheim et al. found, using lignosulfonates derived from both hardwood and softwood sources, a Mark–Houwink–Sakurada exponent of 0.36 corresponding to a shape between Einstein spheres and random coils (θ-solvent).^33^ Vainio et al. describe lignosulfonate as an oblate spheroid with a 3.5 axial ratio.^34,35^ Sizes reported in the literature range from 4 to 7 nm in radius, as determined by techniques such as laser correlation spectroscopy,^36^ small-angle X-ray scattering,^34^ and dynamic light scattering.^37^

The aim of this study is to elucidate the relationship among chemical structure, molecular weight, and size in lignosulfonates. To achieve this, ultrafiltration was employed to obtain fractions with narrower molecular weight distributions, which were then characterized by the functional group content. NMR spectroscopy revealed previously unreported structural features and bonding patterns, contributing to a deeper understanding of the lignosulfonate architecture. HIC was used to assess hydrophobic behavior, while intrinsic viscosity measurements provided insight into molecular size as a function of both molecular weight and ionic strength.

## Experimental Section

### Materials

Sodium lignosulfonate derived from Norway spruce, provided by Borregaard AS (Sarpsborg, Norway), was used in all experimental procedures. DMSO-*d*_6_ (99.9 atom % D), D_2_O (99.9 atom % D), acetic acid-*d*_4_ (99.9 atom % D), CDCl_3_ (99.8 atom % D), sodium metabisulfite, 1,4-dioxane, *N*,*N*-dimethylformamide, pyridine, 2-chloro-4,4,5,5-tetramethyl-1,3,2-dioxaphospholane, coniferyl alcohol, and 3-(4-hydroxy-3-methoxyphenyl)-1-ethanol were purchased from Sigma-Aldrich and 3-(4-hydroxy-3-methoxyphenyl)-1-propanol and guaiacylglycerol-β-guaiacyl ether from VWR. All chemicals and solvents were ACS grade and used without further purification.

### Methods

#### Nuclear Magnetic Resonance Spectroscopy

For the NMR analysis, around 50 mg of dry lignosulfonate powder was dissolved in 500 μL of DMSO-*d*_6_ and 50 μL of D_2_O and transferred to 5 mm NMR tubes (Bruker, LabScape Stream). For lignosulfonate, all homo- and heteronuclear experiments were recorded at a temperature of 298.1 K on a Bruker Avance III HD 800 MHz spectrometer (Bruker BioSpin AG, Fällanden, Switzerland) equipped with a 5 mm cryogenic CP-TCI *z*-gradient probe. ^31^P NMR and model compound experiments were recorded at a temperature of 300 K on a Bruker 500 MHz Avance III instrument equipped with a 5 mm BBFO or SEI probe, respectively. Chemical shifts were calibrated using the residual solvent signal from DMSO-*d*_6_ as calibration reference (^1^H 2.50 ppm, ^13^C 39.52 ppm).^38^ J values are reported as Hz and multiplicities as s = singlet, d = doublet, t = triplet, q = quartet, dd = doublet of doublets, and m = multiplet. Spectra were recorded in TopSpin 3.5 pl7 or 3.6.5 and processed in TopSpin 4.0.7 (Bruker BioSpin AG), and 2D spectral coloring and signal annotation were performed in GIMP version 2.10.38.

For the structural characterization of lignosulfonate, 2D sets were collected for selected samples including the following experiments: 1D proton (zg30), 2D ^1^H–^13^C heteronuclear single quantum coherence (HSQC) with multiplicity editing (hsqcedetgpsisp2.3) 0.128 s acquisition time, 1.5 s relaxation delay (D1), 32 scans, and 135 Hz coupling constant (CNST2) over FID 2048 × 512 increments, ^1^H–^13^C heteronuclear two bond correlation (H2BC) spectroscopy (h2bcetgpl3pr) 0.125 s acquisition time, 2.0 s relaxation delay (D1), and 32 scans over 2048 × 256 increments, ^1^H–^13^C HSQC–^1^H,^1^H TOCSY (total correlation spectroscopy) 0.128 s acquisition time, 2.0 s relaxation delay (D1), 60 ms TOCSY mixing time (D9), 16 scans, and 135 Hz coupling constant (CNST2) over 2048 × 256 increments, and ^1^H–^13^C heteronuclear multiple bond coherence (HMBC) with suppression of one-bond correlations (hmbcetgpl3nd) 0.128 s acquisition time, 1.5 s relaxation delay, and 32 scans over 2048 × 512 increments. 2D HSQC integration was executed with the command int2d with a relative minimum threshold (MI) level of 0.0025.

Quantitative hydroxyl group estimation by ^31^P NMR was performed by a modified procedure reported by Meng et al.^39^ To this end, 200 mg of dry matter lignosulfonate was diluted in 3 mL of ultrapure water (UPW) and passed through a glass pipette containing Amberlite IR120 hydrogen form ion-exchange resin. The glass column was washed twice with 1 mL of water, and the eluted solution was freeze-dried overnight. 30 mg of the dried sample was suspended in 100 μL of 1:1 (v:v) DMF:pyridine, stirred for 30 min, followed by addition of 100 μL of internal standard solution (40 mg/mL cholesterol in pyridine). In a separate vial, 100 μL of 2-chloro-4,4,5,5-tetramethyl-1,3,2-dioxaphospholane (Cl-TMDP) was dissolved in 400 μL CDCl_3_ and added dropwise to the lignosulfonate solution. ^31^P NMR spectra were immediately acquired over 128 scans with a 15 s relaxation delay. The signal for hydrolyzed Cl-TMDP at 132.2 ppm was used as a calibration reference. Chemical shift ranges were integrated and attributed to the following functionalities: aliphatic hydroxyls (150–145.0 ppm), *o*-disubstituted phenols (144.3–140.2 ppm), *o*-monosubstituted phenols (140.2–138.4 ppm), *p*-hydroxyphenyl (138.4–136.9 ppm), and carboxylic acids (135.6–133.7 ppm).

Quantitative methoxyl group estimation was performed by 2D ^1^H–^13^C HSQC (hsqcetgp), 0.114 s acquisition time, 3.0 s relaxation delay (D1), 24 scans, and 145 Hz coupling constant (CNST2) over FID 1024 × 200 increments with nonuniform sampling (NUS) at 25% amount. For the analysis, 25 mg of dried lignosulfonate was dissolved in a 9:1 (v:v) mixture of DMSO-*d*_6_ and acetic acid-*d*_4_ (1 g). After 30 min stirring at rt, 650 μL was transferred to an NMR tube and HSQC NMR spectra were acquired at 353.0 K. A sodium lignosulfonate sample with a methoxyl group content of 3.54 mmol/g, estimated from the method reported by Vieböck and Schwappach,^40^ was used to prepare an external calibration curve following identical sample preparation. F1 projection was used for integration of the methoxyl signal at 53–58 ppm.

#### Size Exclusion Chromatography

Molecular weight (*M*_w_) was determined by size exclusion chromatography (SEC) as described by Fredheim.^24,33^ In this work, the MALLS detector was exchanged by a UV detector according to the modification to the method introduced by Ekeberg.^41^ Calibration was done with purified internal lignosulfonate standards prepared and analyzed by Fredheim through ethanol–water fractionation and SEC-MALLS for absolute *M*_w_.^24^

#### Elemental Analysis

Total sulfur and carbon were estimated with a Thermo FlashSmart CHNS/O element analyzer by applying a modified Dumas method. Inorganic sulfur was quantified using an ICS-5000+ ion chromatograph equipped with a Dionex Ionpac AG11-HC precolumn and a Dionex Ionpac AS11-HC column following the procedure reported by Myrvold.^42^ The difference between total and inorganic sulfur represents organic sulfur. The degree of sulfonation was calculated as the organic sulfur content divided by the methoxy content.

#### Organic Acids

Glycolic, lactic, formic, and acetic acid quantification was carried out on an Agilent 1100 HPLC-RI system equipped with a Bio-Rad Aminex HPX-87H cation exchange column and a Micro Guard Cation H^+^ Refill Cartridge guard column using isocratic elution with 17.5 mM sulfuric acid. A sodium lignosulfonate sample with known amounts of glycolic, lactic, formic, and acetic acid was used as a control to verify the accuracy of the method within 5% deviation.

#### Phenolic OH by UV/Vis

Ionization difference spectra of aqueous acidic and alkaline lignosulfonate solutions were recorded on a Thermo Evolution 220 UV–visible spectrophotometer. Phenolic OH content was estimated by multiplying net absorbance at 250 nm by the factor 0.192 according to eqs 1a and 1b, as described by Wexler.^43^

Equations 1a and 1b. Calculation of the phenolic hydroxyl groups.

1a $$A=A_{250nm}-\left(\frac{\left(A_{280nm}-A_{230nm}\right)\left(\lambda_{280nm}-\lambda_{250nm}\right)}{\lambda_{280nm}-\lambda_{230nm}}\right)$$

1b $$\%\;Phenolic\;OH=0.192\cdot \frac{A\cdot 100}{m\cdot \%\;DM}$$

#### Hydrophobic Interaction Chromatography

Hydrophobic interaction chromatography (HIC) was carried out according to Ekeberg et al.^41^ Stepwise elution was promoted by an ethanol/citric acid gradient in the mobile phase. Five elution peaks were recorded, and the integrated area was normalized to reflect the relative percentage of each peak.

### Procedures

#### Ultrafiltration

Sodium lignosulfonate with an average *M*_w_ of 43,500 g/mol and dispersity of 13.2 was chosen as the starting material (SM) for ultrafiltration. Six fractions were produced through a series of ultrafiltrations using a Millipore ProScale system. The membranes had molecular weight cut-offs (MWCO) ranging from 1 to 300 kDa and a filtration area of 0.1 m^2^, and the transmembrane pressure was kept between 3 and 4 MPa. The nomenclature of the lignosulfonate fractions is based on the molecular weight, where increasing sample index (F1–F6) implies increasing molecular weight.

For fractions F2–F5, two filtration steps were performed to ensure the exclusion of both low- and high-molecular-weight components. First, the start material was filtered through a low MWCO membrane. The retentate was then collected, diluted, and used as a feed for high MWCO filtration where the permeate was collected. Diawater was added to the second filtration. Fractions F1 and F6 were produced by a single filtration step. The scheme in Figure 2 illustrates the order of filtrations and the molecular weight cutoff of the membranes used in the preparation of each fraction.

**Figure 2:**
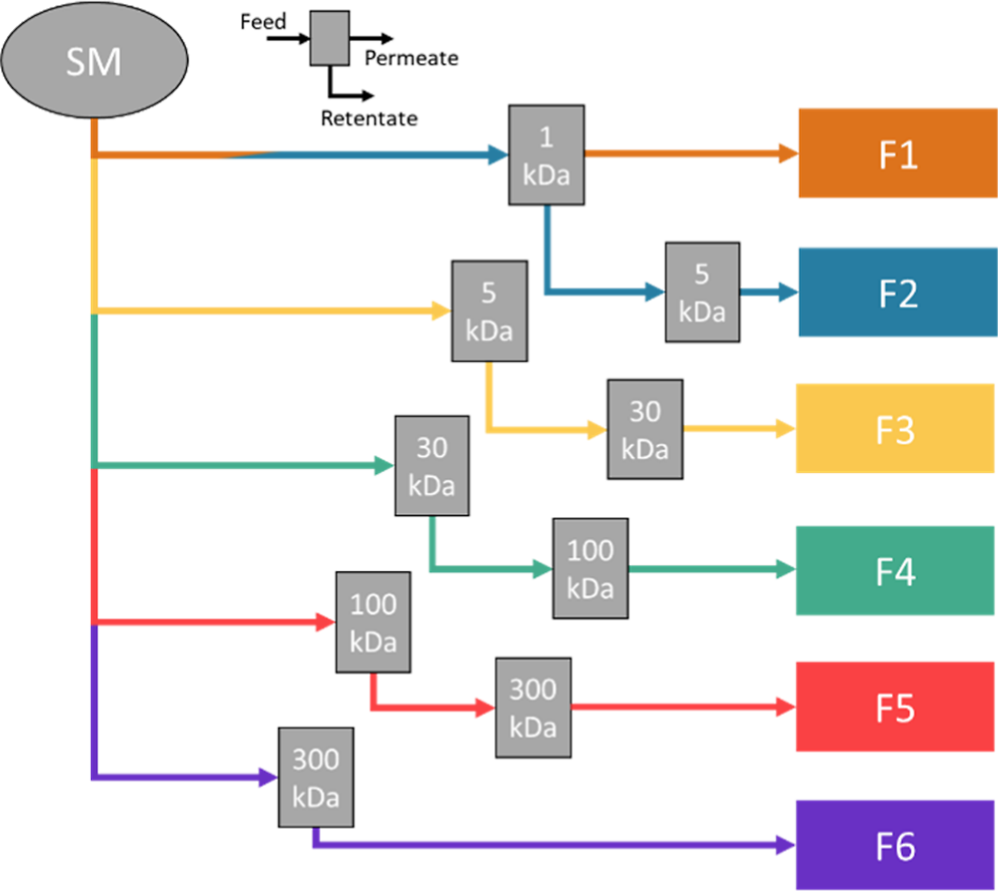
Ultrafiltration scheme. Fractionation on molecular weight was carried out through a two-step ultrafiltration method. First, the unfractionated lignosulfonate was filtered through a low MWCO membrane; the retentate was then filtered through a high MWCO membrane where the permeate was collected. The molecular weight cutoff of the membranes used is indicated in the figure.

#### Hydrodynamic Radius

Samples with lignosulfonate concentration ranging from 0.05 to 0.1 g/mL were prepared in either ultrapure water or ionic solution (0.1 or 0.5 M NaCl) and sonicated for 10 min before use. Dynamic viscosity was measured by rotational rheometry (Anton Paar MCR 102e) equipped with a double gap geometry (DG26.7) for increased surface area.^44^ The sample was presheared at 200/s for 1 min before the shear rate was reduced from 250 to 100/s exponentially. All measurements were conducted at a constant temperature of 20 °C, with three replicates performed for each concentration. Dilute lignosulfonate solutions exhibit Newtonian behavior, and so, the dynamic viscosity was taken as an average over all measurement points. Intrinsic viscosity was calculated according to eqs 2a–2d.

Equations 2a–2d. Intrinsic viscosity

2a $$\eta_{rel}=\frac{\eta_{solution}}{\eta_{solvent}}$$

2b $$\eta_{sp}=\eta_{rel}-1$$

2c $$\eta_{red}=\frac{\eta_{sp}}{C}$$

2d $$\left[\eta \right]=\lim_{c\to 0}\left(\eta_{red}\right)$$

where η_rel_ is the relative viscosity defined by the viscosity of the lignosulfonate solution (η_solution_) and the viscosity of the pure solvent (η_solvent_). η_sp_ is the specific viscosity, η_red_ is the reduced viscosity, and *C* is the concentration.

Extrapolation of reduced viscosity to zero-concentration yields intrinsic viscosity ([η]). Given the molar mass (*M*), the hydrodynamic radius (*R*_h_) can be estimated by using the Einstein–Simha equation (eq 3), where *N*_A_ is Avogadro’s number.

Equation 3. Einstein–Simha

3 $$R_{h}={\left(\frac{3\left[\eta \right]M}{10\pi N_{A}}\right)}^{1/3}$$

Although this equation assumes spherical particles, the resulting value represents an effective hydrodynamic radius, which facilitates comparative analysis across different molecular weight fractions.^45^

#### Model Compound Sulfonations

##### 3-Hydroxy-1-(4-hydroxy-3-methoxyphenyl)-2-(2-methoxyphenoxy)-propane-1-sulfonic acid Sodium Salt (**2—*anti*, 3—*syn***)

A 15 mL Ace glass pressure tube was charged with guaiacylglycerol-β-guaiacyl ether (**1**) (540 mg, 1.69 mmol), 1,4-dioxane (1 mL), 40% aq solution of NaHSO_3_ (1.25 mL, 14.9 mmol), H_2_O (2.8 mL), and 95–97% H_2_SO_4_ (2 drops). The tube was sealed and heated to 135 °C for 4 h. Following cooling to rt, the reaction mixture was concentrated in vacuo, and the residue was suspended in MeOH (10 mL) and filtered to remove salts. The filtrate was concentrated in vacuo, and the salt removal step was repeated three more times. The dried residue (770 mg) was analyzed by NMR spectroscopy without further purification, which uncovered **2** and **3** as diastereomers in a 82:18 ratio, as well as unreacted **1**.

Major diastereomer **2**: ^1^H NMR (500.13 MHz, DMSO-*d*_6_): δ 7.10 (d, *J* = 1.9 Hz, 1H, H2), 7.01 (dd, *J* = 6.0, 3.8 Hz, 1H), 6.85–6.81 (m, 1H), 6.85–6.76 (m, 2H), 6.74 (dd, *J* = 8.1, 1.9 Hz, 1H, H6), 6.57 (d, *J* = 8.1 Hz, 1H, H5), 4.85 (dt, *J* = 5.6, 5.0 Hz, 1H, Hβ), 3.91 (d, *J* = 6.4 Hz, 1H, Hα), 3.64 (s, 3H, OMe), 3.61–3.59 (m, 1H, Hγ), 3.57 (s, 3H, OMe), 3.49 (dd, *J* = 11.8, 5.5 Hz, 1H, Hγ). ^13^C NMR (125.76 MHz, DMSO-*d*_6_): δ 149.9 (C1′), 145.1 (C4), 149.9 (C2′), 146.3 (C3), 127.9 (C1), 123.6 (C6), 121.0, 120.7, 115.9, 114.9 (C2), 113.9 (C5), 113.0, 80.3 (Cβ), 66.2 (Cα), 61.4 (Cγ). Minor diastereomer **3**: ^1^H NMR (500.13 MHz, DMSO-*d*_6_): δ 7.26 (m, 1H), 6.92–6.88 (m, 1H, overlapping), 6.90–6.87 (m, 1H, overlapping), 6.80–6.75 (m, 1H), 4.97 (dt, *J* = 8.2, 2.6 Hz, 1H, Hβ), 4.04 (dd, *J* = 11.6, 2.9 Hz, 1H, Hγ), 3.94 (d, *J* = 2.4 Hz, Hα), 3.72 (s, 3H, OMe), 3.67 (s, 3H, OMe), 3.06 (dd, *J* = 11.5, 8.4 Hz, 1H, Hγ). ^13^C NMR (125.76 MHz, DMSO-*d*_6_): δ 149.8 (C2′), 146.5 (C3), 122.7, 120.9, 114.9, 114.5, 79.7 (Cβ), 61.4 (Cγ), 64.7 (Cα), 55.6 (OMe, overlapping), 55.6 (OMe, overlapping). Remaining aromatic signals are not resolved due to significant overlap with **1** and **2**. Spectroscopic data is in accordance with previously reported literature.^8,46^

##### (*E*)-3-(4-Hydroxy-3-methoxyphenyl)-2-propene-1-sulfonic acid Sodium Salt (**5**) and 3-(4-Hydroxy-3-methoxyphenyl)-2-propene-1-sulfonic acid Sodium Salt (**6**)

A 15 mL Ace glass pressure tube was charged with coniferyl alcohol (**4**) (138 mg, 0.77 mmol), 1,4-dioxane (1.5 mL), 40% aq solution of NaHSO_3_ (1.9 mL, *d* = 1.34 g/mL, 9.80 mmol), H_2_O (4.1 mL), and 95–97% H_2_SO_4_ (3 drops). The tube was sealed and heated to 135 °C for 4 h. Following cooling to rt, the reaction mixture was concentrated in vacuo and the residue was suspended in MeOH (10 mL) and filtered to remove salts. The filtrate was concentrated in vacuo, and the salt removal step was repeated two more times with methanol (10 mL) and once with 1:1 (v:v) methanol:isopropanol (10 mL). The dried residue was analyzed by NMR spectroscopy without further purification, which uncovered the presence of **5** and **6** in a 36:64 ratio as major products along with traces of 1-(4-hydroxy-3-methoxyphenyl)-1,3-propanedisulfonic acid disodium salt (**7**) and 1-(4-hydroxy-3-methoxyphenyl)-1,2-propanedisulfonic acid disodium salt (**8**) and dihydroconiferyl alcohol (**9**).

**5**: ^1^H NMR (500.13 MHz, DMSO-*d*_6_): δ 6.95 (d, *J* = 1.7 Hz, 1H, H2), 6.77 (dd, *J* = 8.2, 1.7 Hz, 1H, H6), 6.72–6.70 (m, 1H, H5, overlapping with H6 from **6**), 6.32 (d, *J* = 16.0 Hz, 1H, Hα), 6.06 (dt, *J* = 16.0, 7.4 Hz, 1H, Hβ), 3.71 (s, 3H, OMe), 3.35 (d, *J* = 7.4 Hz, 2H, Hγ). ^13^C NMR (125.76 MHz, DMSO-*d*_6_): δ 146.8 (C3), 146.0 (C4), 133.1 (Cα), 128.9 (C1), 120.0 (Cβ), 119.4 (C6), 115.5 (C5), 109.5 (C2), 55.6 (OMe), 55.5 (Cγ). HRMS (ESI): calcd for C_10_H_11_O_5_S [M – H]^−^, 243.0333; found, 243.0323.

**6**: ^1^H NMR (500.13 MHz, DMSO-*d*_6_): δ 6.94 (d, *J* = 1.8 Hz, 1H, H2), 6.72–6.70 (m, 1H, H6, overlapping with H5 from **5**), 6.64 (d, *J* = 8.1 Hz, 1H, H5), 6.17 (ddd, *J* = 17.2, 10.1, 8.3 Hz, 1H, Hβ), 5.05 (dd, *J* = 10.2, 1.0 Hz, 1H, Hγ), 4.99 (dd, *J* = 17.2, 1.0 Hz, 1H, Hγ), 4.18 (d, *J* = 8.3 Hz, 1H, Hα), 3.72 (s, 3H, OMe). ^13^C NMR (125.76 MHz, DMSO-*d*_6_): δ 147.7 (C3), 145.1 (C4), 137.3 (Cβ), 129.8 (C1), 121.7 (C6), 116.6 (Cγ), 114.7 (C5), 113.4 (C2), 69.5 (Cα), 55.6 (OMe). HRMS (ESI): calcd for C_10_H_11_O_5_S [M – H]^−^, 243.0333; found, 243.0323.

**7**: ^1^H NMR (500.13 MHz, DMSO-*d*_6_): δ 3.52 (1H, Hα), 2.47 (1H, Hβ), 2.37 (1H, Hγ), 2.20 (1H, Hγ), 2.06 (1H, Hβ). ^13^C NMR (125.76 MHz, DMSO-*d*_6_): δ 64.4 (Cα), 49.7 (Cγ), 27.7 (Cβ). Aromatic signals are not resolved due to their low abundance and overlap. HRMS (ESI): calcd for C_10_H_13_O_8_S [M – H]^−^, 325.0057; found, 325.0050.

**8**: ^1^H NMR (500.13 MHz, DMSO-*d*_6_): δ 4.14 (d, *J* = 5.0 Hz, 1H, Hα), 2.98–2.92 (m, 1H, Hβ), 1.56 (d, *J* = 7.2 Hz, 3H). ^13^C NMR (125.76 MHz, DMSO-*d*_6_): δ 65.5 (Cα), 59.7 (Cβ), 14.0 (Cγ). Aromatic signals are not resolved due to low abundance and overlap. HRMS (ESI): calcd for C_10_H_13_O_8_S [M – H]^−^, 325.0057; found, 325.0050.

**9**: ^1^H NMR (500.13 MHz, DMSO-*d*_6_): δ 6.72 (1H, not resolved), 6.65 (1H, not resolved), 6.56 (1H, not resolved), 3.71 (3H, OMe, not resolved), 3.38 (2H, not resolved), 2.47 (2H, not resolved), 1.68–1.62 (m, 2H, Hβ). ^13^C NMR (125.76 MHz, DMSO-*d*_6_): δ 147.5 (C3), 144.2 (C4), 133.3 (C1), 120.4 (C6), 115.2 (C5), 112.6 (C2), 60.2 (Cγ), 55.6 (OMe), 34.5 (Cβ), and 31.3 (Cα). Signal assignments based on comparison with reference material in HSQC.

## Results and Discussion

### Fractionation into Fractions with Lower Dispersity

The molecular weight distributions of the starting material and fractionated samples are shown in Figure 3. The black line represents the starting material, which shows a broad molecular weight distribution. Fraction F1 displays a “mixed distribution” characterized by a higher proportion of low-molecular-weight components. This fraction was produced through a single filtration where the permeate was collected and therefore a higher content of impurities is expected. Fractions F2, F3, F4, and F5 underwent two filtration steps, resulting in narrower distributions with distinct molecular weights. In contrast, F6 was filtered once through a 300 kDa membrane and shows a broader distribution, indicating a wider range of molecular weights.

**Figure 3:**
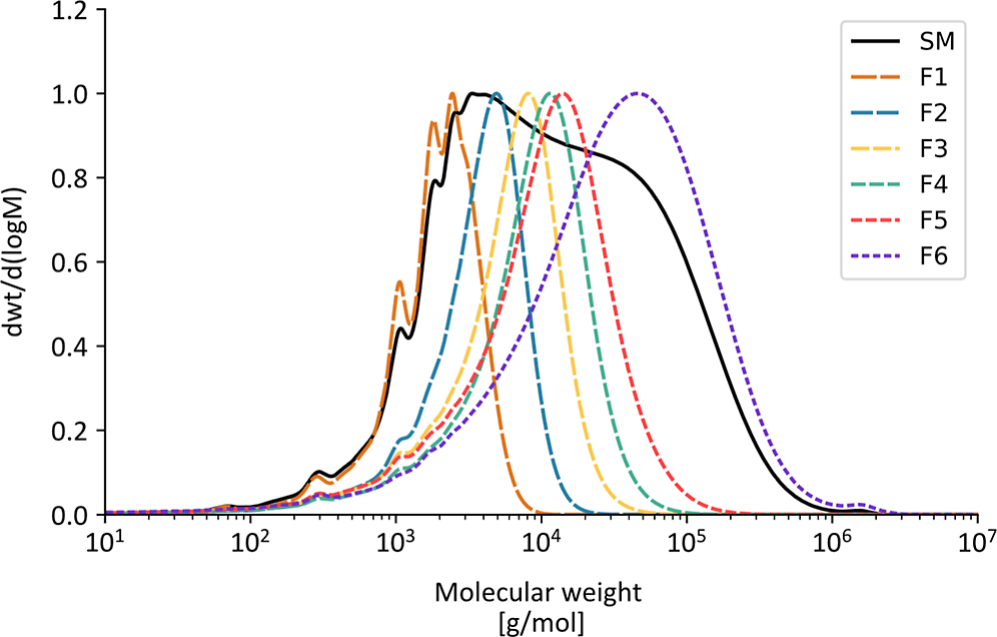
Molecular weight distributions of fractionated lignosulfonate. Molecular weight distributions of sodium lignosulfonate (SM, black) and lignosulfonate fractionated on molecular weight by ultrafiltration (F1–F6). F1 shows the presence of high-concentration low-molecular-weight impurities, while the remaining fractions (F2–F6) show distinct distributions, though with some overlap in the low molecular range.

The weight (*M*_w_) and number-average (*M*_n_) molecular weight as well as dispersity (*D̵*) (defined as (*M*_w_/*M*_n_)) are summarized in Table 1.

**Table 1:** Molecular Weight and Dispersity^a^

	*M*_w_ [g/mol]	*M*_n_ [g/mol]	*D̵* [*M*_w_/*M*_n_]
SM	43,500	3,300	13.2
F1	2,200	1,300	1.7
F2	4,500	2,200	2.0
F3	7,600	2,700	2.8
F4	11,500	3,500	3.3
F5	16,200	3,600	4.5
F6	78,000	6,500	12.2

a Weight average (*M*_w_) and number average (*M*_n_) molecular weight as well as the ratio between them (dispersity, *D̵*) for the unfractionated lignosulfonate (SM) as well as six fractionated samples. Molecular weight distributions are shown in Figure 3.

Caution should be taken when comparing the absolute molecular weights reported in the literature. Many studies use sodium polystyrenesulfonate (PSS) as a standard to determine molecular weight, which can cause underestimation due to differences in shape and extension.^33^ In this study, lignosulfonate fractions with low dispersity prepared and characterized by Fredheim et al. were used as standards.^24^ These fractions were previously characterized by SEC-MALLS and should therefore give more accurate molecular weights compared to results obtained with nonlignosulfonate standards. Since the SEC-UV approach relies on relative calibration (standards) as opposed to a direct measurement like with SEC-MALLS, quantitative comparisons should be approached carefully. Therefore, it is more appropriate to focus on relative trends and general behavior. Similar fractionation has been reported by other authors with start material molecular weight ranging between 5,500 and 20,500 g/mol.^19–22^ It should be noted that the lower-molecular-weight samples are typically associated with hardwood lignosulfonate and that the use of PSS as a calibration standard often results in lower reported average molecular weight.^33^ In this study, the dispersity was reduced from 13.2 for the start material to 2.0–4.5 for the samples fractionated by a two-step ultrafiltration procedure, showing a narrowing of the molecular weight distribution.

### Chemical Characterization Displays More Consistent Fractions

The generated fraction composition in terms of the methoxyl, carboxylic, phenolic hydroxyl, and sulfonic functional groups is given in Table 2.

**Table 2:** Functional Group Quantification^a^

	degree of sulfonation [–]	methoxyl [mmol/g]	carboxylic acid ^31^P NMR [mmol/g]	phenolic hydroxyl ^31^P NMR [mmol/g]	phenolic hydroxyl UV/vis [mmol/g]
SM	0.55	3.1	0.28	1.6	1.1
F1	1.45	1.3	0.86	1.1	0.5
F2	0.54	3.9	0.23	1.6	1.2
F3	0.42	4.1	0.14	1.5	1.2
F4	0.43	4.2	0.11	1.4	1.3
F5	0.41	4.2	0.15^b^	1.1^b^	1.2
F6	0.45	3.6	0.05^b^	0.4^b^	1.2

a Chemical characterization of fractions shows that F1 diverges from the fractionated samples (F2–F6) due to high amounts of impurities. The remaining fractions show little variation in the degree of sulfonation and methoxyl, carboxylic acid, and phenolic hydroxyl content. ^31^P NMR values were not normalized by actual dissolved weight, and some underestimation due to low solubility of high-molecular-weight fractions F5 and F6 is observed.

b Underestimated values due to observation of insoluble matter in NMR sample.

The degree of sulfonation is calculated as organic sulfur per molar methoxy content. Only small variations in organic sulfur content were observed (1.6 to 2.1 mmol/g), so methoxy content variations are accountable for the large difference between the fractions and F1. An overestimation of the sulfonation degree (1.45 mmol/g) for F1 is likely due to an underestimation of methoxyl content possibly caused by the presence of low molecular weight, nonlignosulfonate components. This aligns well with previous studies that consistently report a decrease in the degree of sulfonation with increasing molecular weight.^17,19–22,24^ A modest decrease in the sulfonation degree (0.54 to 0.45) is also observed in this work.

Hydroxyl group estimation by ^31^P NMR revealed that the carboxylic acid levels are low in both the starting material and all ultrafiltered fractions, except F1. This is largely due to the presence of low-molecular-weight organic acids, such as glycolic, lactic, formic, and acetic acids, which were determined by HPLC-RI (Table S2). These organic acids account for 2.2 and 0.7 mmol/g COOH in F1 and SM, respectively, and in the ^31^P NMR spectra, these compounds appear as sharp singlets in contrast to the broad and ill-defined signals from lignosulfonate macromolecules (Figures S52 and S53). As a result, the carboxylic acid values reported in Table 2 are overestimated and do not reflect the true carboxylic content on the lignosulfonate structure. In F2–F4, these signals are less prominent (Figures S54–S56) and therefore the values from Table 2 are more representative of actual COOH content. Since the levels from ^31^P NMR are already low, this means that there are hardly any carboxylic acids present, which align well with recent findings by Wurzer et al.^47^ It should be noted that F5 and F6 did not dissolve properly in the reagent solution, likely resulting in underestimation. The values were not normalized by actual dissolved weight and, hence, rendered incomparable with the remaining fractions. When potentiometric titration is used instead of ^31^P NMR, other studies have reported higher carboxylic acid values and a decreasing trend with increasing molecular weight.^19–21^ This is likely a result of overestimation, especially in low-molecular-weight fractions, and the method’s inability to distinguish between carboxylate groups on lignosulfonate from small monomeric acids.

Phenolic hydroxyl content is consistent (1.2–1.3 mmol/g) across all fractions, except for F1, as determined by UV/vis spectroscopy. This contrasts with published literature, where both increasing and decreasing amounts of phenolic hydroxyl have been reported.^19–22^ When estimated by ^31^P NMR spectroscopy, a slight declining trend with increasing molecular weight was observed, although the significance of this trend is uncertain. Minimal variations in phenolic hydroxyl levels suggested that molecular weight is not primarily built through ether cross-linking as this should result in a decreasing trend. Instead, condensation reactions are more responsible for building molecular weight. Phenolic hydroxyl estimated by ^31^P NMR is slightly higher than those determined by the spectrophotometric method, likely due to differences in the sample preparation. In the NMR method, the sample is passed through Amberlite IR120 ion-exchange resin in order to remove salts prior to freeze-drying and derivatization. This results in a sample of higher purity and therefore higher apparent phenolic hydroxyl content. In contrast, the UV method does not utilize any sample purification procedure.

### Unreported Lignosulfonate Structure Identified through NMR

Standard analytical methods used for lignosulfonate characterization such as FT-IR, SEC, and NMR often rely on isolation protocols to obtain high purity materials, facilitating analysis and simplified data interpretation. However, this could potentially lead to a loss of important compositional information, particularly given limited knowledge of structure–activity relationships of lignosulfonates. One such method is the XAD-7 resin adsorption–desorption protocol described by Sumerskii and co-workers, which enables rapid and reproducible sample purification.^48^ Despite its efficiency, this approach leads to partial or complete removal of the sulfonated β-O-4 bonding pattern, as evidenced by the NMR spectra. This is a notable limitation as the β-O-4 structure is considered a major sulfonated structure that exists in lignosulfonates. These findings underscore the need for development and improvement of characterization techniques capable of analyzing crude lignosulfonates without or with limited purification. Two-dimensional nuclear magnetic resonance (2D NMR) spectroscopy has been identified as a promising, yet underutilized, tool for this purpose.

The α-sulfonated β-O-4 bonding pattern is among the key structural features present in lignosulfonates and has been frequently reported in recent literature.^7,9,10,49^ The β-CH signal is well-established, and Miles-Barrett and co-workers even detected the presence of two diastereomers within this specific cross peak at 4.9 (^1^H)/79.9 (^13^C) ppm. The purified low-molecular-weight fraction (F2) enabled the detection of diastereomeric signals for the γ-CH_2_, as illustrated in the HSQC spectrum with correlations from HSQC-TOCSY (Figure 4). Due to the stereocenters and hence variations in ^3^ *J* coupling constants, the γ-signal splits into two pairs for each pair of diastereomers. For the α-CH, there were no observable TOCSY correlations to what is believed to be the signal for the minor diastereomer.

**Figure 4:**
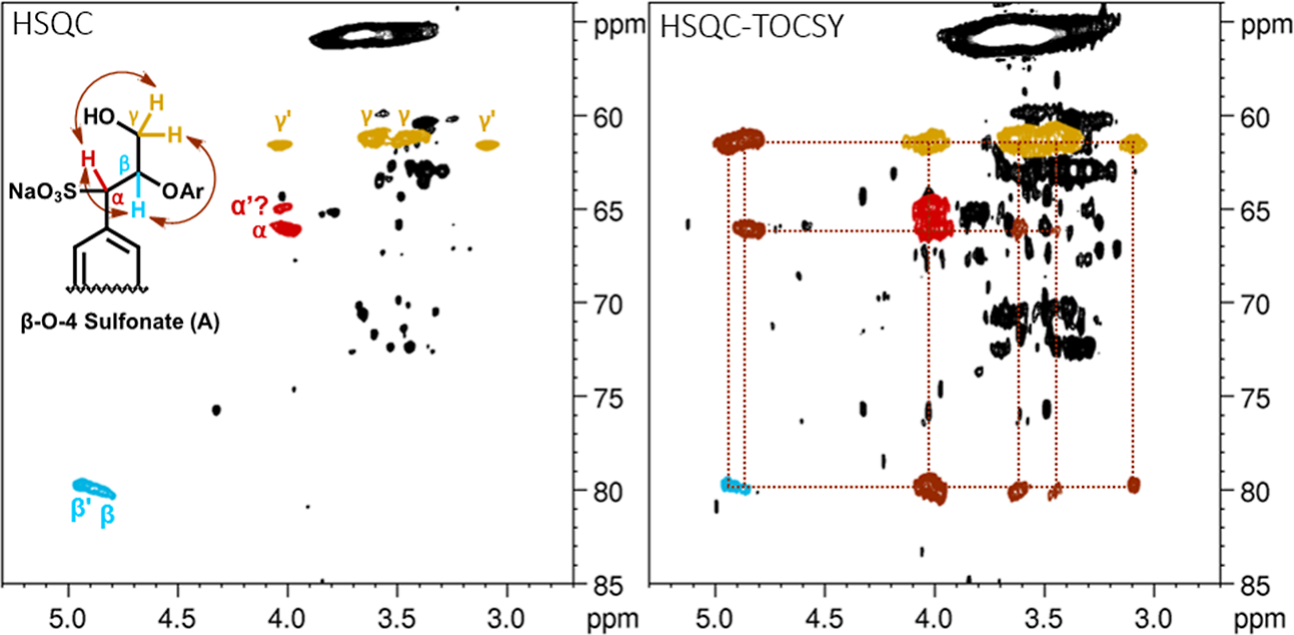
Characterization of the α-sulfonated β-O-4 moiety. Zoomed-in area of ^1^H–^13^C-HSQC and HSQC-TOCSY NMR spectra of fraction F2 recorded at 298.1 K, 800 MHz. The structure fragment of the α-sulfonated β-O-4 moiety is shown in the HSQC panel, where signals corresponding to α (red), β (blue), and γ (orange) positions are highlighted. Brown arrows, cross peaks, and dotted lines indicate TOCSY correlations within the spin system.

To confirm the identity of the diastereomeric α-CH signals, guaiacylglycerol-β-guaiacyl ether was sulfonated according to the procedure reported by Miles-Barrett.^49^ The reaction conditions simulate the acidic sulfite pulping process and result in the α-sulfonated β-O-4 bonding pattern (Scheme 1).

**Scheme 1:**
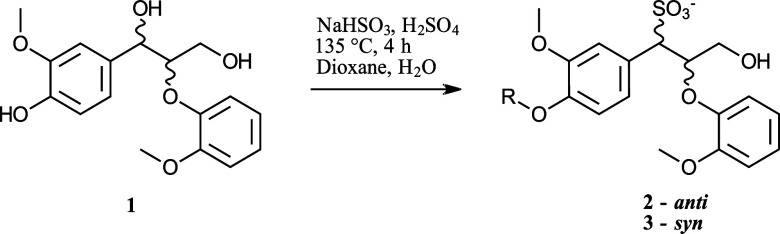
Sulfonation of β-O-4 Model Compound Guaiacylglycerol-β-guaiacyl ether (**1**) at Simulated Acidic Sulfite Pulping Conditions[Fn s1fn1]

a
*Anti* and *syn* diastereromers **2** and **3** were formed in a 82:18 ratio as determined by integrals in ^1^H NMR.

The expected α-sulfonated β-O-4 model was formed as a mixture of *anti* and *syn* diastereomers (**2** and **3**, 82:18 ratio from ^1^H NMR integrals), which has been reported and characterized previously by Miles-Barrett and colleagues.^49^ Comparison of the NMR spectra from the crude reaction mixture and fraction F2 showed a perfect overlap of the β-CH signals for both diastereomers. Additionally, α- and γ-signals corresponding to both diastereomeric pairs were detected (Figure 5). Model compound sulfonation and fractionation combined with NMR enabled mapping of the chemical shift values across the entire aromatic system in the α-sulfonated β-O-4 bonding pattern (Table S4 and Figure S50).

**Figure 5:**
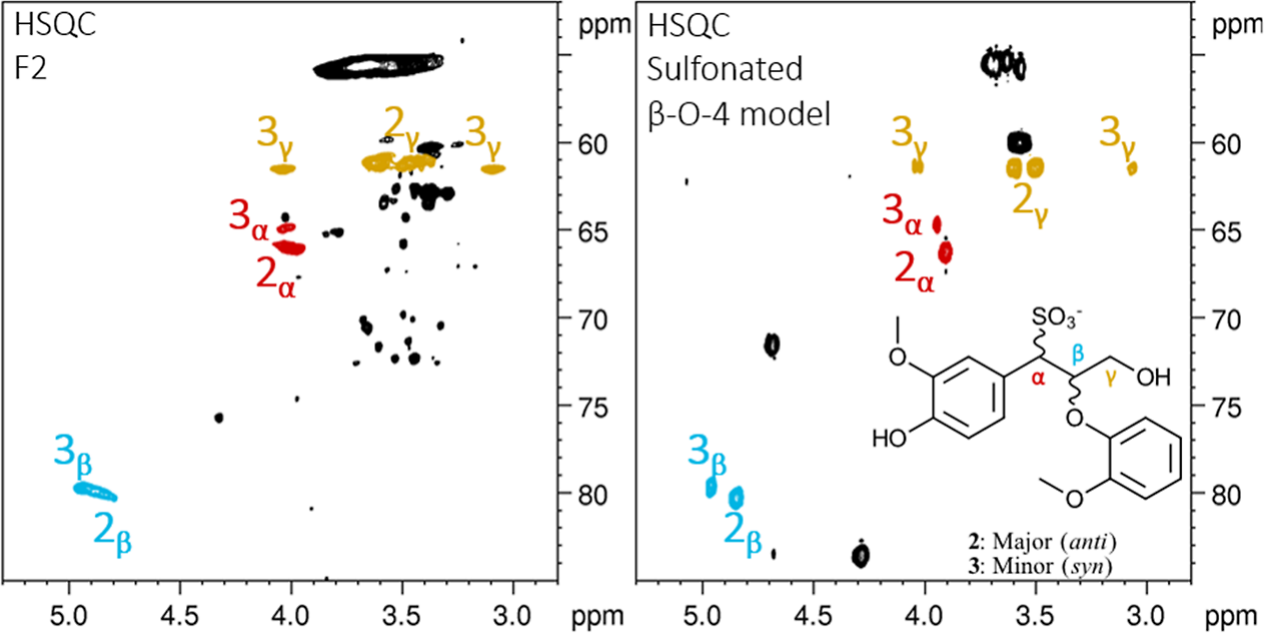
Side-by-side comparison of sulfonated guaiacylglycerol-β-guaiacyl ether to fraction F2 lignosulfonate. Zoomed-in area of interest of ^1^H–^13^C-HSQC-NMR spectra recorded at 298.1 K and 800 MHz. Side-by-side comparison of sodium lignosulfonate F2 (left) and the crude reaction mixture of sulfonated guaiacylglycerol-β-guaiacyl ether (right). Signals corresponding to the α (red), β (blue), and γ (orange) positions are assigned. Diastereomer guaiacylglycerol-β-guaiacyl ether in *anti* conformation is indicated by **2**, while diastereomer for *syn* conformation corresponds to **3**. Remaining signals in black are from unreacted **1**.

Other sulfonated structures previously mentioned by Glennie in 1966 ^13^ and Marques in 2009 ^7^ and since absent from recent literature are allyl-1-sulfonate and propane-1,3-disulfonate. The characteristic signals from the allyl-1-sulfonate group (δ_H_/δ_C_: 6.2/136.9, 5.1/117.2, and 4.2/69.6 ppm) as well as the γ-methylene group from propane-1,3-disulfonate (δ_H_/δ_C_: 2.3/49.7 ppm) were indeed observed in the HSQC spectrum of fraction F2 and corresponded well with the reported values (Figure 6).^7^ However, the β-signal in the latter structure (δ_C_: 33.0 ppm) did not match the literature, and no signal corresponding to the α-sulfo group was detected, likely due to low resolution or sensitivity.

**Figure 6:**
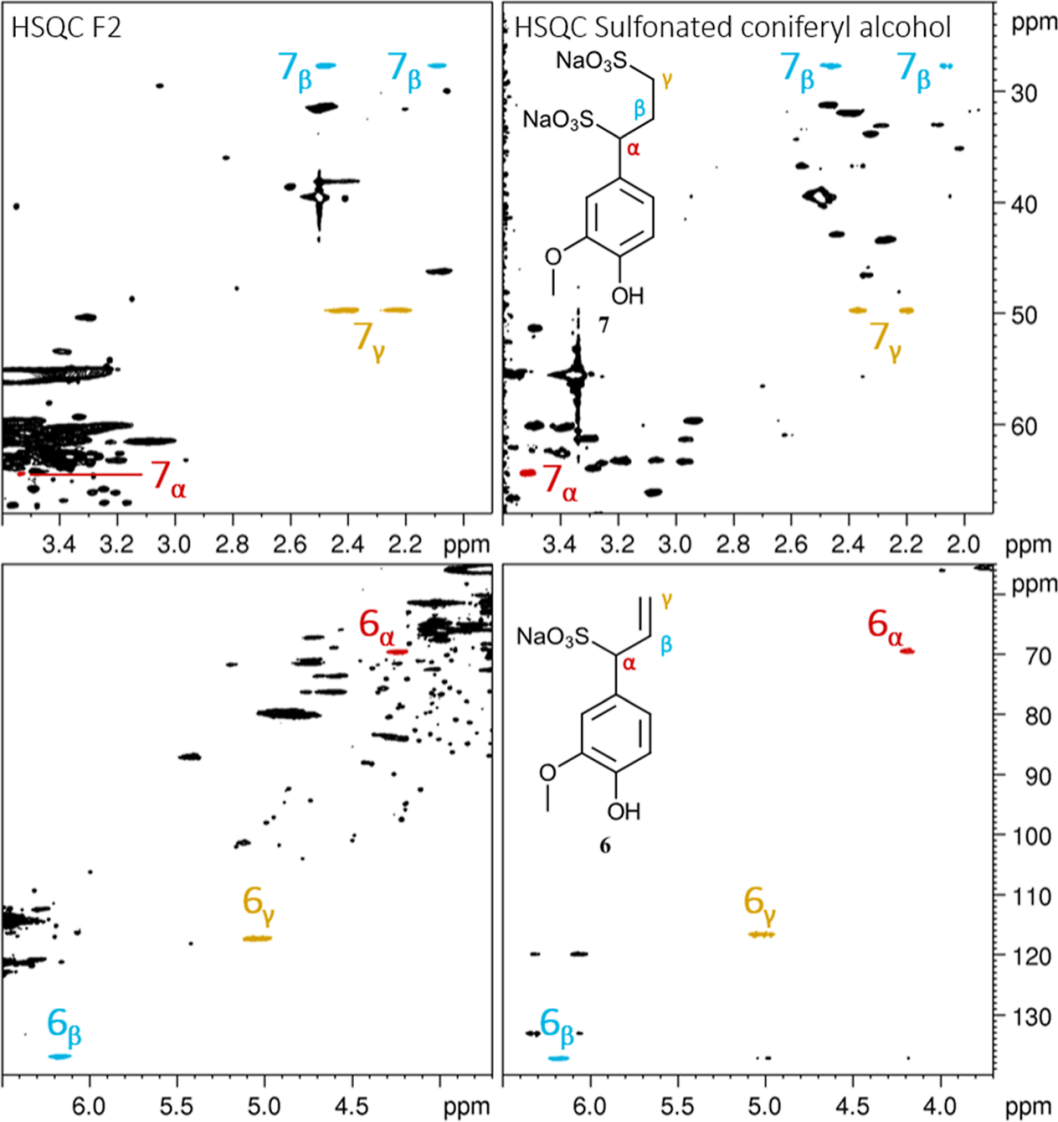
Side-by-side comparison of sulfonation of coniferyl alcohol to fraction F2 lignosulfonate. Zoomed-in area of interest of ^1^H–^13^C-HSQC-NMR spectra recorded at 298.1 K and 800 MHz. Side-by-side comparison of sodium lignosulfonate (left) and the crude reaction mixture from sulfonated coniferyl alcohol at mimicked acidic sulfite pulping conditions (right, Scheme 2). Signals corresponding to the α (red), β (blue), and γ (orange) positions are assigned.

To verify the identity of both of these structures, coniferyl alcohol (**4**) was a suitable substrate due to its expected S_N_1 reactivity and carbocation resonance stabilization. Following sulfonation in mimicked acidic sulfite pulping conditions (Scheme 2), four sulfonated products were detected: the expected monosulfonates **5** and **6** as well as disulfonates **7** and **8**. The monosulfonated products are likely formed through an S_N_1 mechanism with initial formation of a primary carbocation that rapidly rearranges to a more stable benzylic resonance structure. Subsequent nucleophilic attack by the bisulfite anion leads to sulfonates **5** and **6** in a 36:64 ratio, as determined by integrals in the ^1^H NMR spectrum of the crude reaction mixture, highlighting the superior stability of the benzylic carbocation. Secondary sulfonation likely proceeds via acid-catalyzed Markovnikov addition of bisulfite to the double bonds, yielding disulfonates **7** and **8**. An unexpected discovery was the presence of dihydroconiferyl alcohol (**9**) in the reaction mixture. To the best of our knowledge, alkene reduction using sodium bisulfite has not been previously reported. Intriguingly, of the four mono- and disulfonates detected, only compounds **6** and **7** were observed in the HSQC spectrum of the F2 fraction (Figure 7). This is the first time disulfonate **7** is fully characterized by NMR and confirmed as a constituent of commercial softwood lignosulfonates, as far as is currently known.

**Scheme 2:**
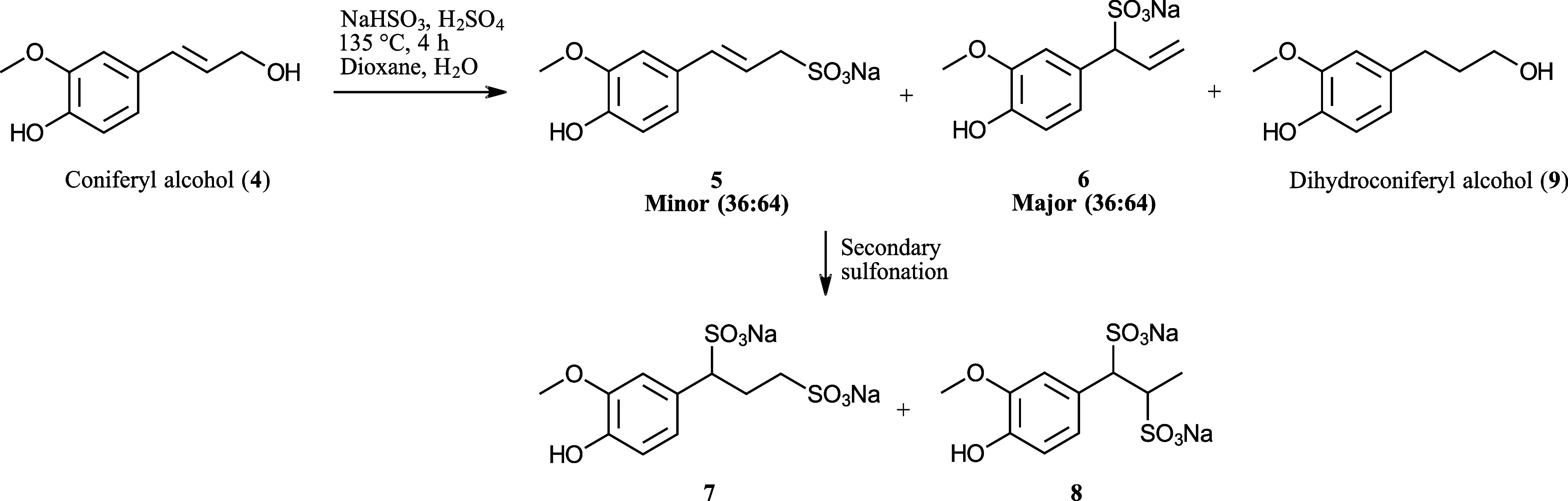
Sulfonation of Coniferyl Alcohol at Simulated Acidic Sulfite Pulping Conditions^a^

a Monosulfonates **5** and **6** were formed as major products in a 36:64 ratio as determined by integrals in ^1^H NMR. Disulfonates **7** and **8** as well as dihydroconiferyl alcohol (**9**) were present in trace amounts.

**Figure 7:**
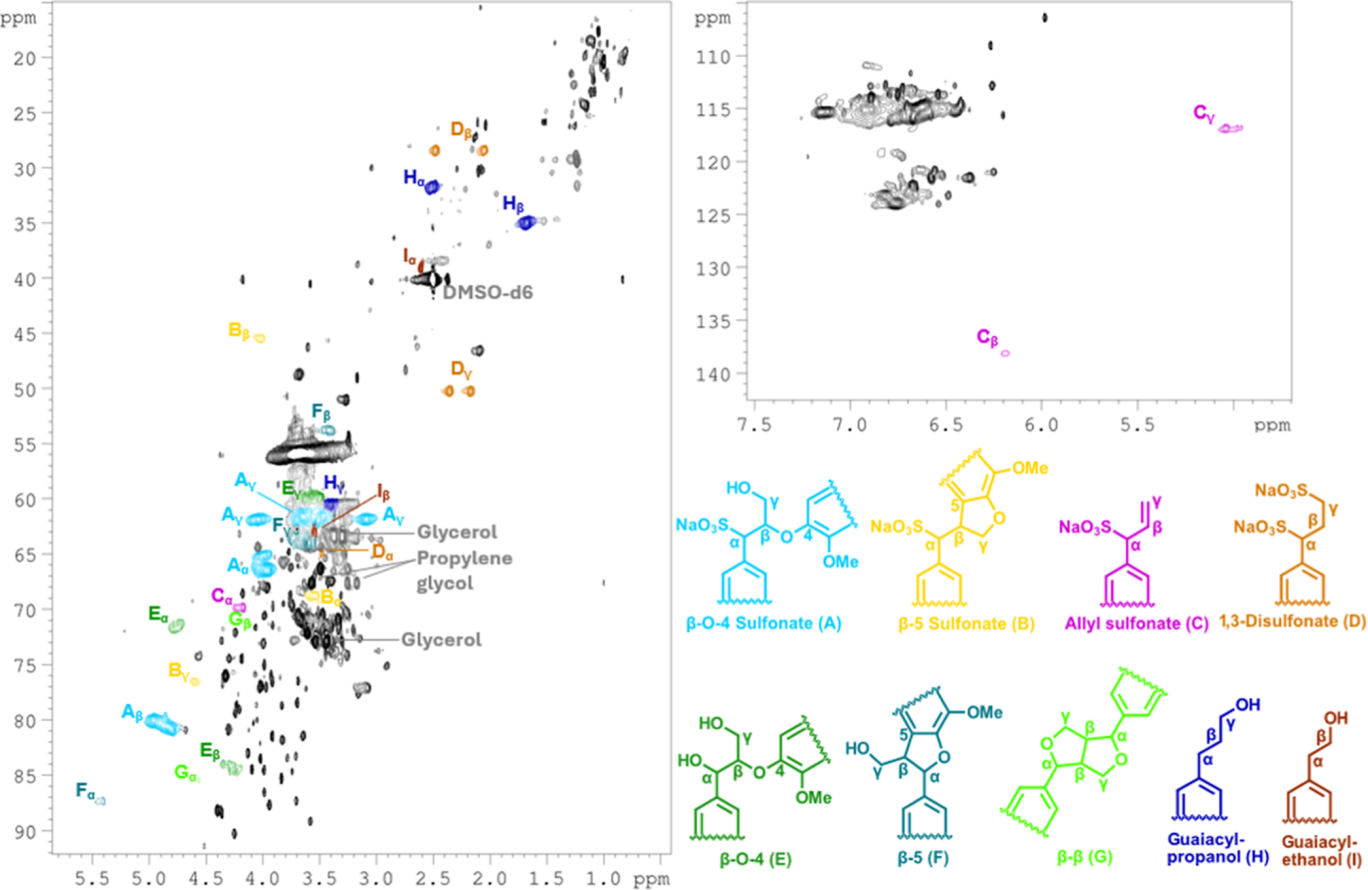
^1^H–^13^C-HSQC-NMR spectrum of sodium lignosulfonate (SM) with signal assignments. Observed structural moieties are β-O-4 sulfonate (A, turquoise), β-5 sulfonate (B, orange), allyl sulfonate (C, purple), 1,3-disulfonate (D, brown), β-O-4 (E, dark green), β-5 (F, dark teal), β–β (G, light green), guaiacylpropanol (H, dark blue), and guaiacylethanol (I, dark brown).

Combining the results from model compound studies with recent literature, a more comprehensive map of the structural elements present in lignosulfonates can be constructed. A total of four sulfonated moieties are presented in the HSQC spectrum of a commercial sodium lignosulfonate, along with the typical native bonding patterns β-O-4 (E), β-5 (F), β–β (G), and guaiacylpropanol (H) (Figure 7). Additionally, guaiacylethanol (I) was observed, representing, to the best of our knowledge, a previously unreported structure in the lignosulfonate literature. TOCSY and HMBC couplings are illustrated in Figures S41–S45 and S50–S51, respectively.

Comparison of the HSQC spectra of fractions F2, F4, and F6, as shown in Figure 8, reveals that all identified signals for characterized lignosulfonate moieties that were discussed above and shown in Figure 7 are present in all the fractions, regardless of molecular weight. Minor differences are seen, such as the appearance of two signals around δ_H_/δ_C_ 5.3/130 ppm, which were detected only in F6. While the exact identity of these signals remains unknown, they may originate from tannin-like structures that are abundant in bark.^50^ Quantitative estimations could not be obtained from current HSQC data alone; however, ongoing work is focused on applying QQ-HSQC and HSQC_0_ techniques to enable quantitative analysis. Regardless, the HSQC spectra can be used to obtain relative ratios between selected signals, for instance, methoxyl and the α- and β-CH-signals from the α-sulfonated β-O-4 moiety (A). These ratios do not express absolute quantification or molar ratio, but they may be used to compare trends in relative signal intensities across the fractions. To this end, the α-signals for the two diastereomers at δ_H_/δ_C_ 4.01/66.0 and 4.02/64.9 ppm were integrated in the 2D HSQC spectra, which revealed an approximate 70:30 diastereomeric ratio for all fractions, including unfractionated SM (Table S5). This does not necessarily correlate to a 70:30 molar ratio of the diastereomeric pairs; however, it means that the ratio is independent of molecular weight. Similarly, methoxy absolute integrals were compared with the β-signals, giving ratios of 92:8, 94:6, and 95:5 for F2, F4, and F6, respectively. Although possibly not statistically significant, it does correlate well with the decreasing sulfonation degree observed in Table 2. Absolute values of the integrals decrease dramatically with increasing molecular weight (Table S5) due to the dependence of molecular tumbling with size, which causes short *T*_2_ relaxation. However, the ratios seem to remain stable across the fractions. Increasing the probe temperature is a way of counteracting the effects of molecular tumbling, and performing the methoxy quantification at an increased 353 K gave absolute integrals in the same order of magnitude, regardless of molecular weight (Table S3).

**Figure 8:**
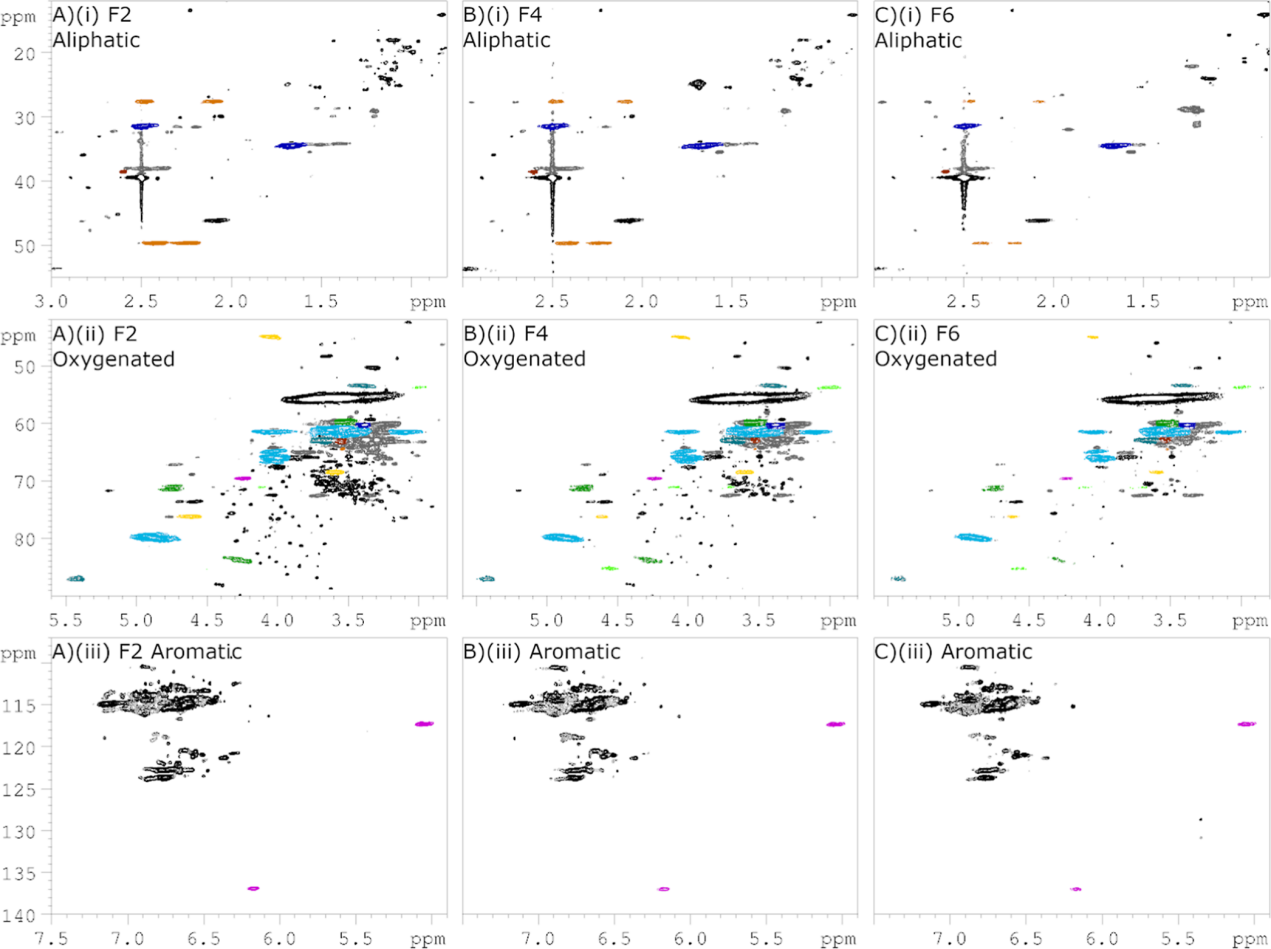
Side-by-side comparisons of fractions F2 (A), F4 (B), and F6 (C). Zoomed-in areas of interest in ^1^H–^13^C HSQC spectra recorded at 298.1 K and 800 MHz: (i): aliphatic region, (ii): oxygenated region, and (iii): aromatic region. Color annotation illustrates signals elucidated in Figure 7 to be present in all fractions, regardless of molecular weight.

### HIC Shows Decreasing Hydrophilicity with Increasing Molecular Weight

The previous sections illustrated that the lignosulfonate fractions differ considerably in molecular weight but have the same bonding patterns and chemical structure. To understand how the balance between size and functional groups influences the interfacial behavior, hydrophobic interaction chromatography (HIC) enables quantification of the relative hydrophobic character of the fractions.

The peak values for the HIC analysis are listed in Figure 9. A trend of decreasing hydrophilicity with increasing molecular weight is observed, indicated by a decrease in peak 1 intensity (from 82 for F2 to 17 for F6) and a corresponding increase in peak 3 (from 4 in F2 to 42 in F6). Over 90% of the elution occurs within peaks 1–3, suggesting that all fractions predominantly exhibit hydrophilic properties. This behavior is likely tied closely to the presence of hydrophilic functional groups and overall molecular conformation.

**Figure 9:**
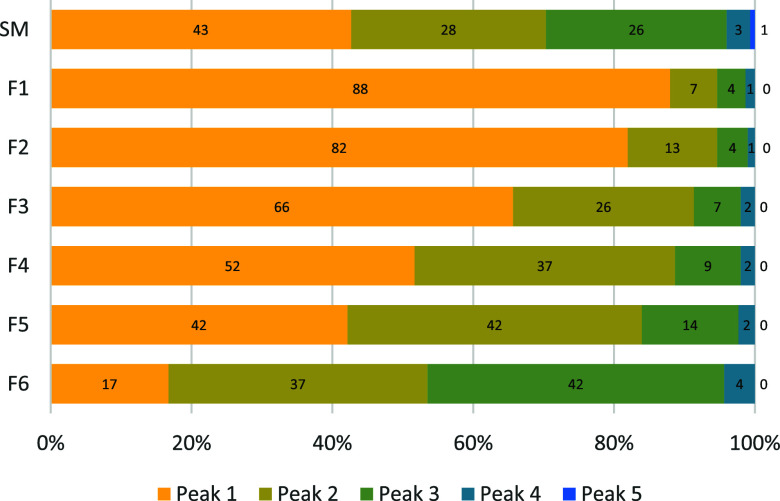
Hydrophobic interaction chromatography (HIC) peak values. HIC peak values for unfractionated lignosulfonate (SM) and molecular weight fractions (F1–F6) show that all samples are predominantly hydrophilic since little and no elution is seen in peaks 4 and 5. The low-molecular-weight fractions (F1 and F2) elute earlier (peaks 1 and 2) and are therefore more hydrophilic compared to the high-molecular-weight fractions (F5 and F6) that elute later (peaks 2 and 3).

Despite only minor variations in the functional group content (Table 2) and largely similar chemical architecture across the fractions (Figure 8), the elution profiles in HIC differ significantly. While HIC is commonly used to assess hydrophobic characteristics, Musl et al.^14^ demonstrated that elution behavior is strongly influenced by the charge-to-size ratio. Later elution in HIC correlates with a lower charge-to-size ratio and is strongly tied to the sulfonation degree and molecular weight. A slight reduction in sulfonation (from 0.56 for F2 to 0.46 for F6) combined with a substantial increase in molecular weight (from 4,500 g/mol for F2 to 78,000 g/mol for F6) would considerably reduce the charge-to-size ratio. Additionally, the larger volume occupied by high-molecular-weight molecules may hinder access to internal functional groups in the molecule, potentially contributing to the delayed elution observed in the HIC chromatogram.

### Hydrodynamic Radius of Lignosulfonate Is Reduced with Decreased Charge-to-Size Ratios

Molecular weight and chemical characteristics of the fractions were addressed in previous sections. It is evident that the charge-to-size ratio plays a key role in determining the hydrophobic character of the lignosulfonate. To further investigate this, the hydrodynamic radius of three fractions and the starting material was determined by measurement of intrinsic viscosity. The results showing molecular size in ultrapure water and higher ionic strength conditions are presented in Figure 10.

**Figure 10:**
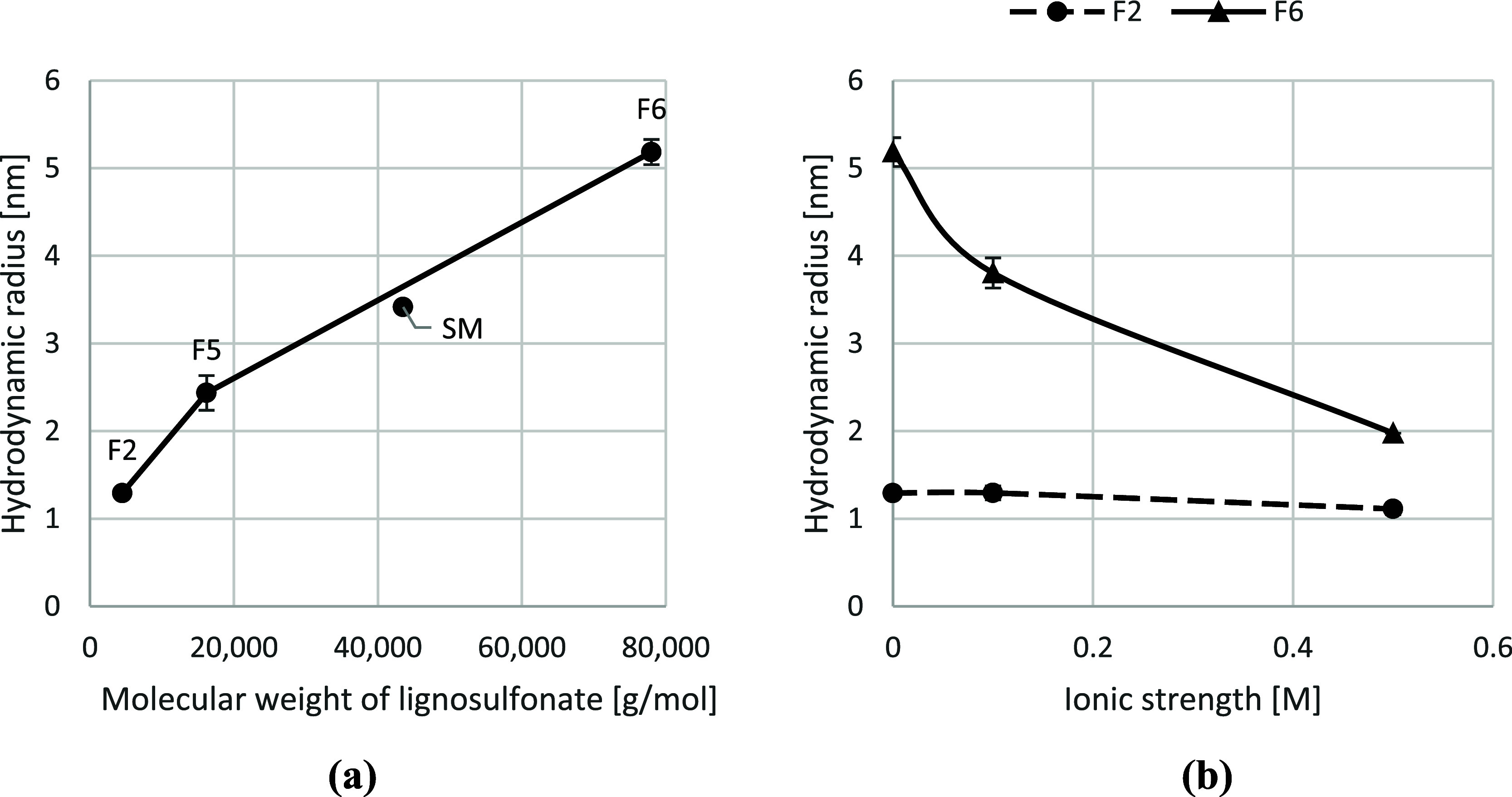
Hydrodynamic radius of lignosulfonate. Figure (a) shows hydrodynamic radius for three selected lignosulfonate fractions and the unfractionated sample (SM) as a function of molecular weight. Figure (b) shows hydrodynamic radius for a low and a high-molecular-weight fraction as a function of ionic strength. (a) Molecular weight: hydrodynamic radius (nm) of lignosulfonate in ultrapure water as a function of molecular weight. Increasing molecular weight corresponds to increased size. (b) Ionic strength: hydrodynamic radius (nm) of lignosulfonate as function of ionic strength. At high ionic strength, hydrodynamic radius is reduced for the high-molecular-weight fraction (F6). No change is seen for the low-molecular-weight fraction (F2).

Figure 10a shows that the hydrodynamic radius of lignosulfonate increases with molecular weight. However, the rate of increase seems to diminish at higher molecular weights, suggesting that larger molecules adopt more compact conformations, likely due to increased chain folding and entanglement. This observation aligns with the expectation that molecular size does not scale linearly indefinitely with molecular weight.

It should be noted that the start material and F6 have high polydispersity in molecular weight, and since the hydrodynamic radius is calculated using the weight-average molecular weight (*M*_w_), these numbers should be interpreted with caution. Nevertheless, the comparison between fractions remains informative. Size measurements for lignosulfonates are scarce in the literature, with reported diameter ranging from 4 to 7 nm.^34–36^ Lower values are typically observed when the water content of swollen lignosulfonates is excluded.^35^ Hydrodynamic radius measured by rheometry as presented here will also encompass the hydration layers around the macromolecule and may therefore be slightly larger than the size measured by other techniques.

The hydrodynamic radius increases with the molecular weight of the lignosulfonate, as expected from the Einstein–Simha relationship, since the intrinsic viscosity scales with the effective hydrodynamic volume of the lignosulfonate molecule. However, this apparent size is influenced by the molecular shape and its interaction with the flow field during rheological measurements. The low-molecular-weight fraction (F2) is expected to be nearly spherical according to a branched polyelectrolyte model proposed by Myrvold.^30^ For the low-molecular-weight fractions, the viscosity-derived radius provides a reasonable approximation of the effective physical size. In contrast, the high-molecular-weight fraction (F6) is expected to exhibit pronounced anisotropy, with an estimated *L*:*D* aspect ratio of approximately 3.5 at low ionic strength, as reported by Vainio et al.^34^ and Qian et al.^35^

When the intrinsic viscosity is determined using a rotational rheometer, the applied shear introduces additional complexity. Elongated particles such as F6 experience hydrodynamic drag forces that promote alignment with the flow direction (sample measured at 250 to 100/s). This alignment reduces the effective hydrodynamic resistance compared to an isotropic orientation, leading to a lower apparent viscosity than would be observed under a random orientation. Consequently, the calculated hydrodynamic radius for F6 is likely underestimated, relative to its random coil dimensions. For F2, the effect of alignment is negligible because its near spherical shape does not exhibit significant orientation dependence.

The interplay between aspect ratio and flow alignment implies that the observed differences in hydrodynamic radius between F2 and F6 reflect not only molecular size but also orientation effects under shear. For elongated lignosulfonate molecules, the reduction in drag upon alignment partially offsets the increase in intrinsic viscosity expected from their anisotropy, resulting in effective radii that are smaller than the true molecular dimensions. Therefore, while the trend of increasing the hydrodynamic radius with molecular weight is robust, the absolute values for highly anisotropic fractions should be interpreted as effective parameters rather than literal physical sizes.

With the increase in ionic strength, a decrease in molecular dimensions is seen for the high-molecular-weight sample, as shown in Figure 10b. For the low-molecular-weight lignosulfonate, no change in molecular dimensions is observed. Under neutral pH conditions in UPW, carboxylic and sulfonic functional groups are deprotonated and thus negatively charged. Repulsion between the negative charges on the molecule causes the polymer to take on a more extended shape in the solution. With the addition of ions, charged groups on the polymers are shielded, and the molecule will therefore take on a more compact structure as described by the branched polyelectrolyte model.^30^ In Figure 10b, a reduction from 5 to 2 nm is seen for F6 when ionic strength is increased to 0.5 M.

It has been proposed that lignosulfonate may possess two hydration layers and that at elevated temperature, the second hydration layer is lost.^32^ The transition temperature where the loss of the hydration layer is seen was found to decrease with ionic strength. Decrease in size with increased ionic strength (as seen in Figure 10b) could partly be due to loss of the hydration layer. However, the size reduction observed in this publication is in the range of 1–3 nm (≫3 Å) and therefore contraction of the macromolecule must also occur.

No decrease in molecular dimension is seen for F2 with increasing ionic strength. The low-molecular-weight sample is small and has a smaller structure that cannot contract to the same extent as the high-molecular-weight sample. A decrease in the hydrodynamic volume will therefore cause a less pronounced response in the radius. The structure might also be slightly different, with less flexible groups or branches that do not allow the molecule to contract and reduce in size.

A clear size difference was observed between the two most extreme fractions, with a notable reduction in the hydrodynamic radius for the high-molecular-weight fraction (F6) under high ionic strength conditions. A larger size is correlated to a decreased charge-to-size ratio and therefore a less hydrophilic molecule. The physicochemical properties of the lignosulfonate fractions, particularly the charge-to-size ratio, play a decisive role in their suitability for different applications. Molecules with a high charge-to-size ratio typically exhibit superior dispersing behavior due to enhanced electrostatic repulsion, whereas those with a lower charge-to-size ratio tend to function more effectively as binders through increased network formation. Additionally, hydrophilicity can influence the dispersing performance in suspensions containing particles with differing surface characteristics, such as hydrophilic versus hydrophobic surfaces.

## Conclusions

The aim of the study was to clarify how chemical structure and molecular weight are intertwined and to improve the understanding of lignosulfonate architecture. Six sodium lignosulfonate fractions with different molecular weights were prepared by a two-step ultrafiltration procedure resulting in molecular weights ranging from 2,200 to 78,000 g/mol with a dispersity of 1.7 to 12.2. Functional group content (degree of sulfonation and phenolic hydroxyl, methoxyl, and carboxylic acids) was found to have few variations between the samples, which was further supported by the NMR analysis of three different fractions showing similar structures. Molecular weight was therefore deemed to be the most significant difference among the fractions. It was found that higher molecular weight corresponded to increased hydrophobicity, as determined by HIC. The purified samples were used to reveal previously unreported structural features and bonding patterns using 2D NMR techniques. Diastereomers of the α-sulfonated β-O-4 bonding pattern were fully characterized, and allyl sulfonate, 1,3-disulfonate, and guaiacylethanol were confirmed as constituents in technical softwood lignosulfonate. The present study complements previous literature by contributing to a more complete map of the structural elements that exist in lignosulfonates. The size of lignosulfonate fractions was estimated by rheometry and found to be consistent with values in the literature. High-molecular-weight lignosulfonate was found to be more affected by changes in ionic strength, as seen by a reduction in size. The charge-to-size ratio of lignosulfonates is an important property influencing their performance in applications such as dispersants and binders.

## Supplementary Material



## Notes

Elemental and organic acids analyses, high-resolution mass spectra, ^1^H and ^31^P NMR spectra, 2D HSQC, HSQC-TOCSY, and HMBC NMR spectra of lignosulfonate fractions, and hydrophobic interaction chromatograms (PDF)
