# Supplementary material for: Unravelling the Complexity of Lignosulfonates—Fractionation and Physicochemical Profiling
Source: Biomacromolecules. 2025 Dec 9;27(1):917–29. doi: 10.1021/acs.biomac.5c02229 (PMC12801188; doi:10.1021/acs.biomac.5c02229)
Supplement: Supplementary file 1 [file bm5c02229_si_001.pdf]

Supporting Information

Unravelling the Complexity of Lignosulfonates – Fractionation and Physicochemical Profiling

Veslemøy Margrethe Selvik[ab], *Finn Lillelund Aachmann*[c], *Carlos Salas-Bringas*[a], *and Vebjørn Eikemo*[a]*

[a] Borregaard ASA, 1701 Sarpsborg, Norway

[b] Ugelstad Laboratory, Department of Chemical Engineering, Norwegian University of Science and Technology (NTNU), 7491 Trondheim, Norway

[c] Norwegian Biopolymer Laboratory (NOBIPOL), Department of Biotechnology and Food Science, NTNU Norwegian University of Science and Technology, 7491 Trondheim, Norway

*Corresponding author, e-mail: vebjorn.eikemo@borregaard.com

Table of Contents

1. Elemental and organic acids analyses....3
2. Methoxyl group quantification with 2D HSQC NMR spectroscopy....7
3. High-resolution mass spectra and chromatograms....14
4. ^{1}H NMR Spectra....16
5. 2D HSQC NMR Spectra....19
6. 2D HSQC-TOCSY NMR Spectra....24
7. 2D HMBC NMR Spectra....30
8. ^{31}P NMR Spectra....33
9. Signal assignment from NMR data....36
10. Hydrophobic interaction chromatography....39

1. Elemental and organic acids analyses

Table S1. Chemical characterization of start material and ultrafiltered fractions F1-F6.

**Component**	***Unit***	**SM**	**F1**	**F2**	**F3**	**F4**	**F5**	**F6**
**Organic S**	*mmol/g*	1.71	1.87	2.12	1.75	1.81	1.71	1.62
**Inorganic S**	*mmol/g*	0.31	0.53	0.12	0.12	0.09	0.09	0.09
**Total S**	*mmol/g*	2.06	2.40	2.25	1.87	1.90	1.81	1.71
**Carbon**	*mmol/g*	34.39	25.56	36.05	39.30	39.13	40.30	42.55
**Na**	*mmol/g*	4.00	6.96	3.48	2.74	2.65	2.52	2.04
**Ca**	*mmol/g*	0.09	0.07	0.04	0.03	0.04	0.03	0.08

Carbon was estimated with a Thermo FlashSmart CHNS/O element analyzer applying a modified Dumas method.
Sodium and calcium content was analyzed by inductively coupled plasma optical emission spectroscopy (ICP-OES) with an iCAP 6000 Series ICP Emission Spectrometer from Thermo Scientific.

Table S2. Organic acids and glycerol content as determined by HPLC-RI.

**Component**	***Unit***	**SM**	**F1**	**F2**	**F3**	**F4**	**F5**	**F6**
**Glycolic acid**	*%*	1.7	3.4	1.0	0.9	0.5	0.6	0.3
**Lactic acid**	*%*	1.1	2.5	0.5	0.3	0.2	0.2	0.2
**Formic acid**	*%*	1.5	2.9	0.8	n.d.	0.2	0.3	0.1
**Acetic acid**	*%*	1.5	4.9	0.9	0.6	0.6	0.5	0.5
**Glycerol**	*%*	0.7	1.3	0.6	0.3	0.1	0.4	0.1
**Total COOH**	*mmol/g*	0.67	2.17	0.51	0.25	0.23	0.25	0.17

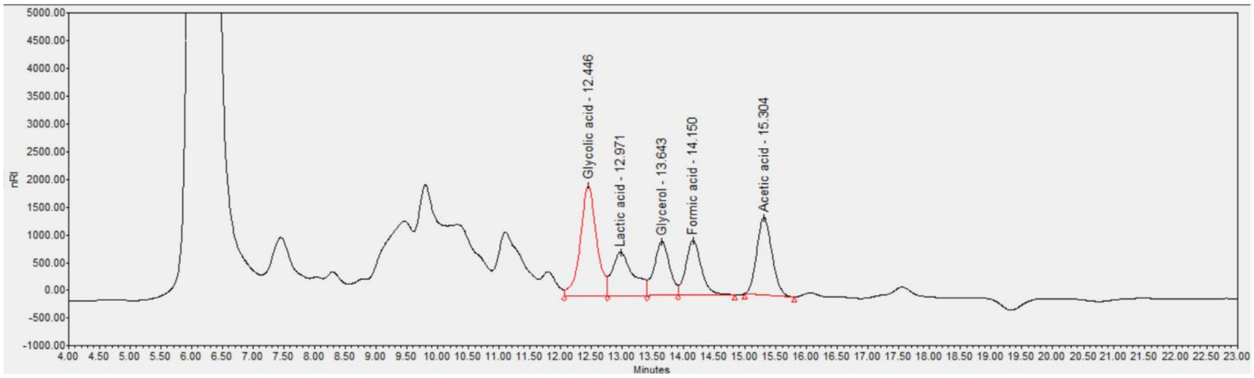

Figure S1. Chromatogram from HPLC-RI analysis of SM.

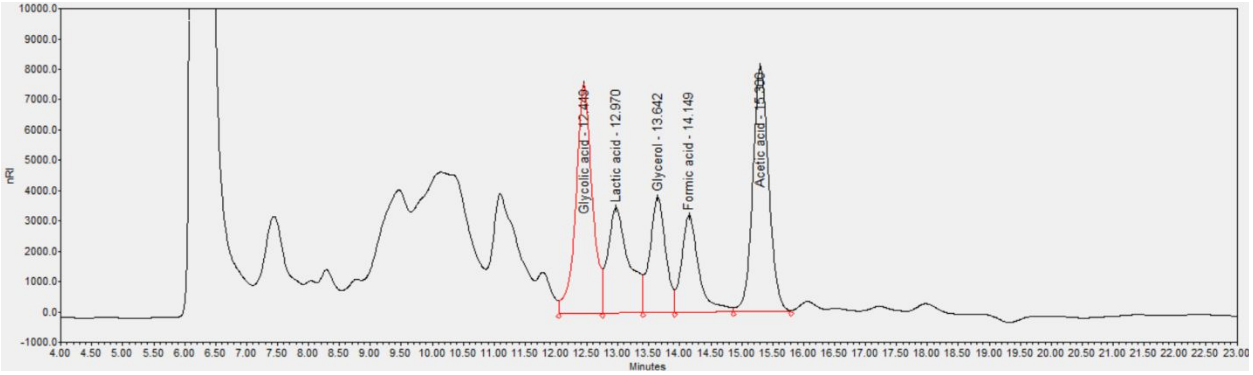

Figure S2. Chromatogram from HPLC-RI analysis of F1.

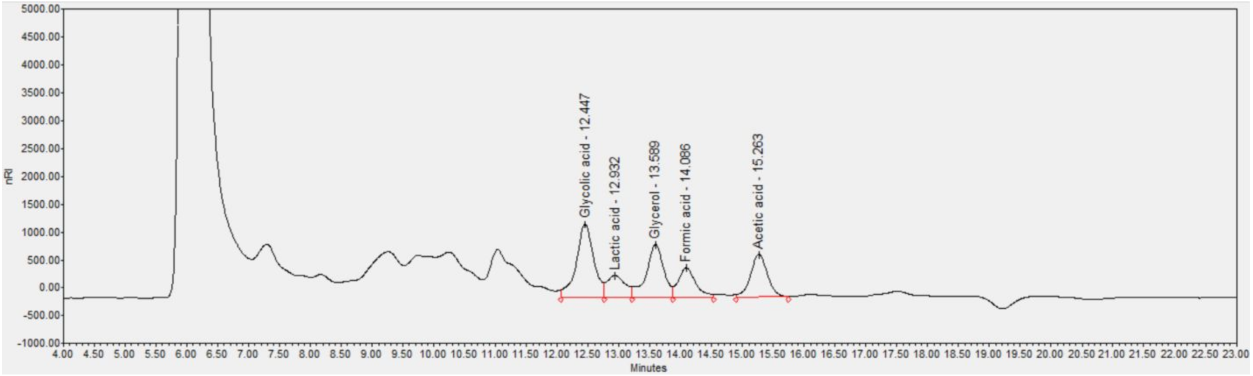

Figure S3. Chromatogram from HPLC-RI analysis of F2.

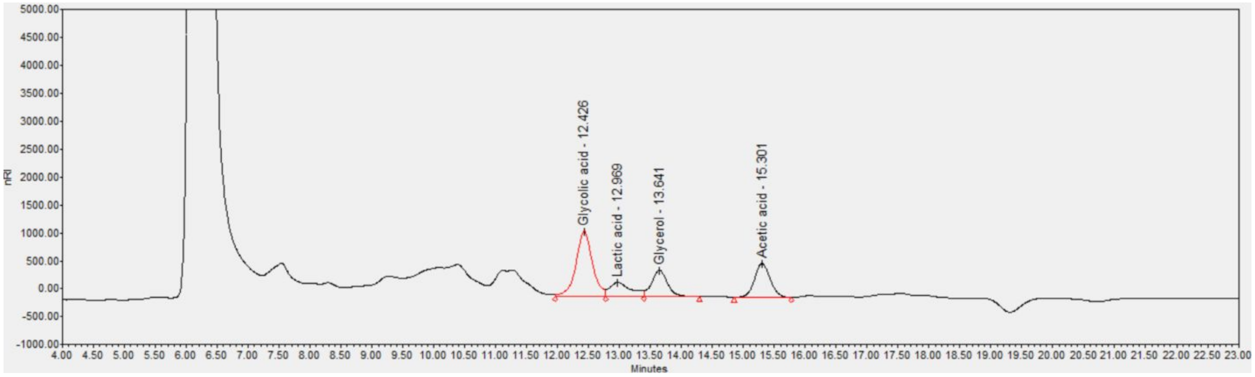

Figure S4. Chromatogram from HPLC-RI analysis of F3.

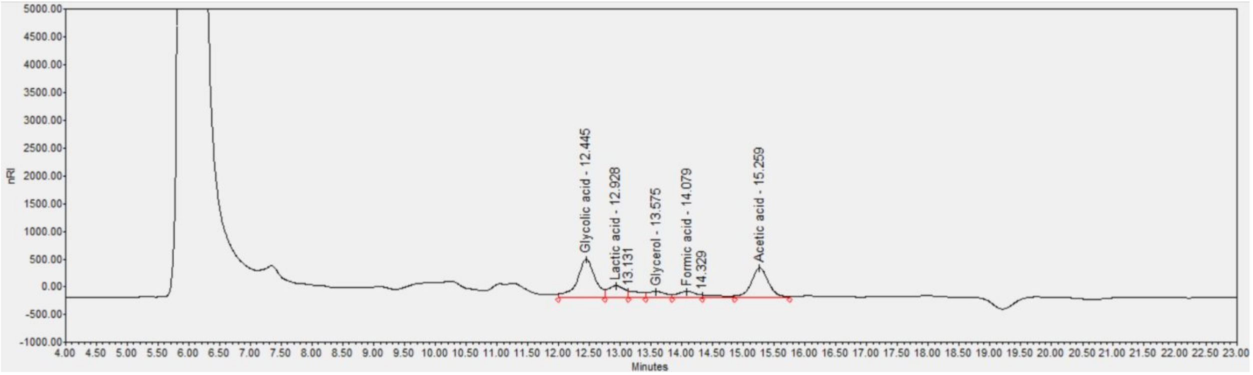

Figure S5. Chromatogram from HPLC-RI analysis of F4.

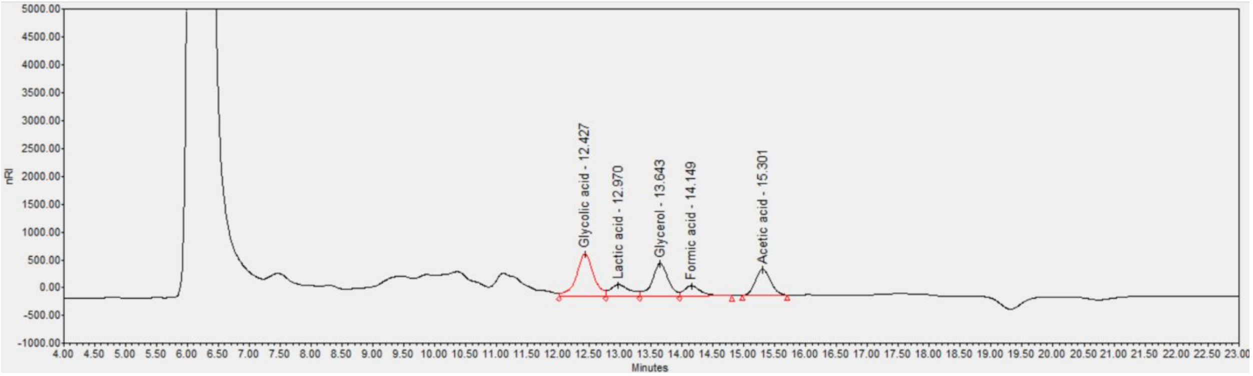

Figure S6. Chromatogram from HPLC-RI analysis of F5.

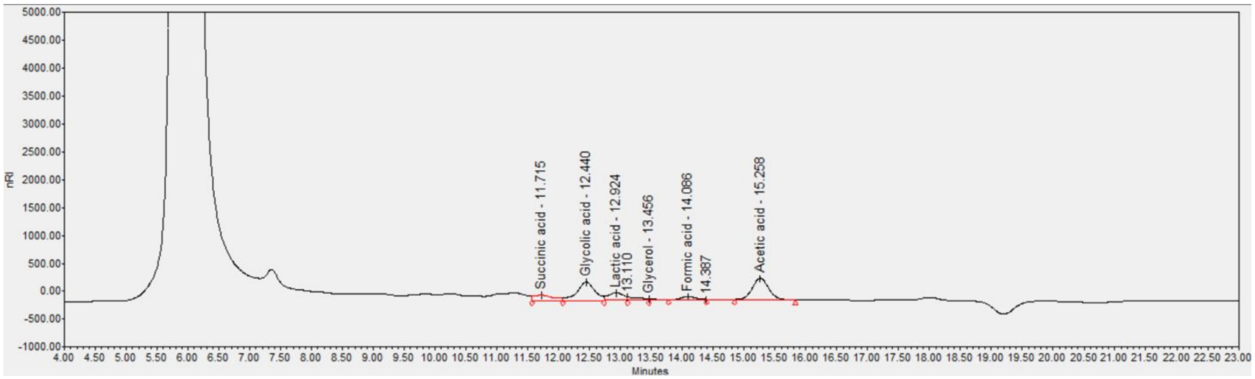

Figure S7. Chromatogram from HPLC-RI analysis of F6.

2. Methoxyl group quantification with 2D HSQC NMR spectroscopy

Table S3. Methoxyl group quantification of lignosulfonate fractions.

Sample	m sample<br>Parallel 1	m sample<br>Parallel 2	Integral F1<br>Parallel 1	Integral F1<br>Parallel 2	MeO in solution<br>Parallel 1	MeO in solution<br>Parallel 2	MeO in LS dry matter
	mg	mg	-	-	mg/g	mg/g	mmol/g
Std 1	15.5	-	88 828	-	1.561	-	3.5
Std 2	20.5	-	118 130	-	2.060	-	3.5
Std 3	25.6	-	147 753	-	2.579	-	3.5
Std 4	30.7	-	176 813	-	3.088	-	3.5
SM	42.6	45.7	115 627	121 392	2.023	2.123	3.1
F1	39.9	44.0	76 144	81 868	1.337	1.436	1.3
F2	22.6	22.3	145 311	145 656	2.538	2.544	3.9
F3	23.1	21.3	160 108	148 790	2.795	2.599	4.1
F4	23.3	23.8	166 800	167 691	2.912	2.927	4.2
F5	22.7	22.1	158 223	156 201	2.763	2.727	4.2
F6	24.1	22.6	146 936	138 718	2.566	2.424	3.6

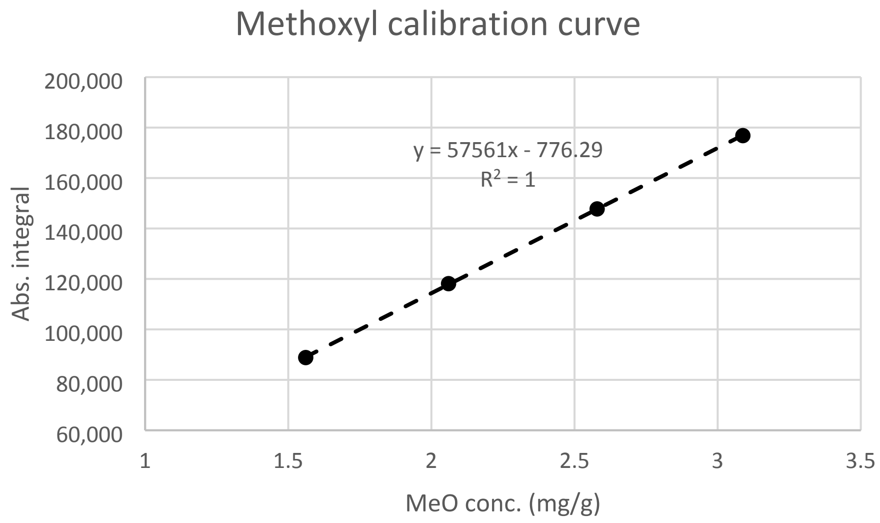

Figure S8. Calibration curve from standard sodium lignosulfonate sample with 3.5 mmol/g methoxyl content.

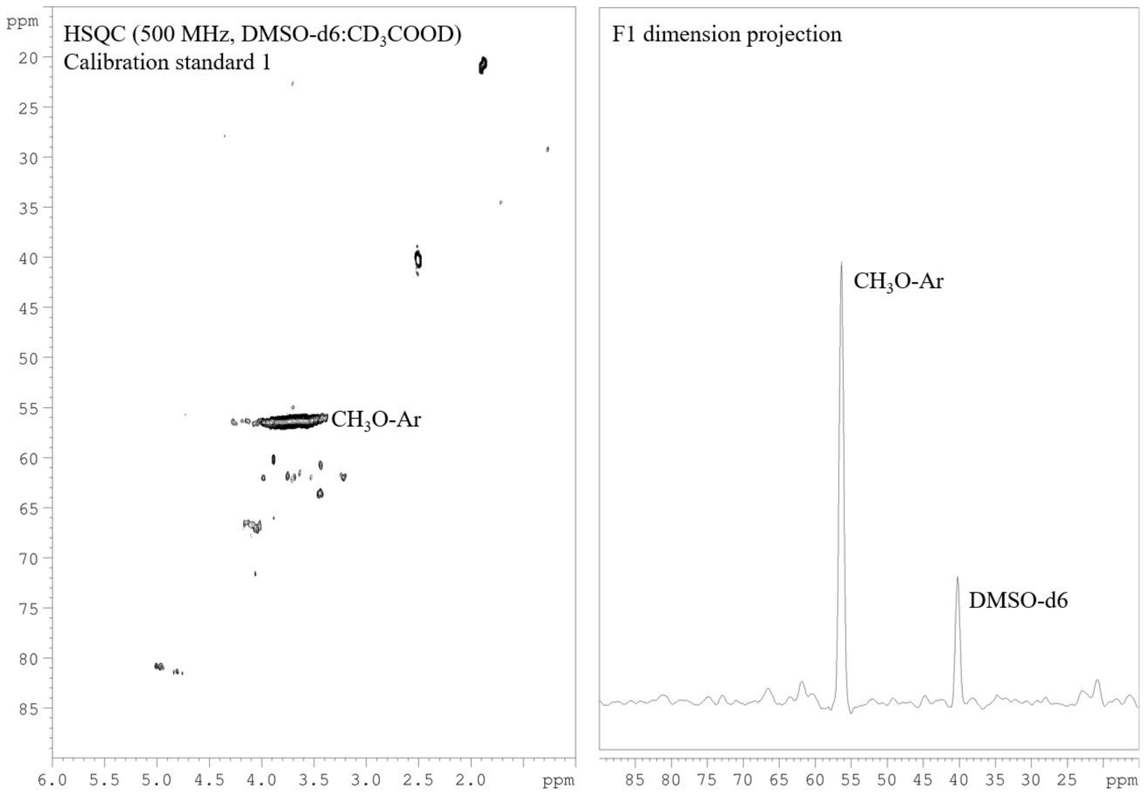

Figure S9. 500.13 MHz ^{1}H-13C HSQC NMR spectrum of calibration standard 1.

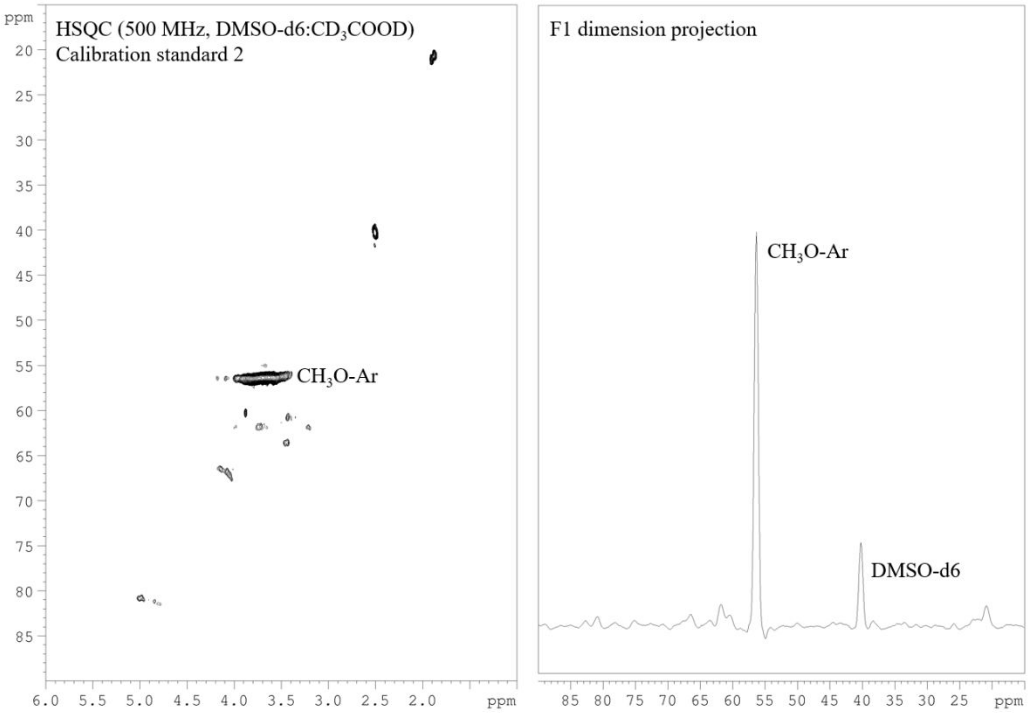

Figure S10. 500.13 MHz ^{1}H-13C HSQC NMR spectrum of calibration standard 2.

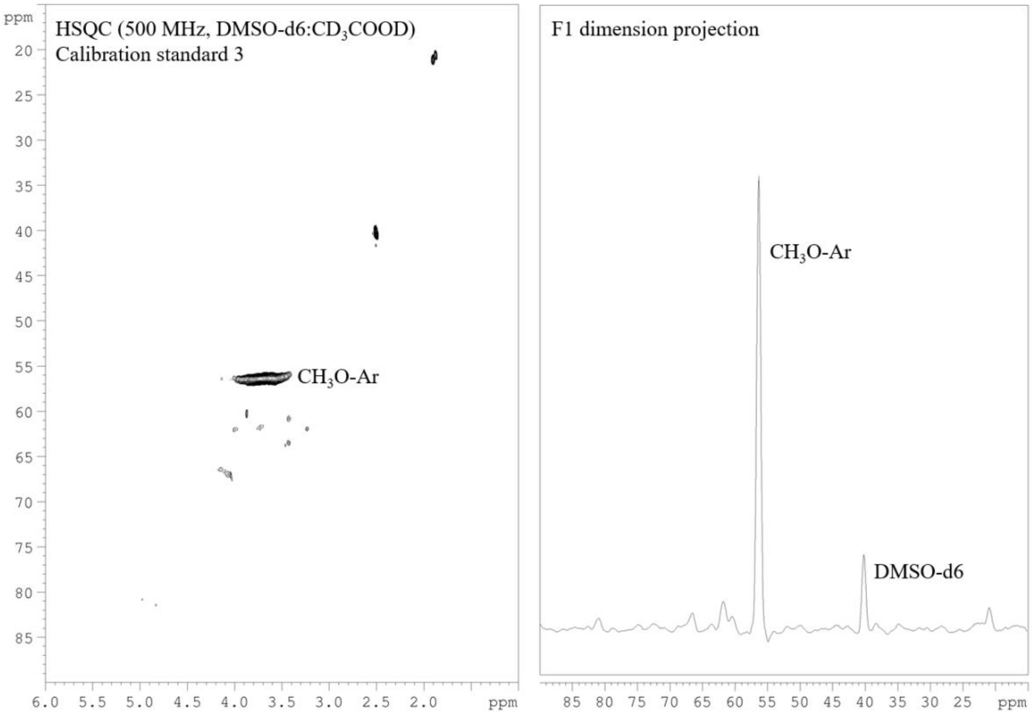

Figure S11. 500.13 MHz ^{1}H-13C HSQC NMR spectrum of calibration standard 3.

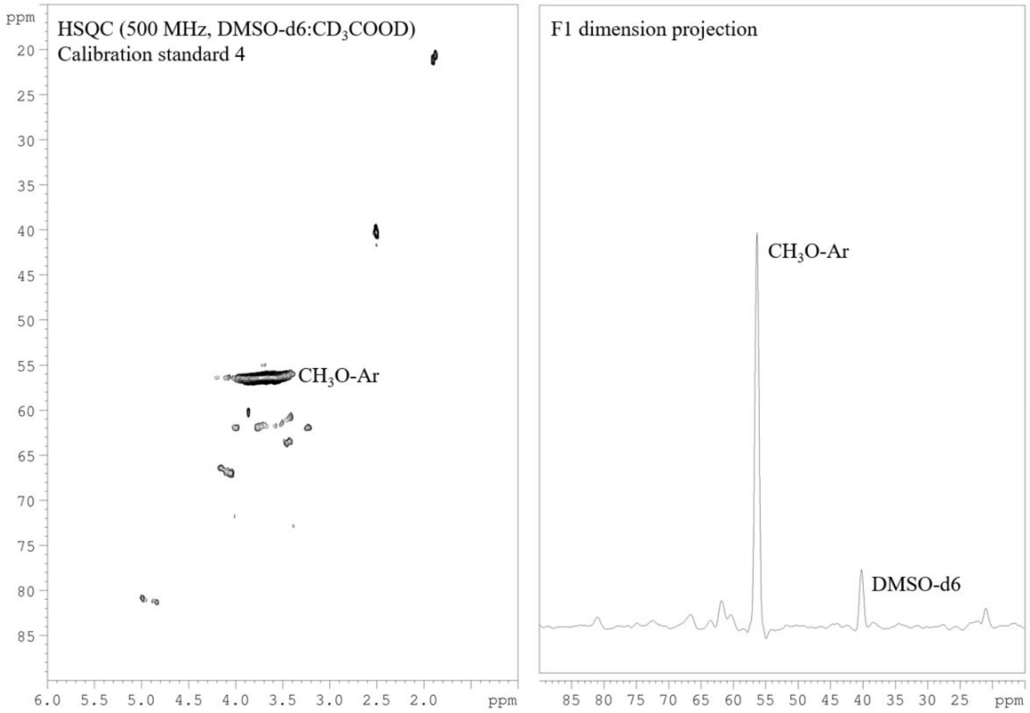

Figure S12. 500.13 MHz ^{1}H-13C HSQC NMR spectrum of calibration standard 4.

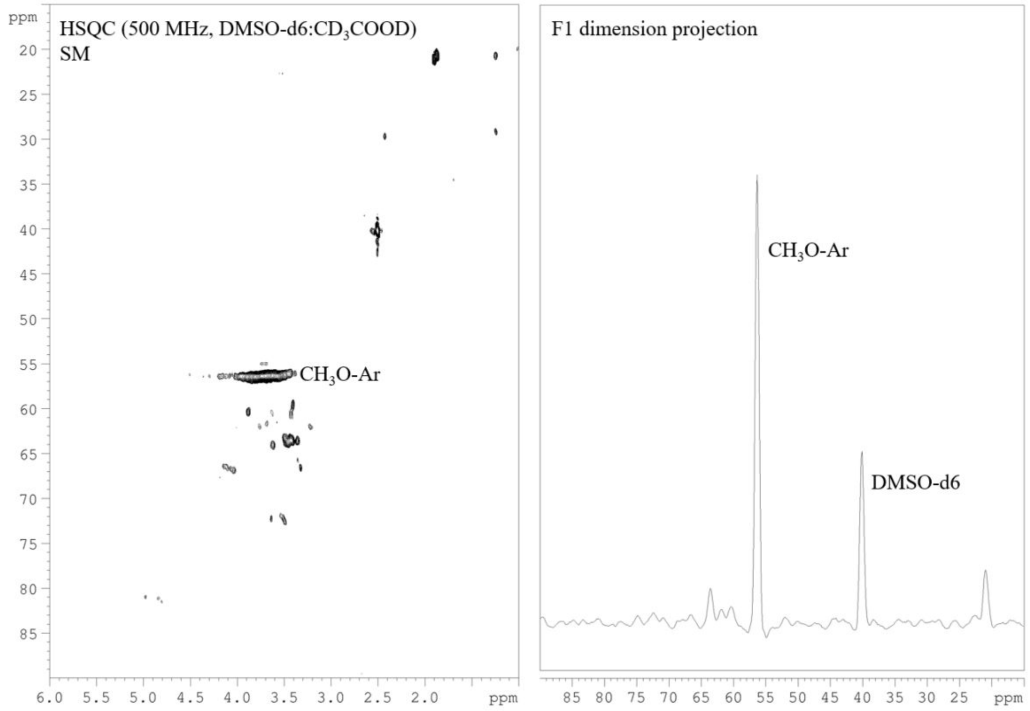

Figure S13. 500.13 MHz ^{1}H-13C HSQC NMR spectrum for methoxyl quantification of SM.

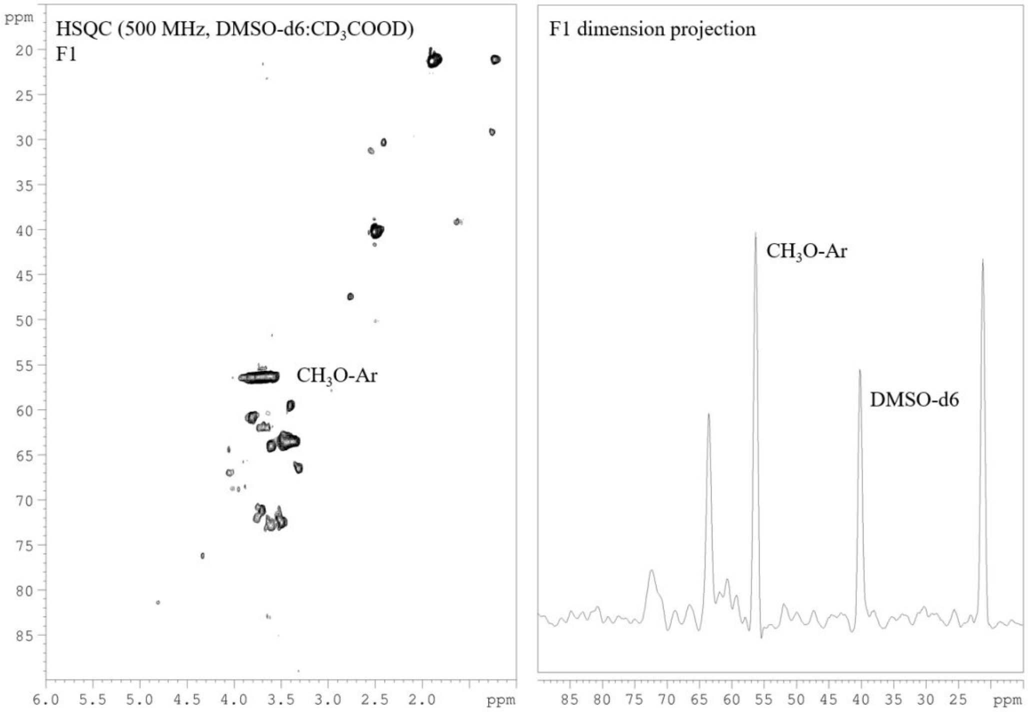

Figure S14. 500.13 MHz ^{1}H-13C HSQC NMR spectrum for methoxyl quantification of F1.

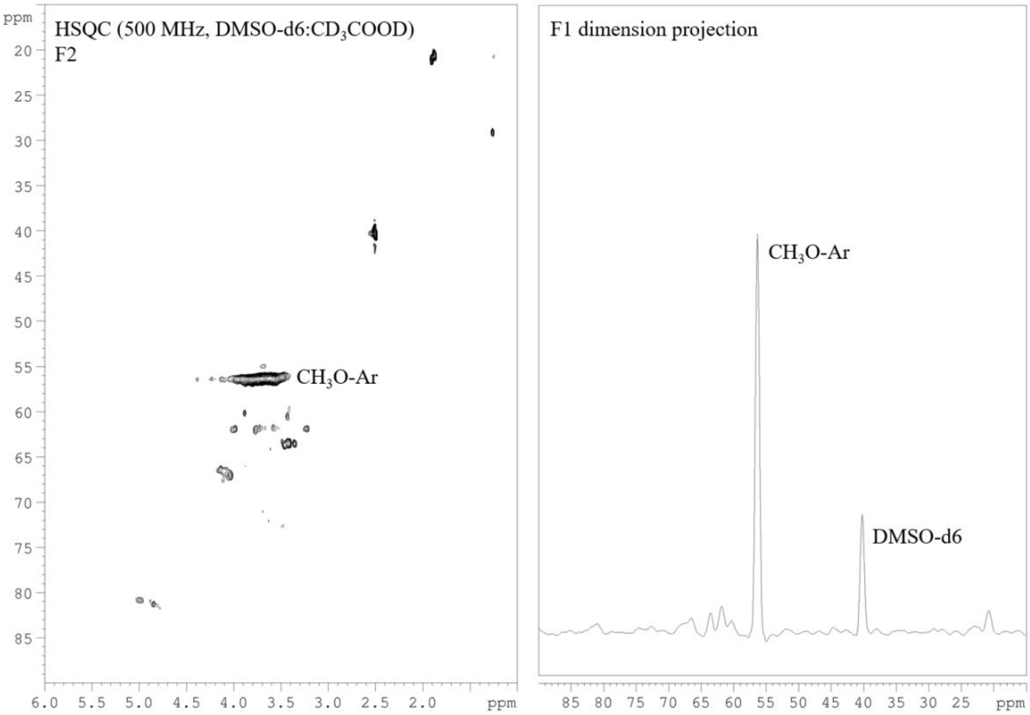

Figure S15. 500.13 MHz ^{1}H-13C HSQC NMR spectrum for methoxyl quantification of F2.

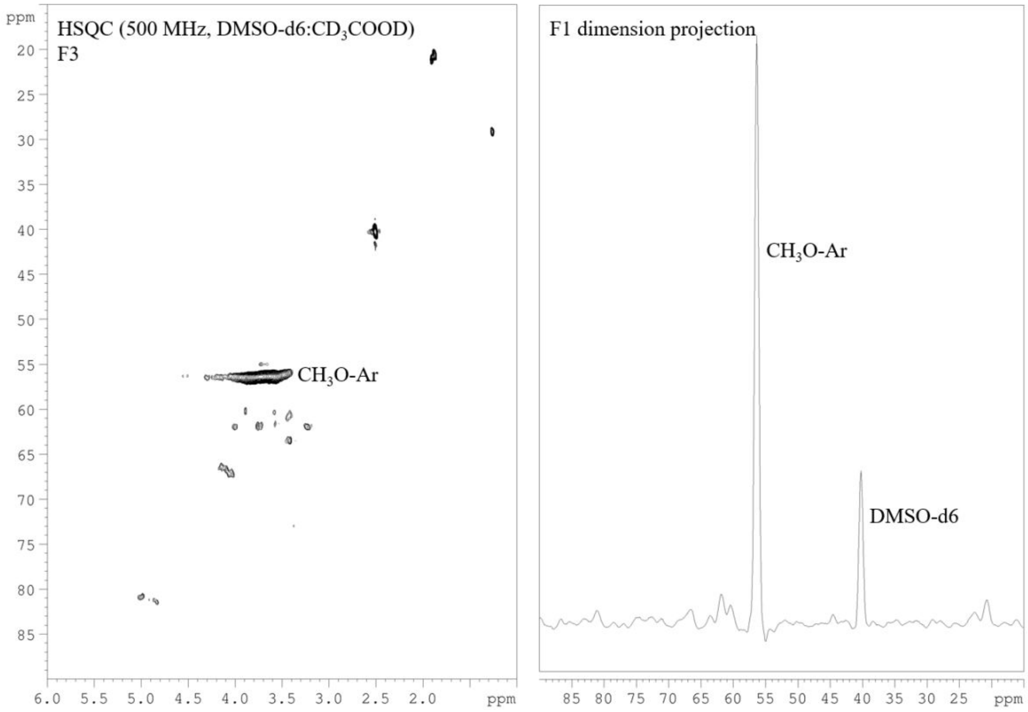

Figure S16. 500.13 MHz ^{1}H-13C HSQC NMR spectrum for methoxyl quantification of F3.

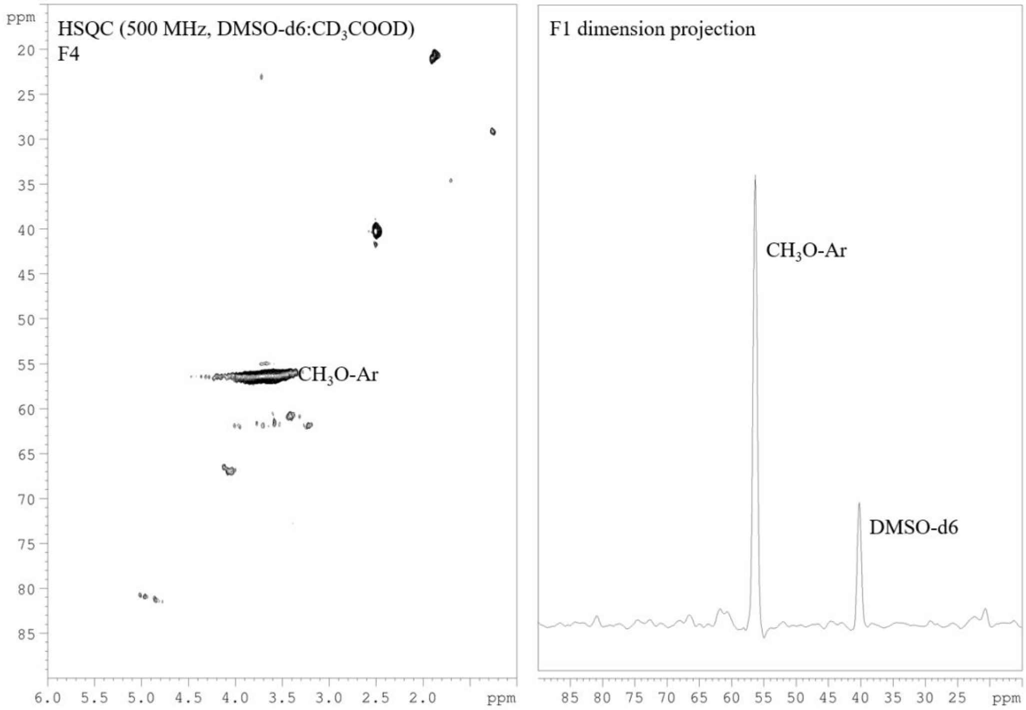

Figure S17. 500.13 MHz ^{1}H-13C HSQC NMR spectrum for methoxyl quantification of F4.

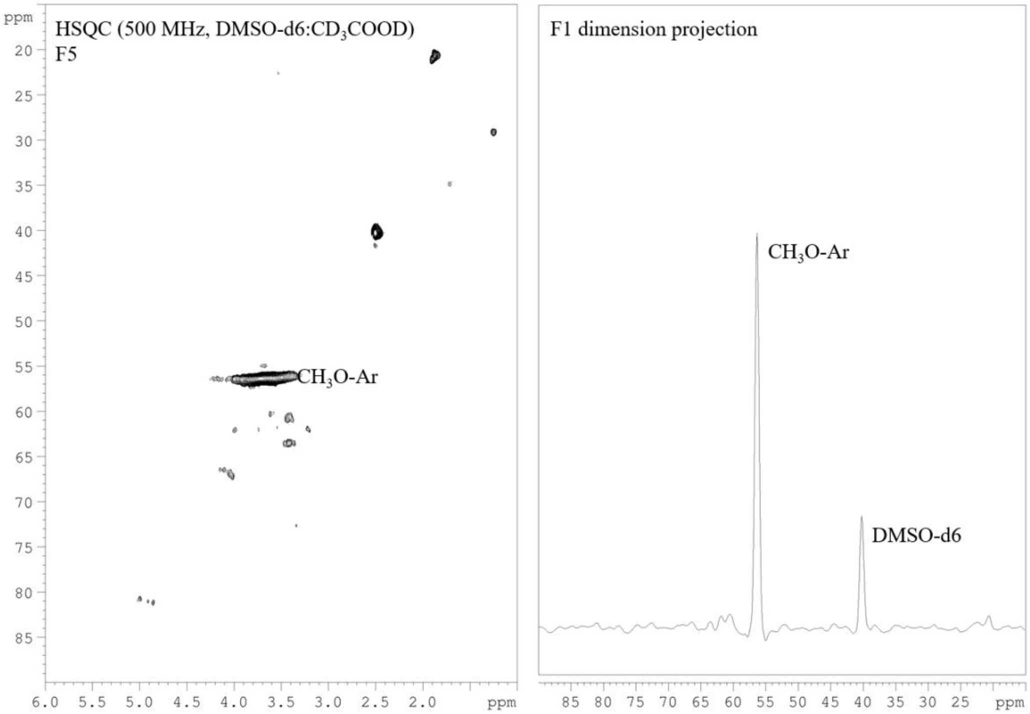

Figure S18. 500.13 MHz ^{1}H-13C HSQC NMR spectrum for methoxyl quantification of F5.

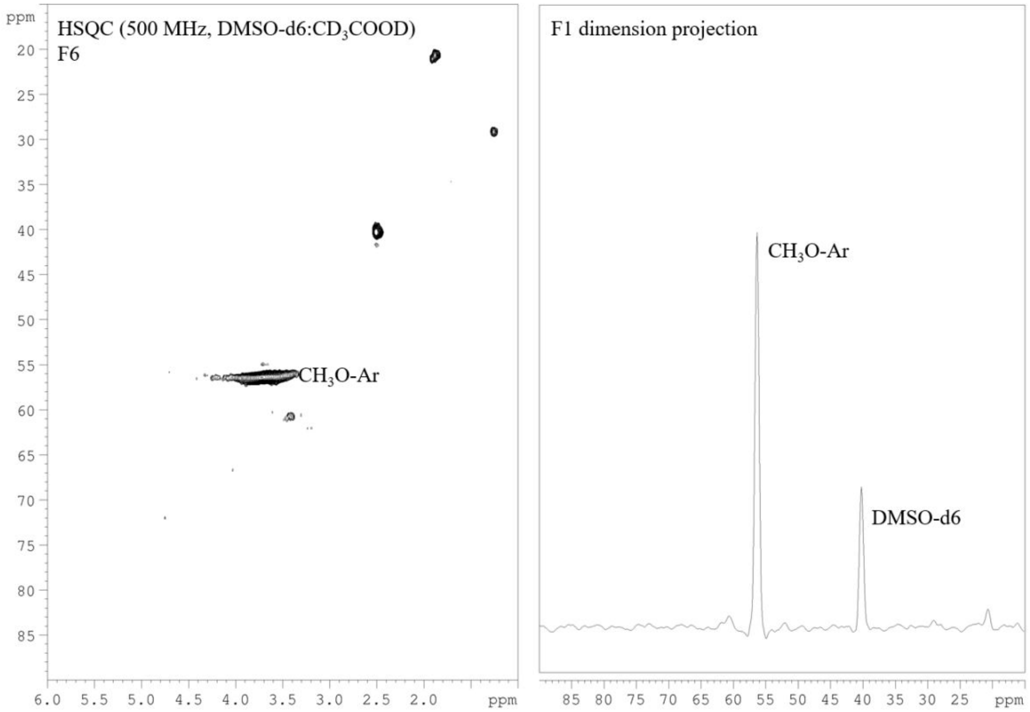

Figure S19. 500.13 MHz ^{1}H-13C HSQC NMR spectrum for methoxyl quantification of F6.

3. High-resolution mass spectra and chromatograms

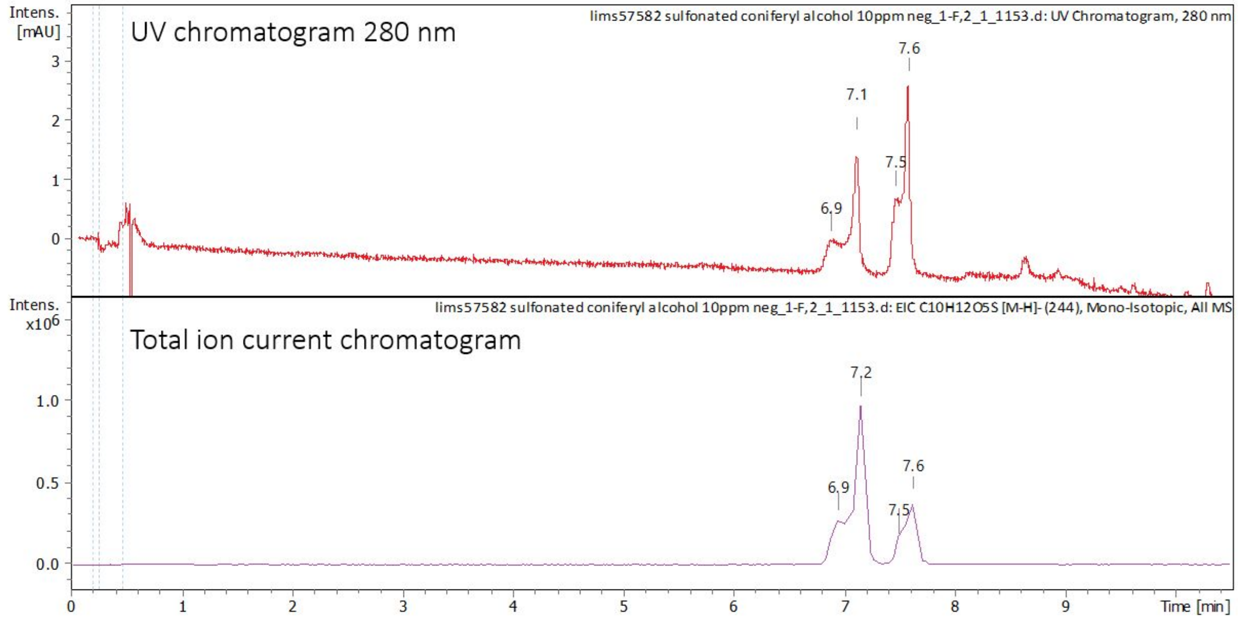

Figure S20. Chromatogram from LC-HRMS analysis of crude reaction mixture from sulfonation of coniferyl alcohol (**4**).

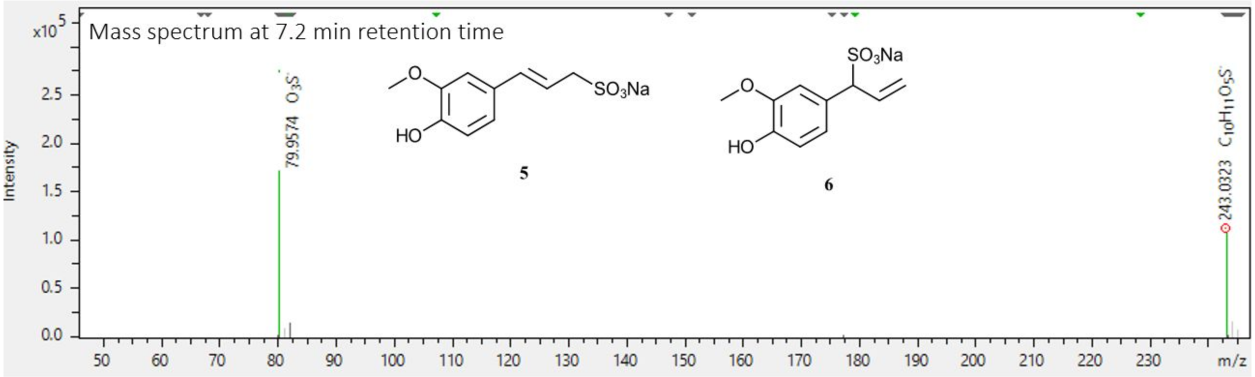

Figure S21. Mass spectrum at 7.2 min retention time from the chromatogram of crude reaction mixture from sulfonation of coniferyl alcohol (**4**).

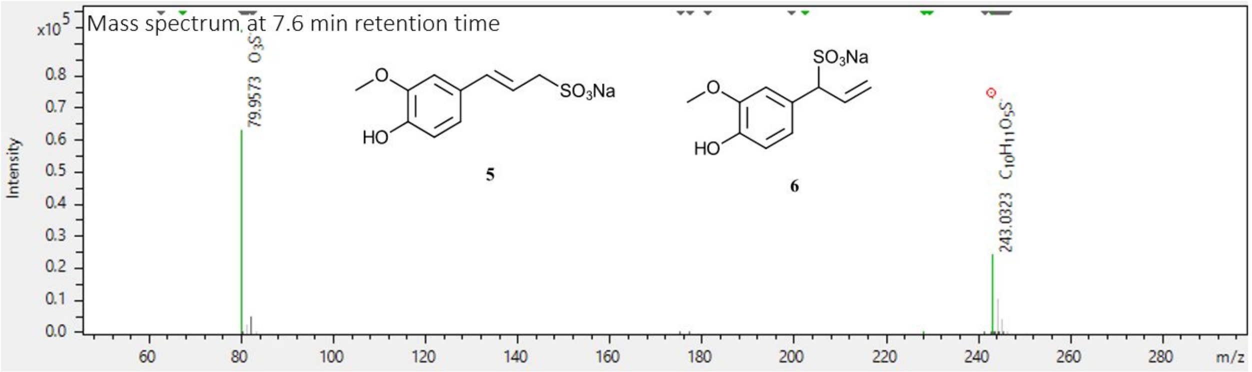

Figure S22. Mass spectrum at 7.6 min retention time from the chromatogram of crude reaction mixture from sulfonation of coniferyl alcohol (**4**).

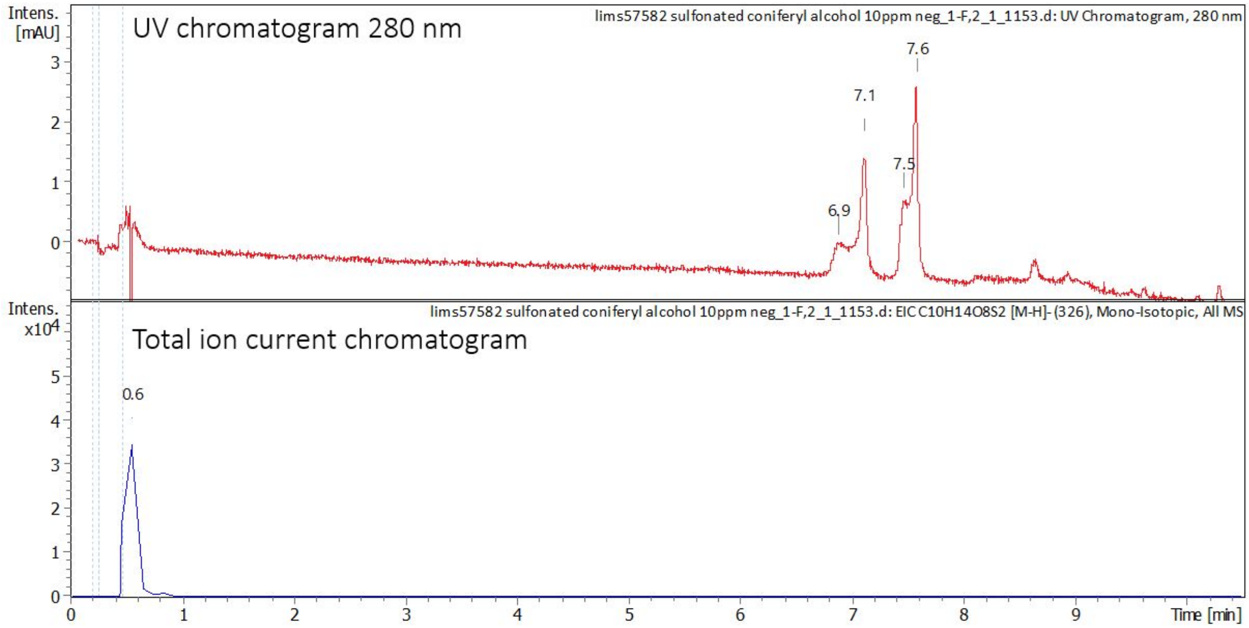

Figure S23. Chromatogram in selected-ion monitoring at 325 m/z from LC-HRMS analysis of crude reaction mixture from sulfonation of coniferyl alcohol (**4**).

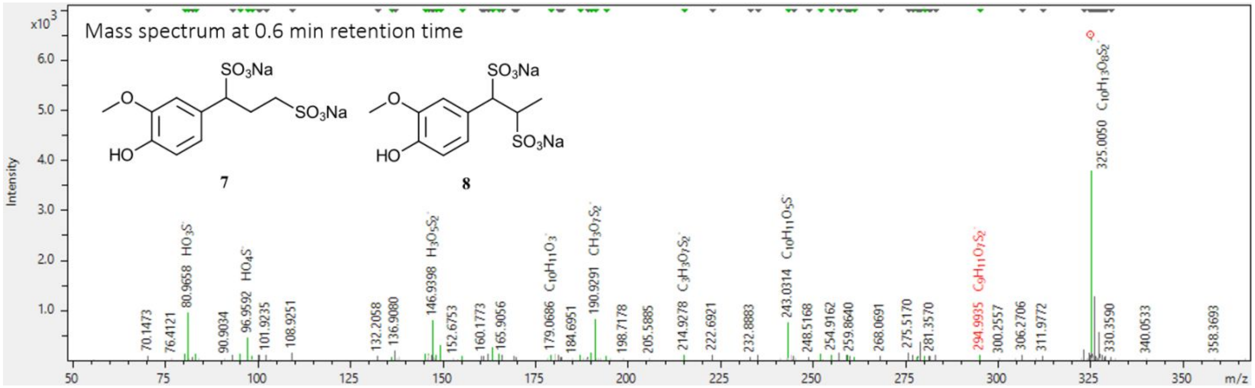

Figure S24. Chromatogram in selected-ion monitoring at 325 m/z from LC-HRMS analysis of crude reaction mixture from sulfonation of coniferyl alcohol (**4**).

4. ^{1}H NMR Spectra

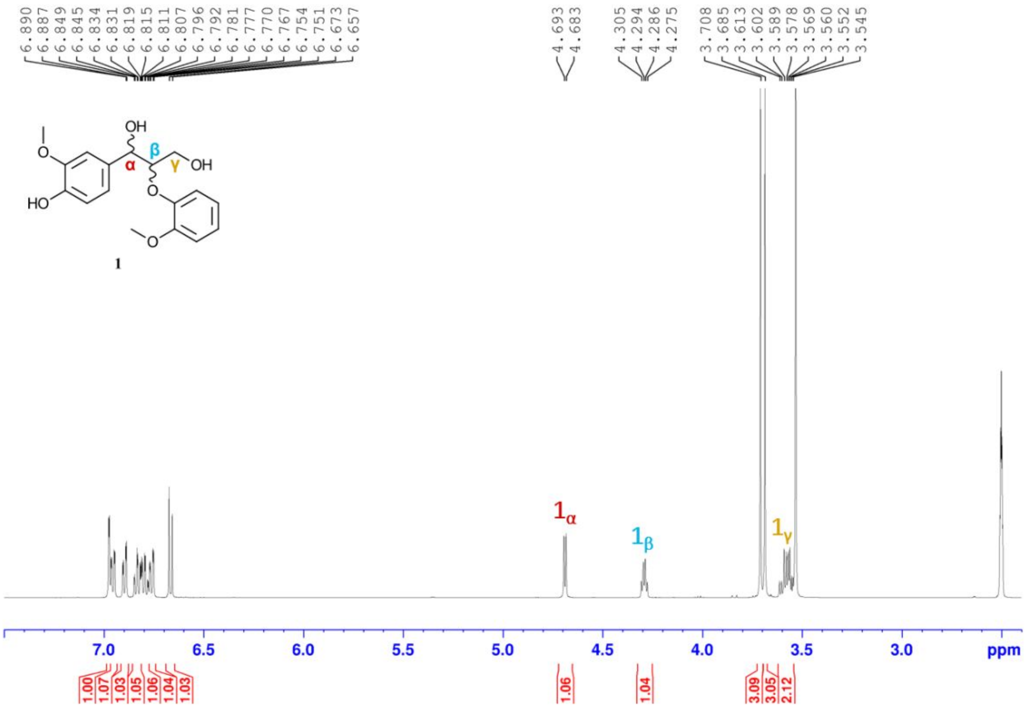

Figure S25. 500.13 MHz ^{1}H NMR spectrum of β-O-4 model.

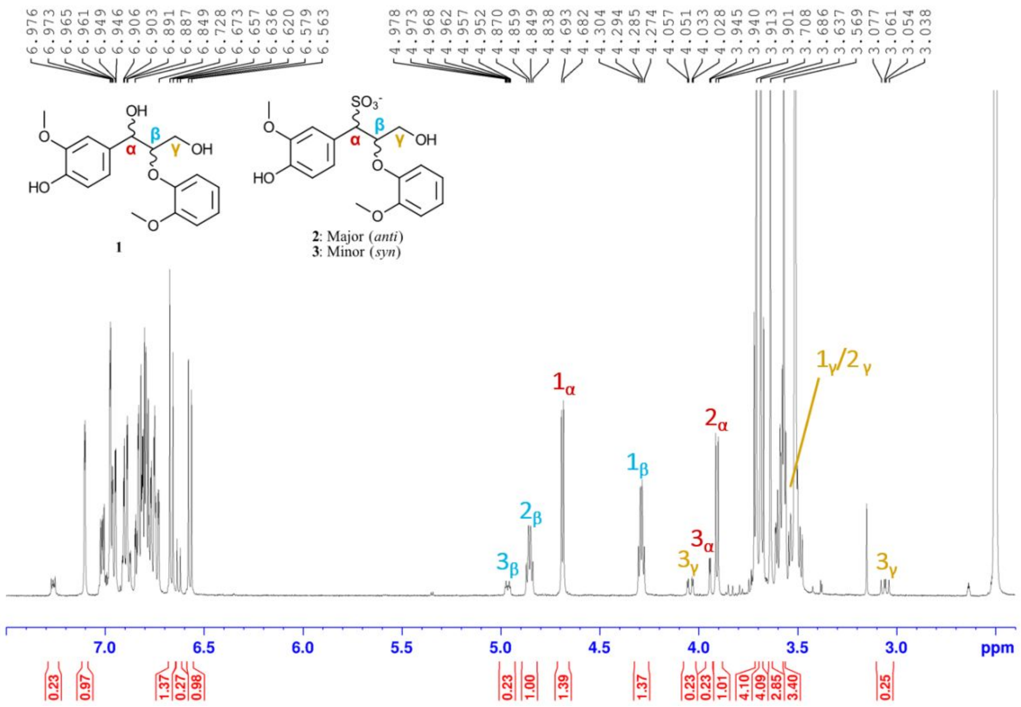

Figure S26. 500.13 MHz ^{1}H NMR spectrum of β-O-4 model.

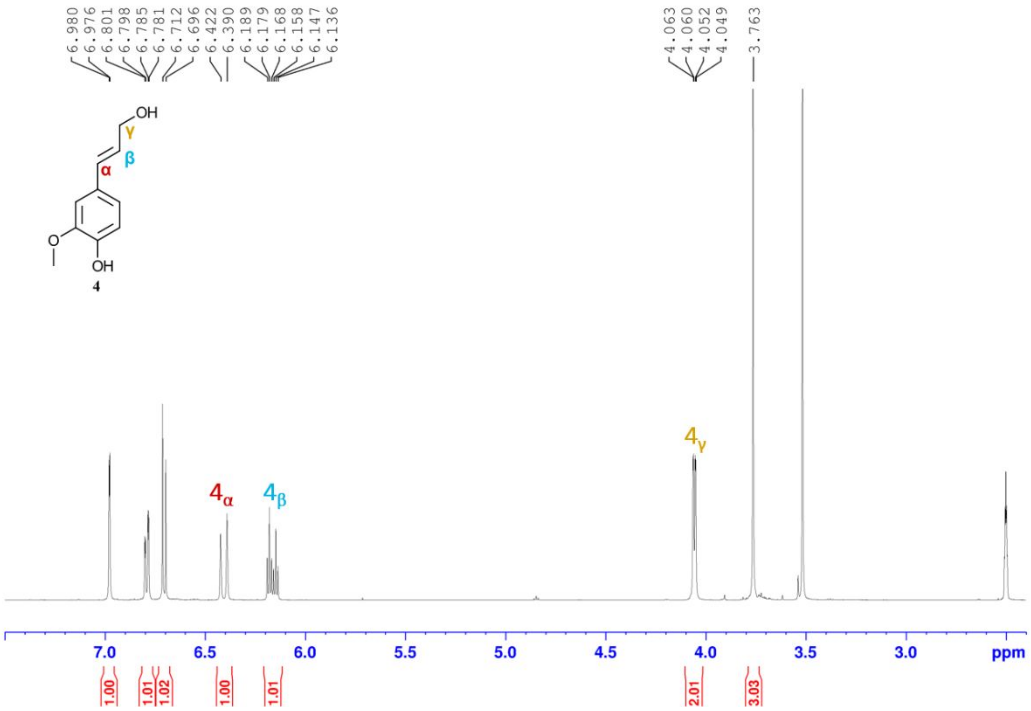

Figure S27. 500.13 MHz ¹H NMR spectrum of coniferyl alcohol (**4**).

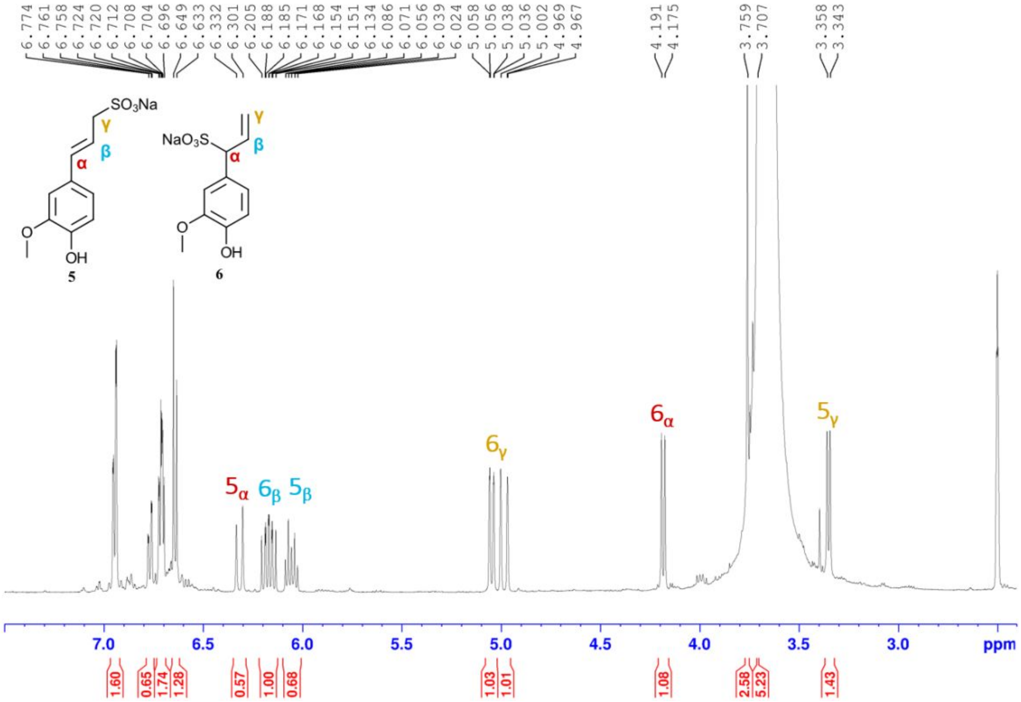

Figure S28. 500.13 MHz ¹H NMR spectrum of sulfonated coniferyl alcohol (**4**).

5. 2D HSQC NMR Spectra

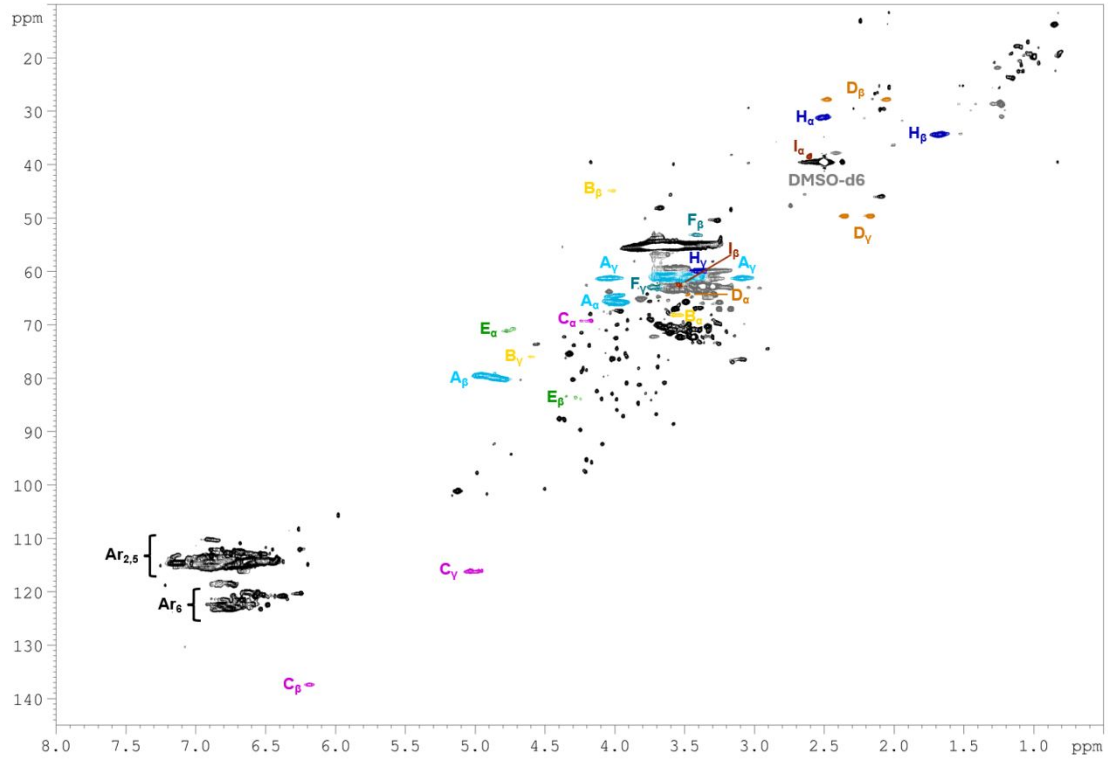

Figure S29. 800.03 MHz ^{1}H-^{13}C HSQC NMR spectrum of starting sodium lignosulfonate.

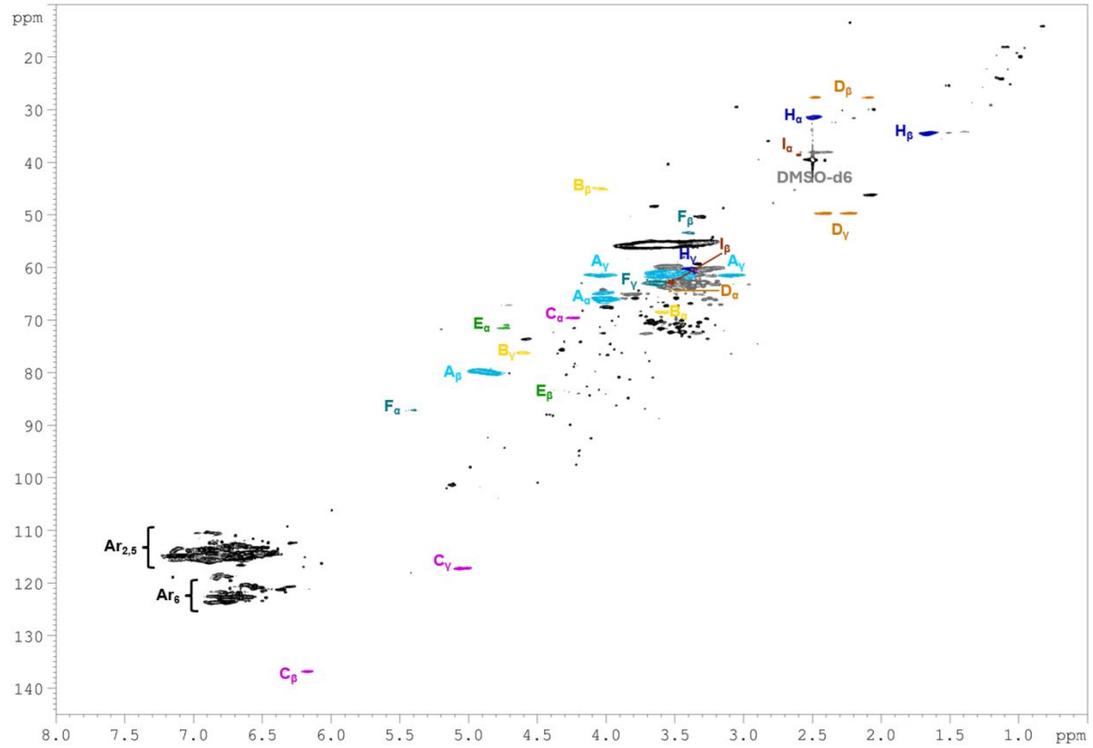

Figure S30. 800.03 MHz ^{1}H-^{13}C HSQC NMR spectrum of fraction F2.

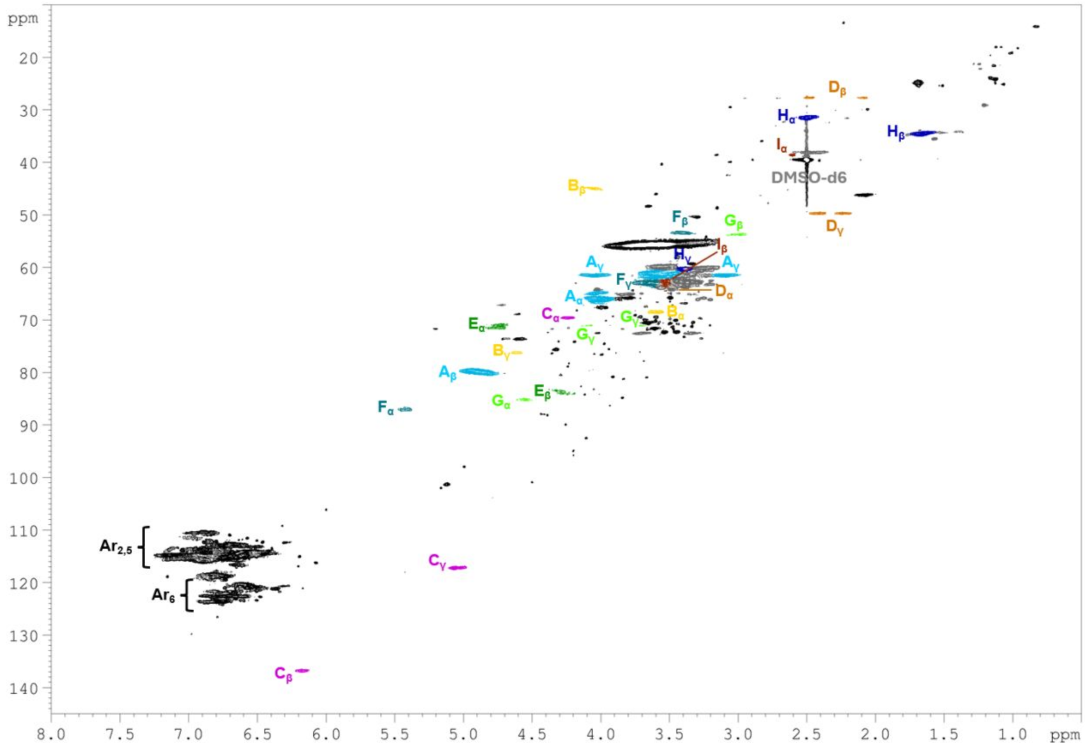

Figure S31. 800.03 MHz ^{1}H-^{13}C HSQC NMR spectrum of fraction F4.

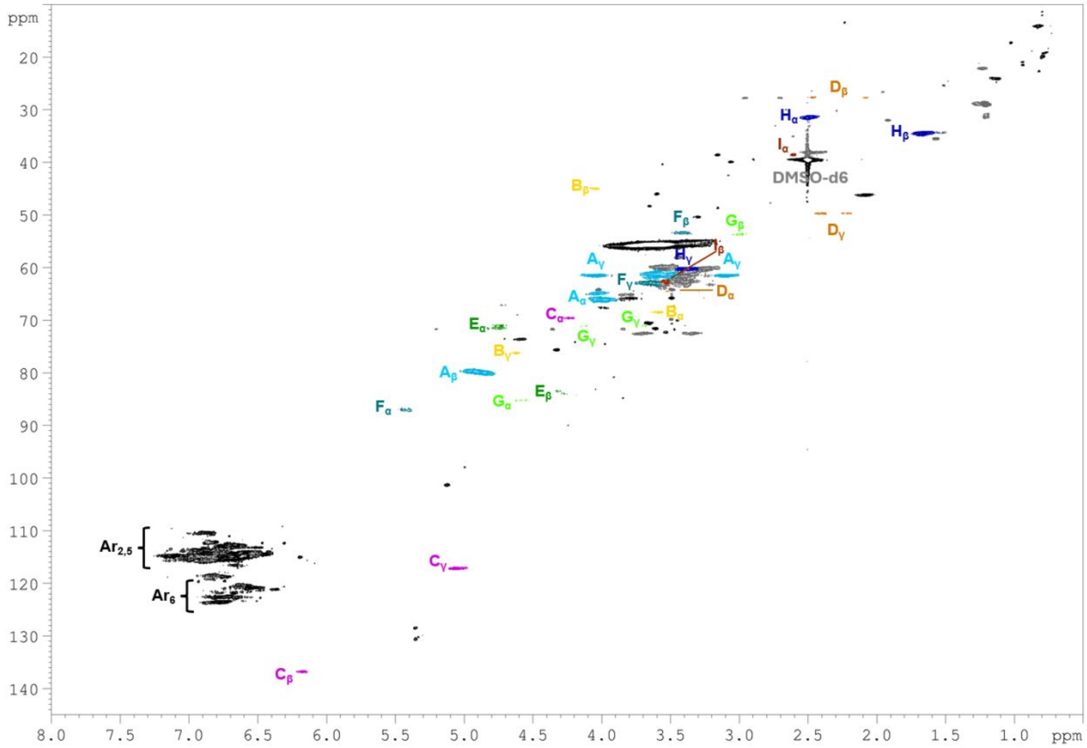

Figure S32. 800.03 MHz ^{1}H-^{13}C HSQC NMR spectrum of fraction F6.

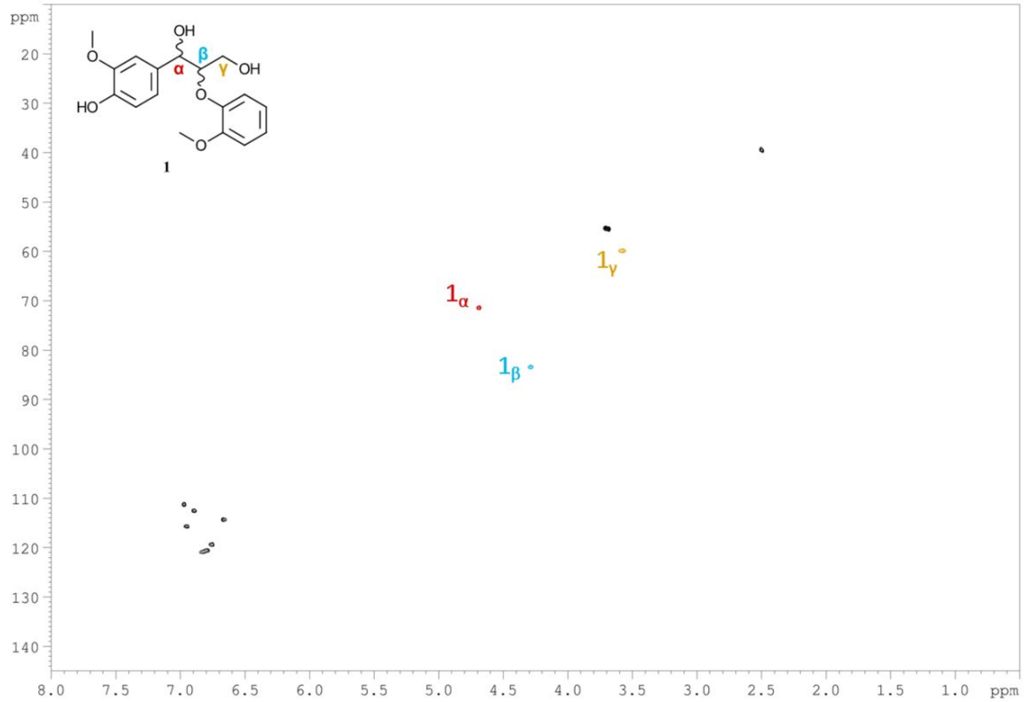

Figure S33. 500.13 MHz ^1H-^{13}C HSQC NMR spectrum of β-O-4 model **1**.

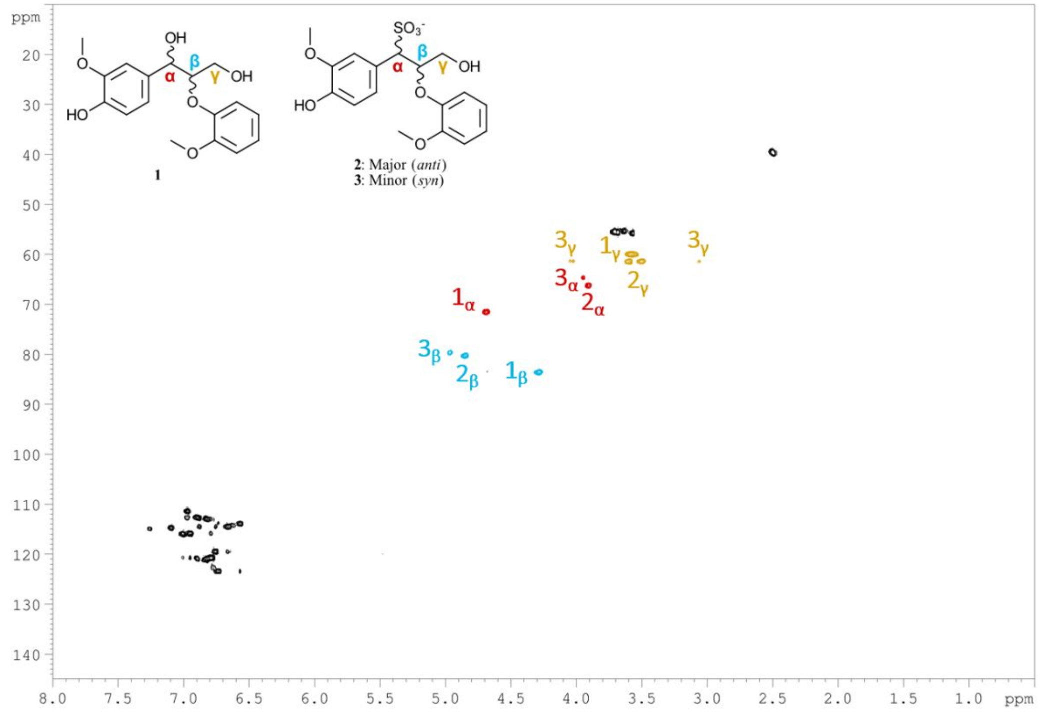

Figure S34. 500.13 MHz ^{1}H-^{13}C HSQC NMR spectrum of sulfonated β-O-4 model **2**.

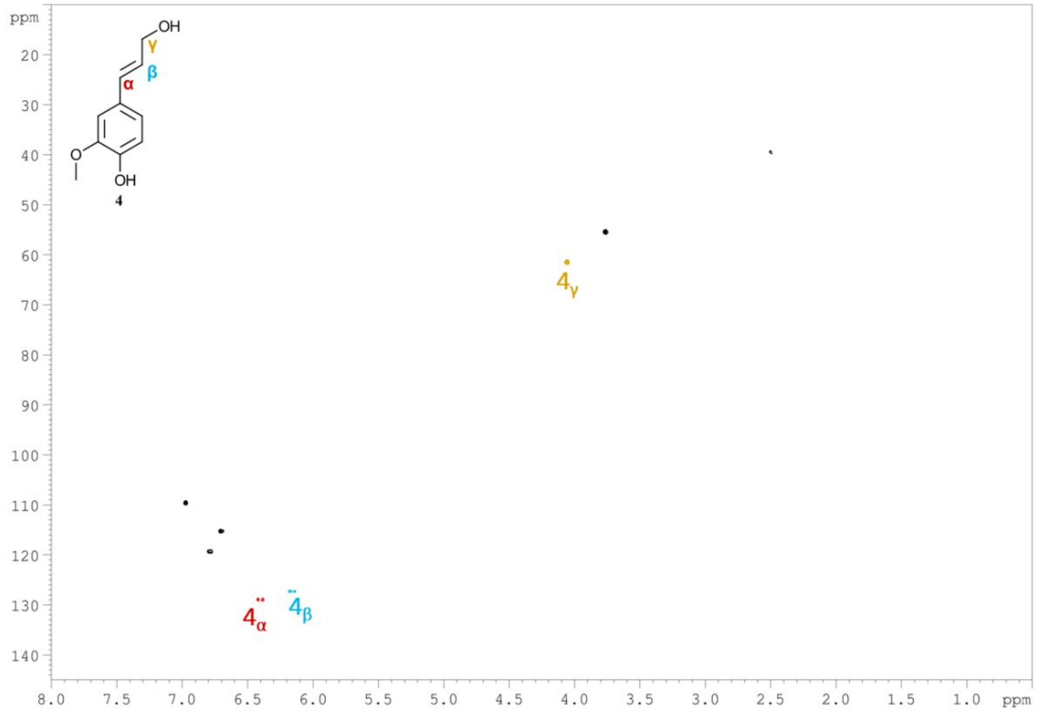

Figure S35. 500.13 MHz ^{1}H-^{13}C HSQC NMR spectrum of coniferyl alcohol.

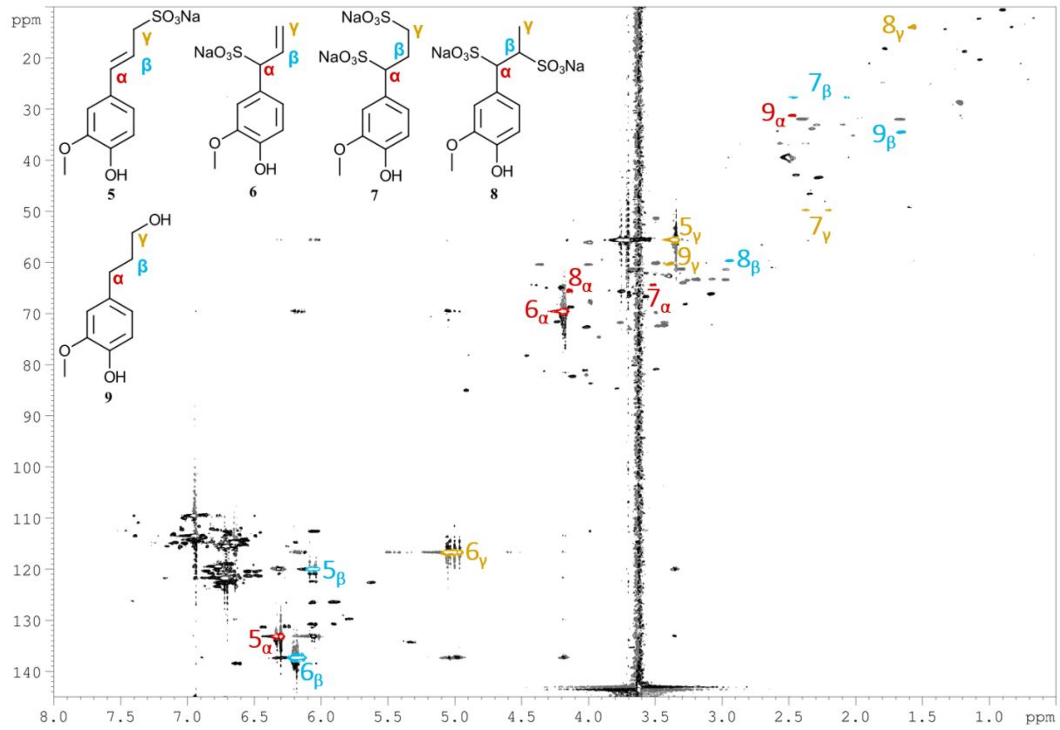

Figure S36. 500.13 MHz ^{1}H-^{13}C HSQC NMR spectrum of sulfonated coniferyl alcohol.

6. 2D HSQC-TOCSY NMR Spectra

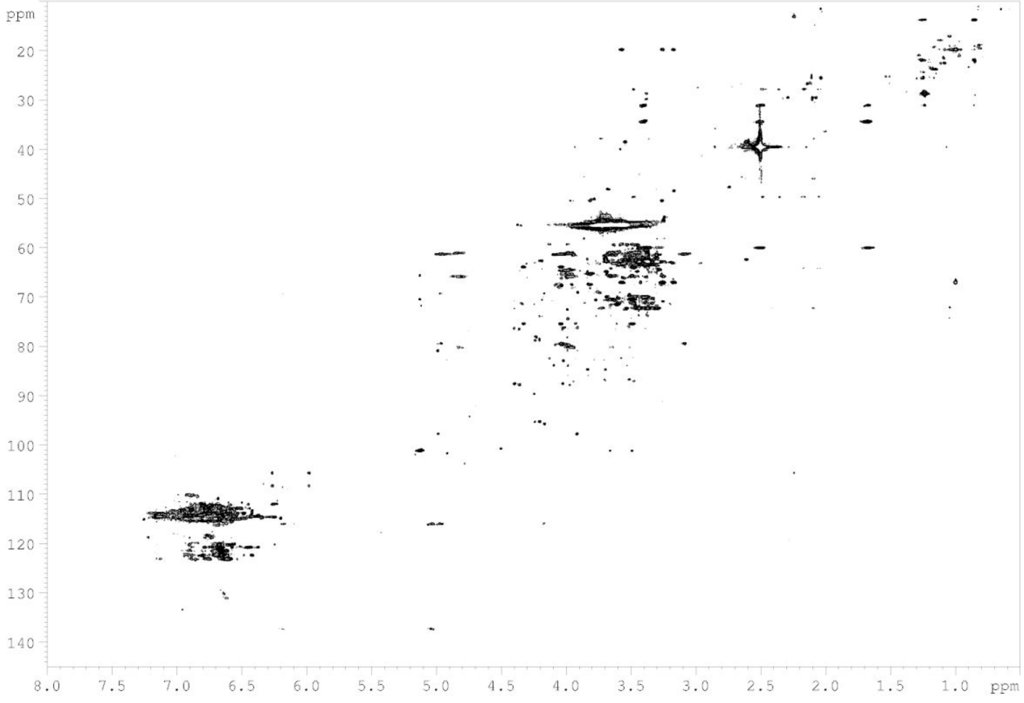

Figure S37. 800.03 MHz 2D ^{1}H-^{13}C HSQC-TOCSY NMR spectrum of starting sodium LS.

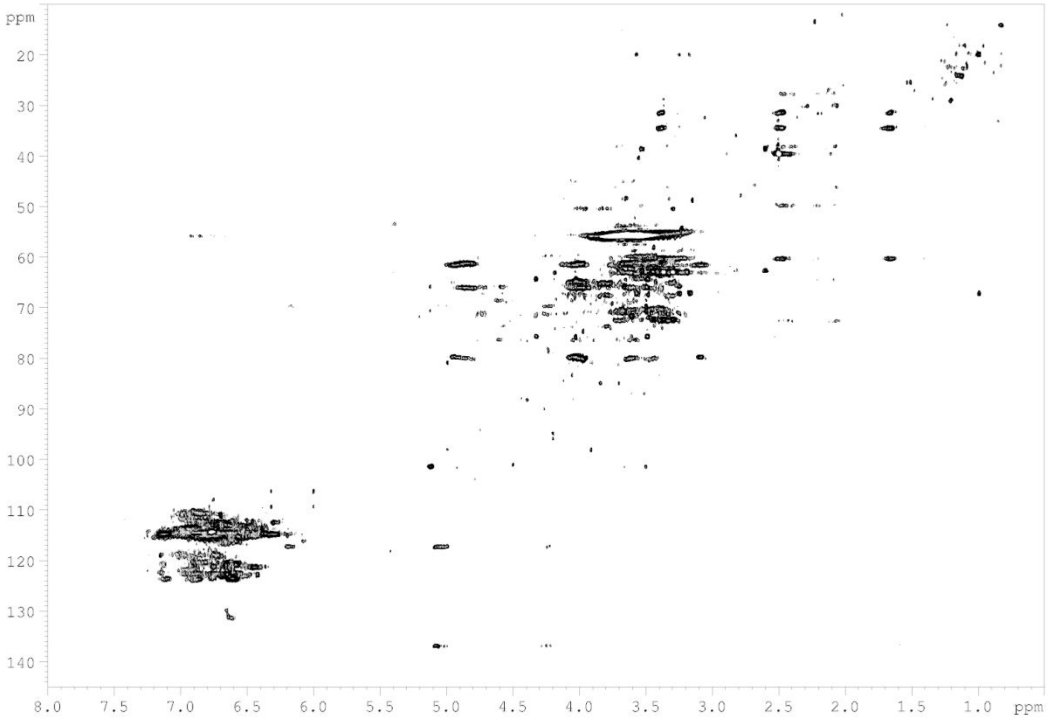

Figure S38. 800.03 MHz 2D ^{1}H-^{13}C HSQC-TOCSY NMR spectrum of fraction F2.

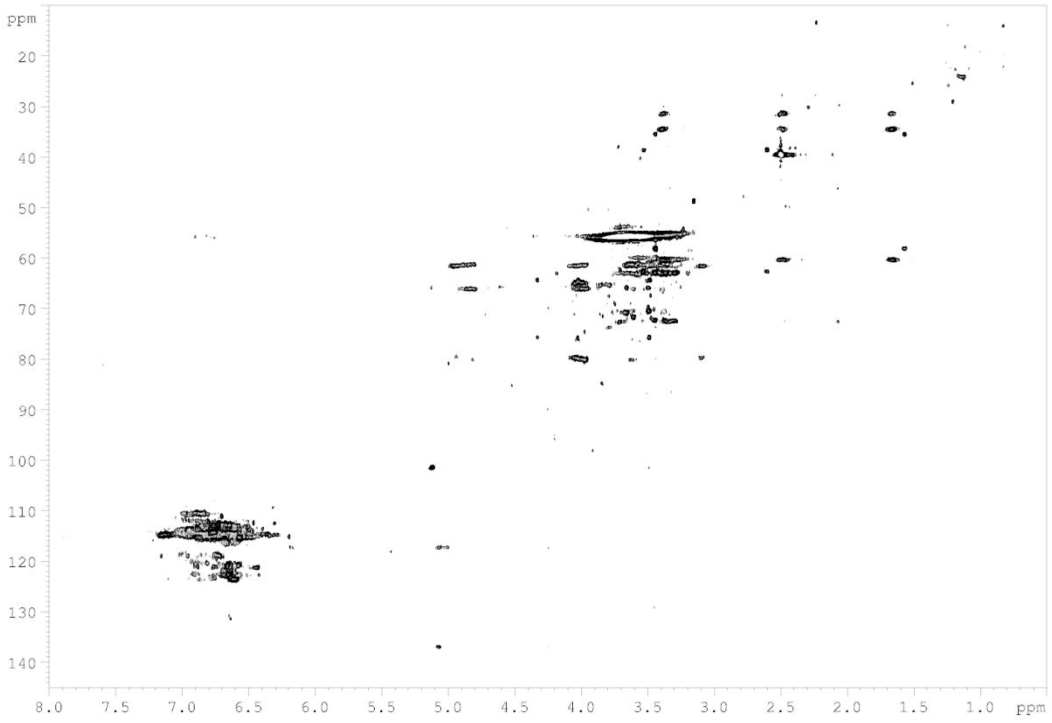

Figure S39. 800.03 MHz 2D ^{1}H-^{13}C HSQC-TOCSY NMR spectrum of fraction F4.

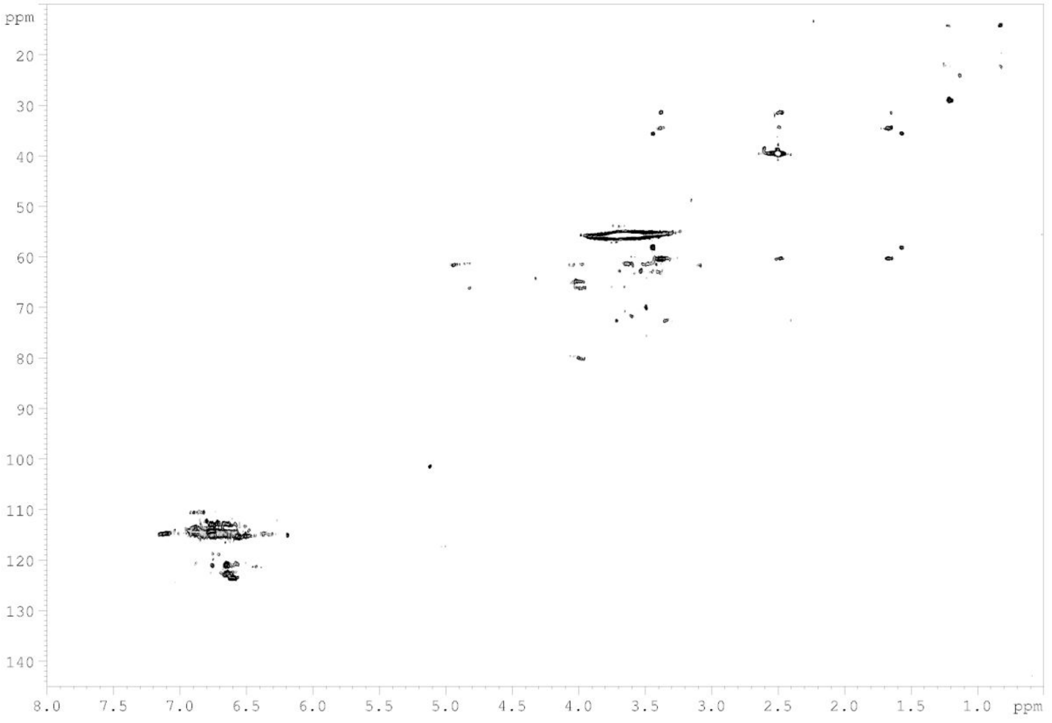

Figure S40. 800.03 MHz 2D ^{1}H-^{13}C HSQC-TOCSY NMR spectrum of fraction F6.

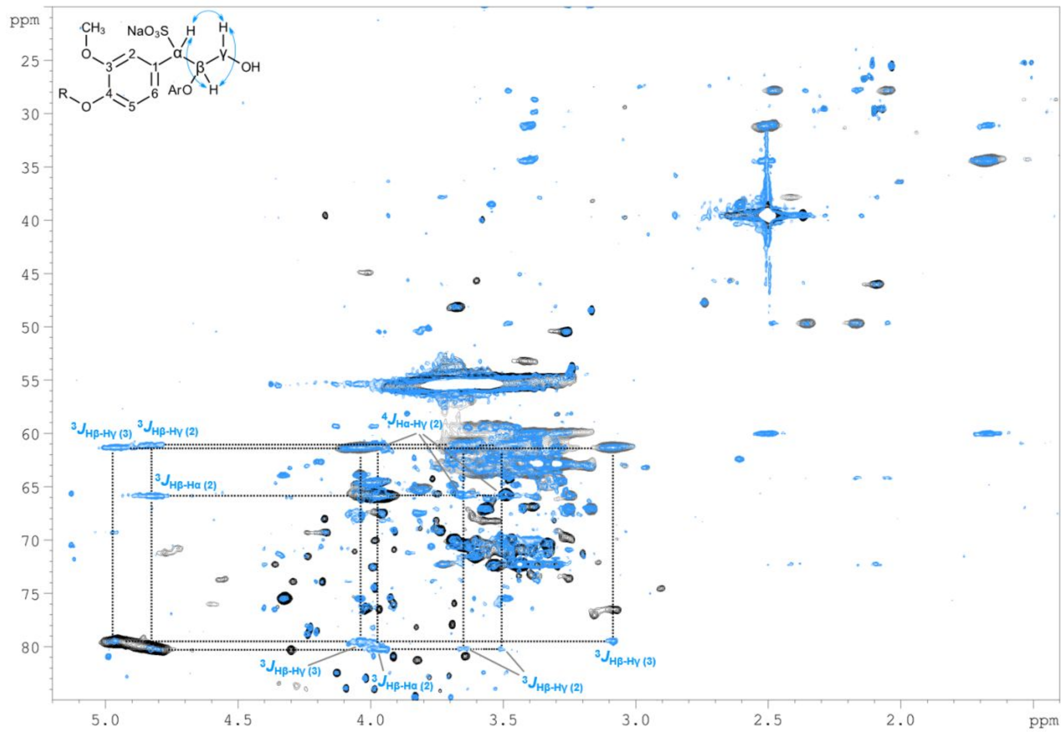

Figure S41. Overlay of 800.03 MHz ^{1}H-^{13}C HSQC-TOCSY (blue) and HSQC (black/grey) NMR spectra of starting LS with correlations for the sulfonated β-O-4 bonding pattern.

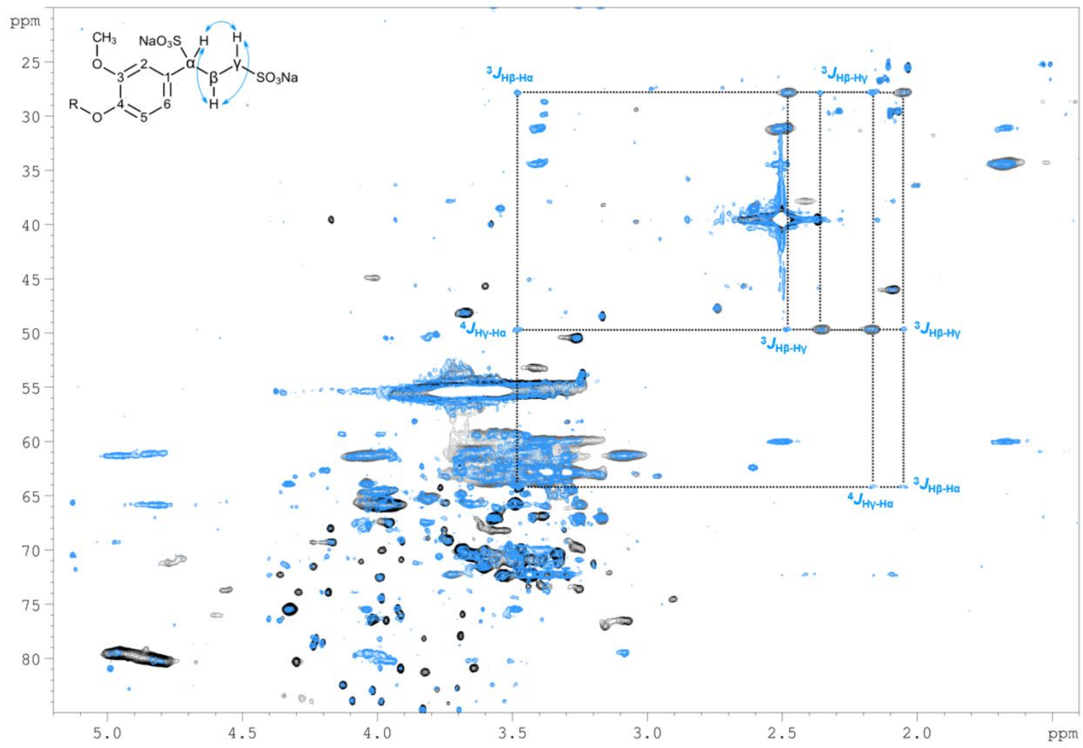

Figure S42. Overlay of 800.03 MHz ^{1}H-^{13}C HSQC-TOCSY (blue) and HSQC (black/grey) NMR spectra of starting LS with correlations for 1,3-disulfonate bonding pattern.

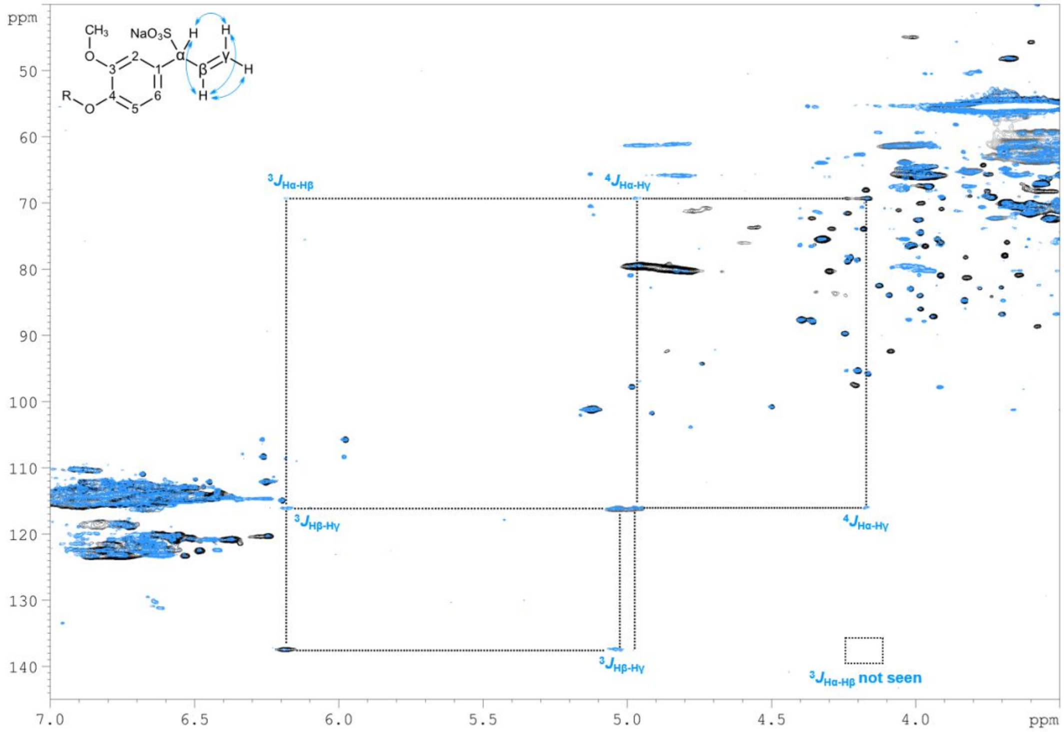

Figure S43. Overlay of 800.03 MHz ^{1}H-^{13}C HSQC-TOCSY (blue) and HSQC (black/grey) NMR spectra of starting LS with correlations for allyl sulfonate bonding pattern.

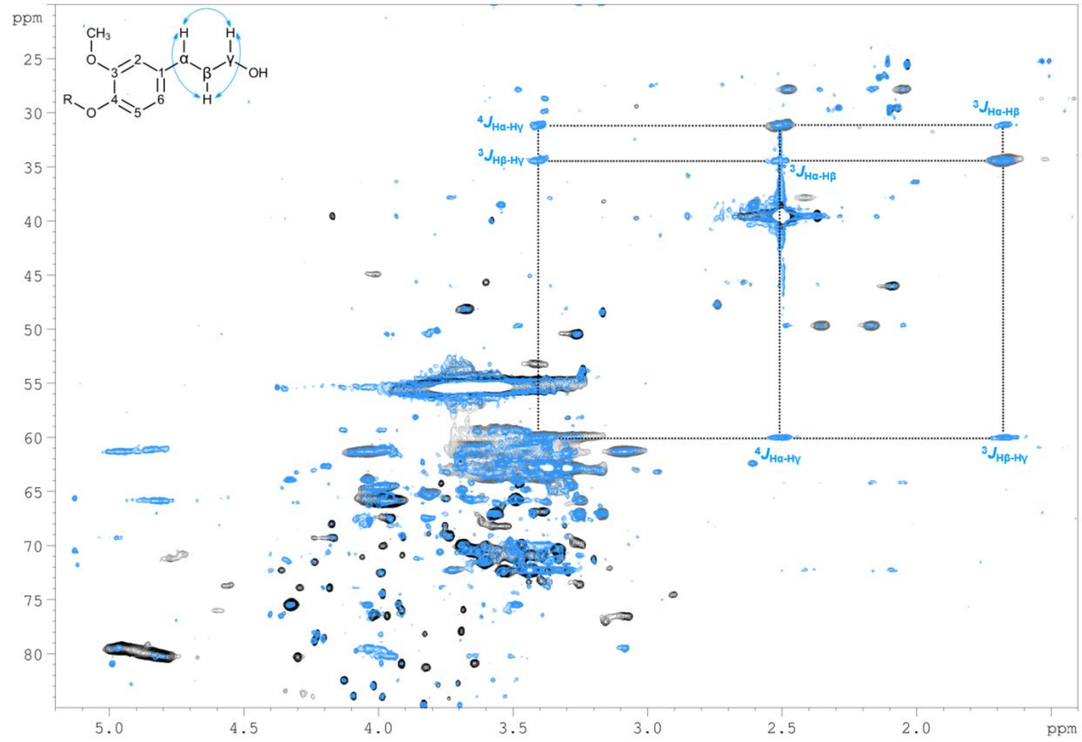

Figure S44. Overlay of 800.03 MHz ^{1}H-^{13}C HSQC-TOCSY (blue) and HSQC (black/grey) NMR spectra of starting LS with correlations for guaiacylpropanol bonding pattern.

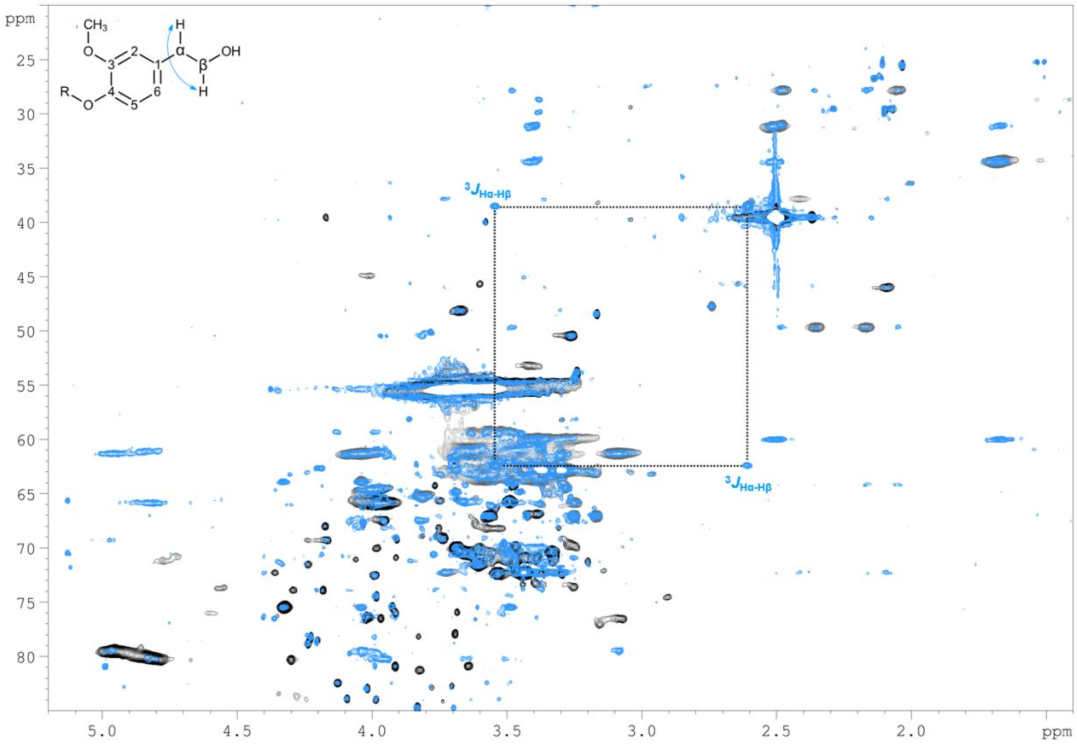

Figure S45. Overlay of 800.03 MHz ^{1}H-^{13}C HSQC-TOCSY (blue) and HSQC (black/grey) NMR spectra of starting LS with correlations for guaiacylethanol bonding pattern.

7. 2D HMBC NMR Spectra

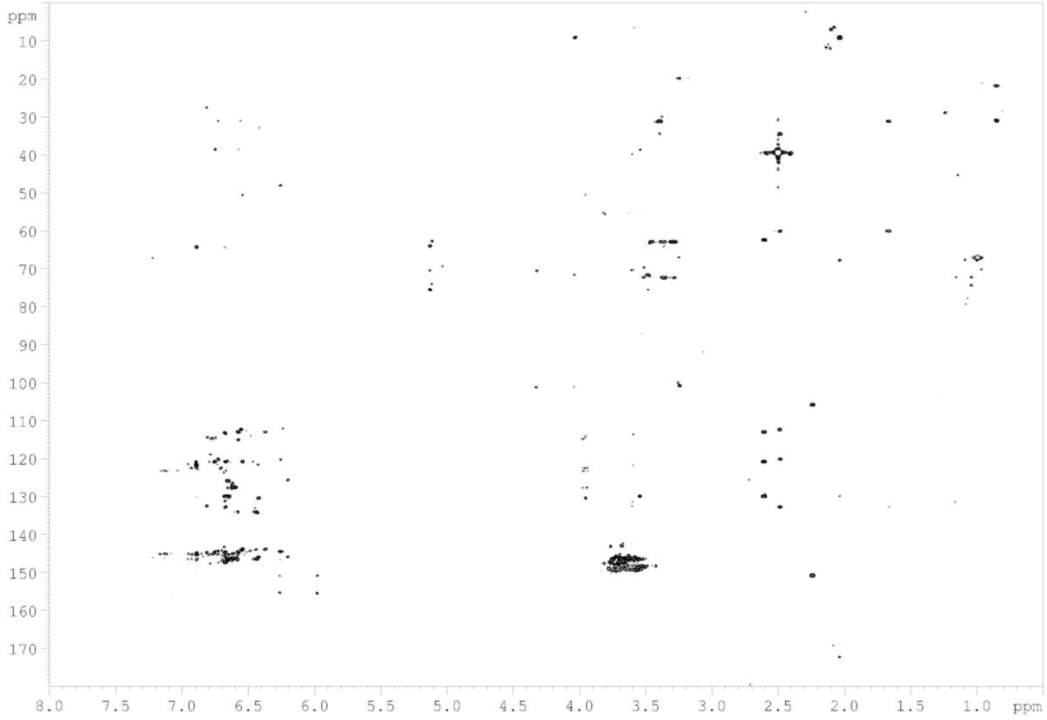

Figure S46. 800.03 MHz ^{1}H-^{13}C HMBC NMR spectrum of starting sodium LS.

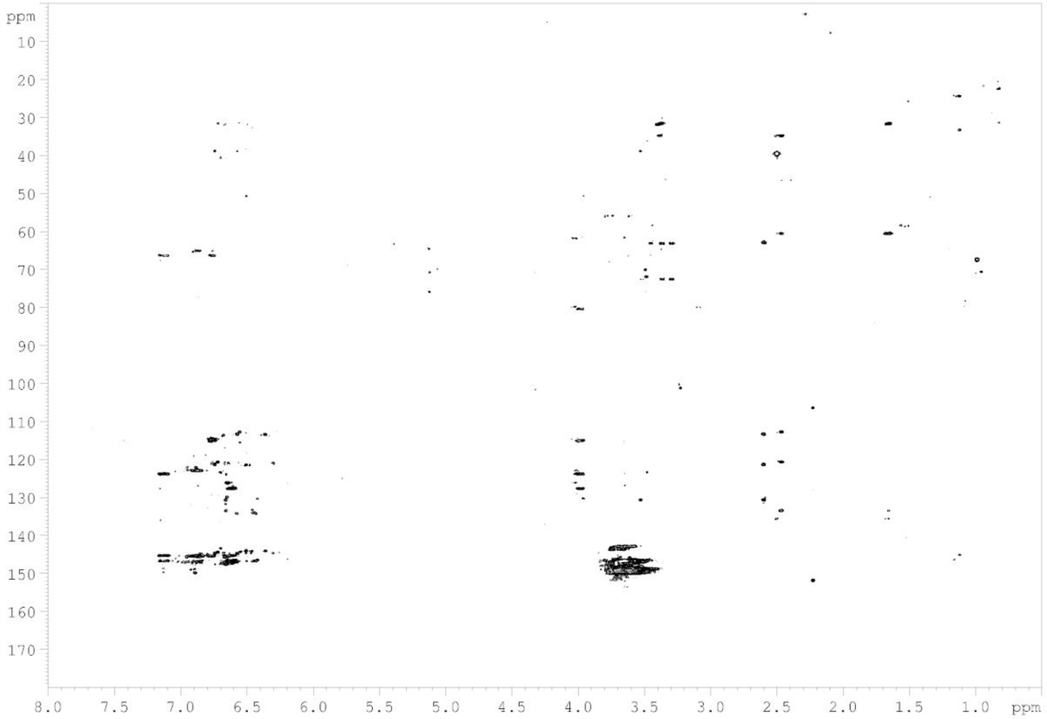

Figure S47. 800.03 MHz ^{1}H-^{13}C HMBC NMR spectrum of fraction F2.

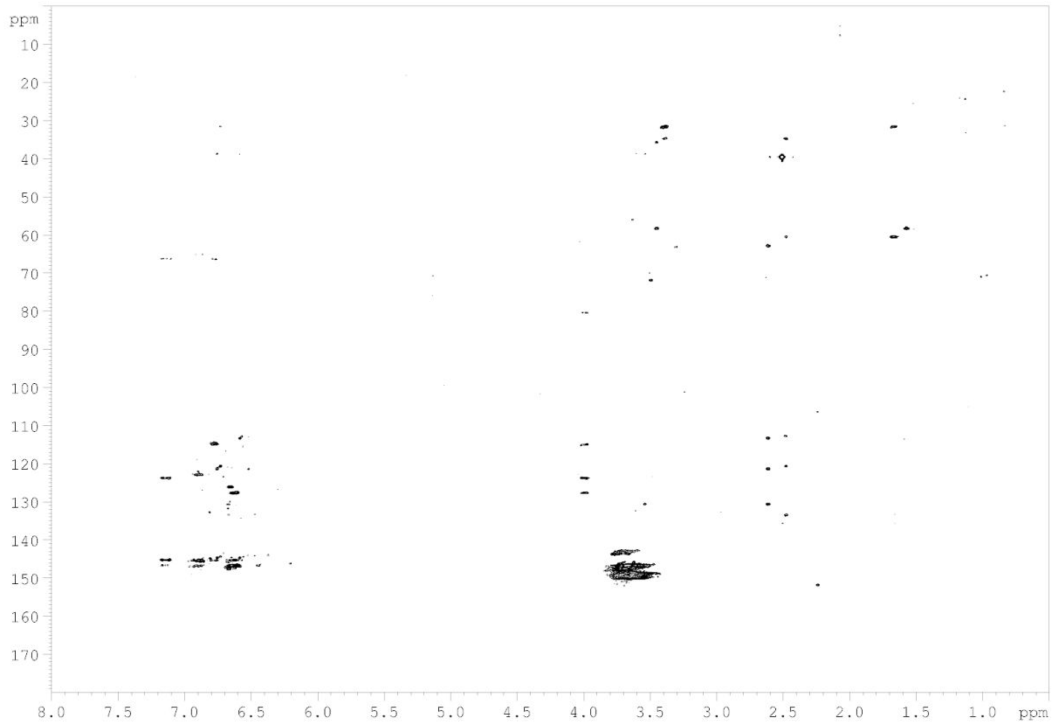

Figure S48. 800.03 MHz ^{1}H-^{13}C HMBC NMR spectrum of fraction F4.

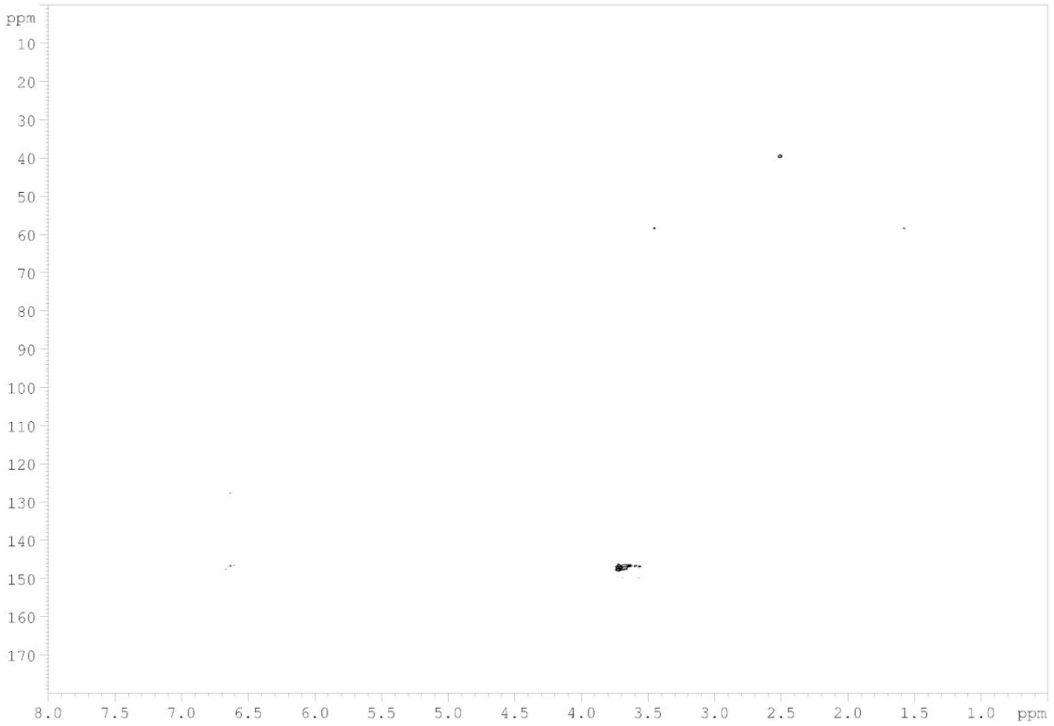

Figure S49. 800.03 MHz ^{1}H-^{13}C HMBC NMR spectrum of fraction F6.

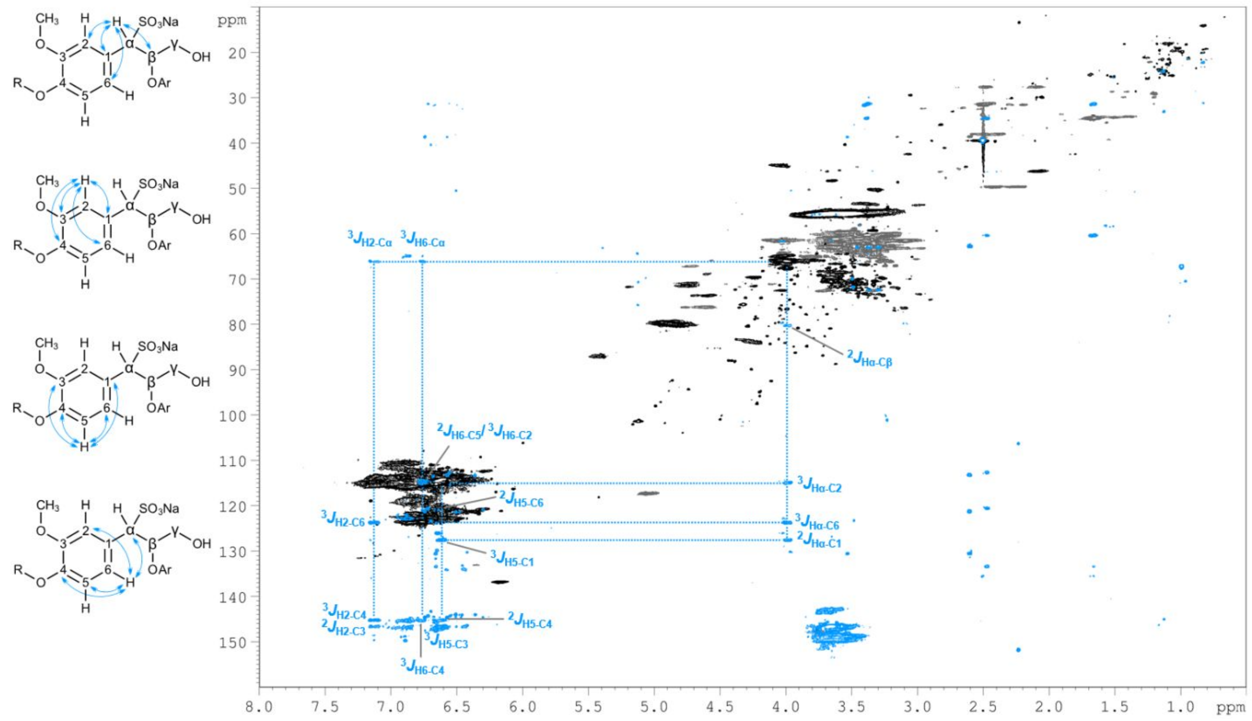

Figure S50. Overlay of 800.03 MHz ^1H-^{13}C HMBC (blue) and HSQC (black/grey) NMR spectra of fraction F2 with correlation annotations for the sulfonated β-O-4 bonding pattern.

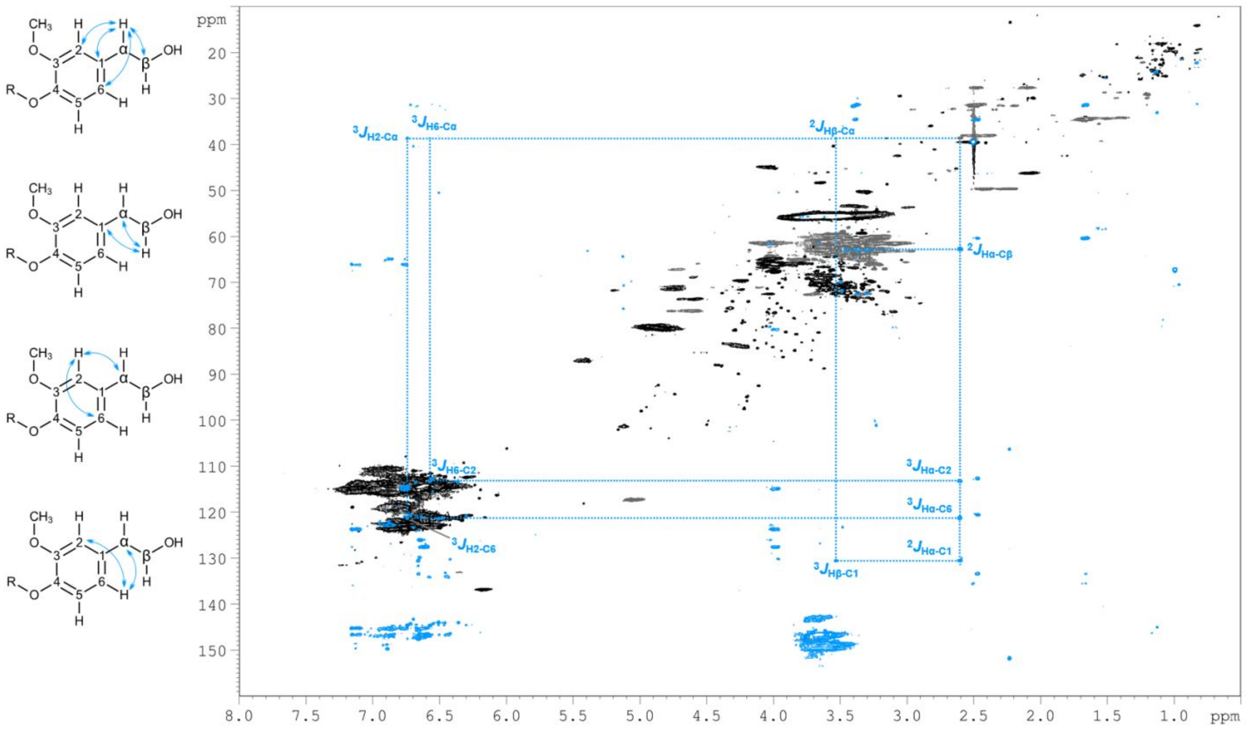

Figure S51. 800.03 MHz ^1H-^{13}C HMBC (blue) and HSQC (black/grey) NMR spectra of fraction F2 with correlation annotations for guaiacylethanol.

8. ^{31}P NMR Spectra

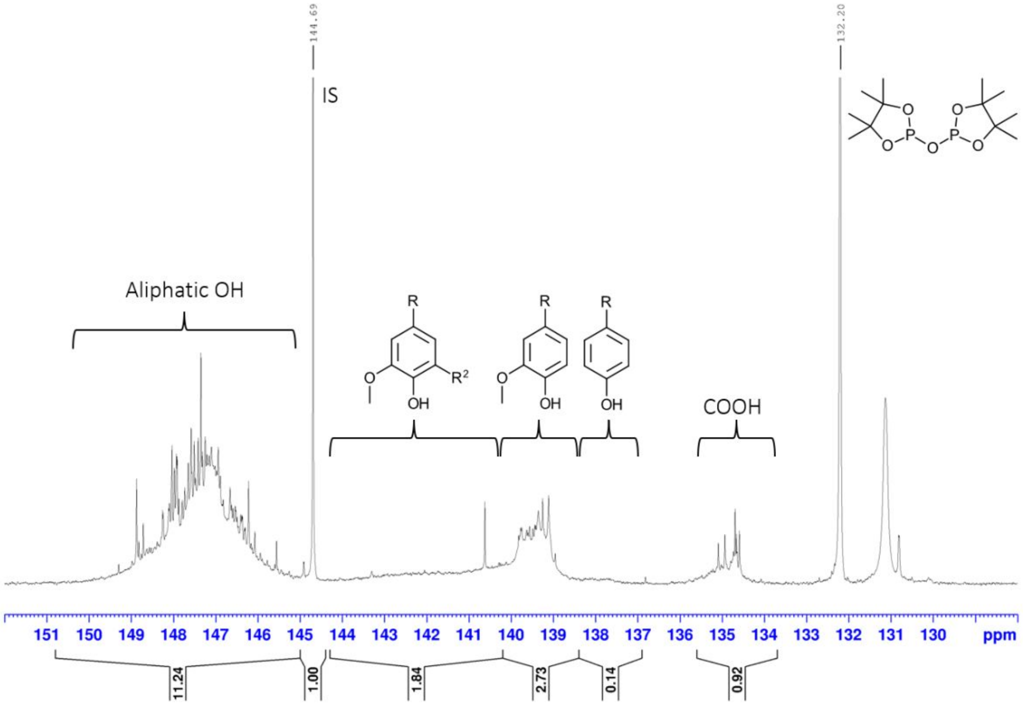

Figure S52. 202.46 MHz ^{31}P NMR spectrum of starting sodium lignosulfonate derivatized with Cl-TMDP.

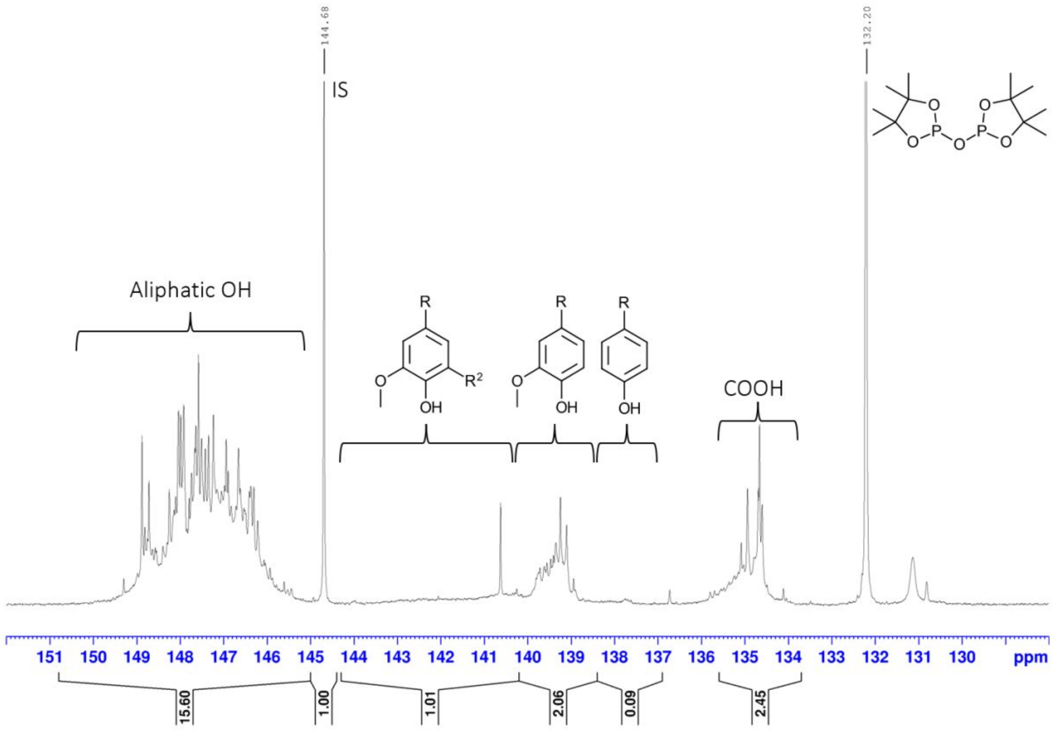

Figure S53. 202.46 MHz ^{31}P NMR spectrum of fraction F1 derivatized with Cl-TMDP.

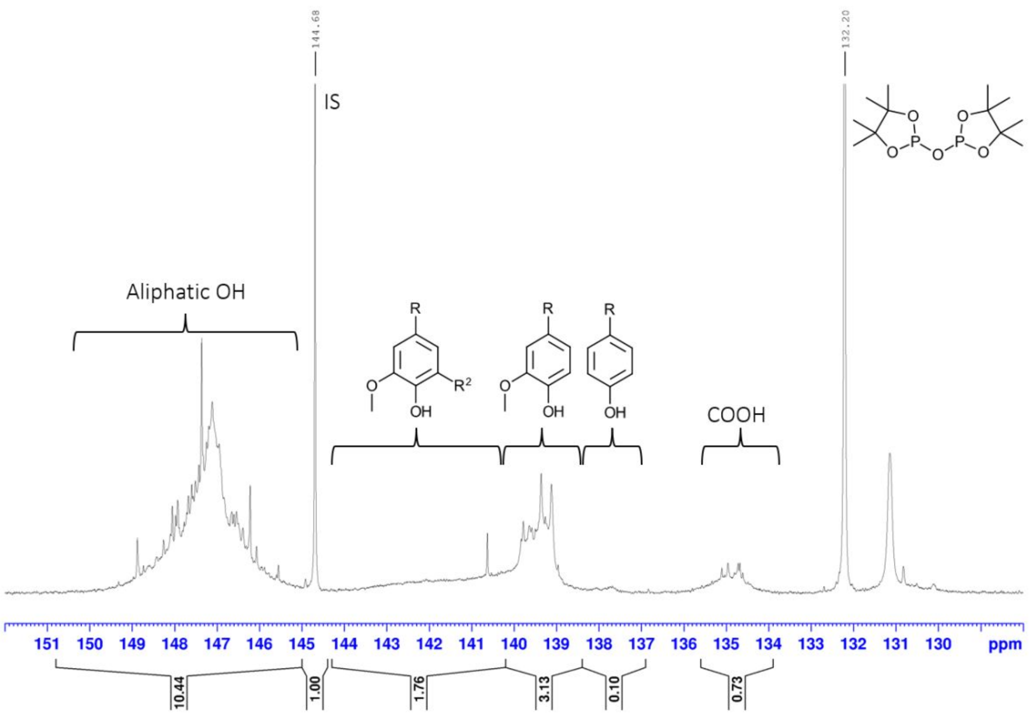

Figure S54. 202.46 MHz ^{31}P NMR spectrum of fraction F2 derivatized with Cl-TMDP.

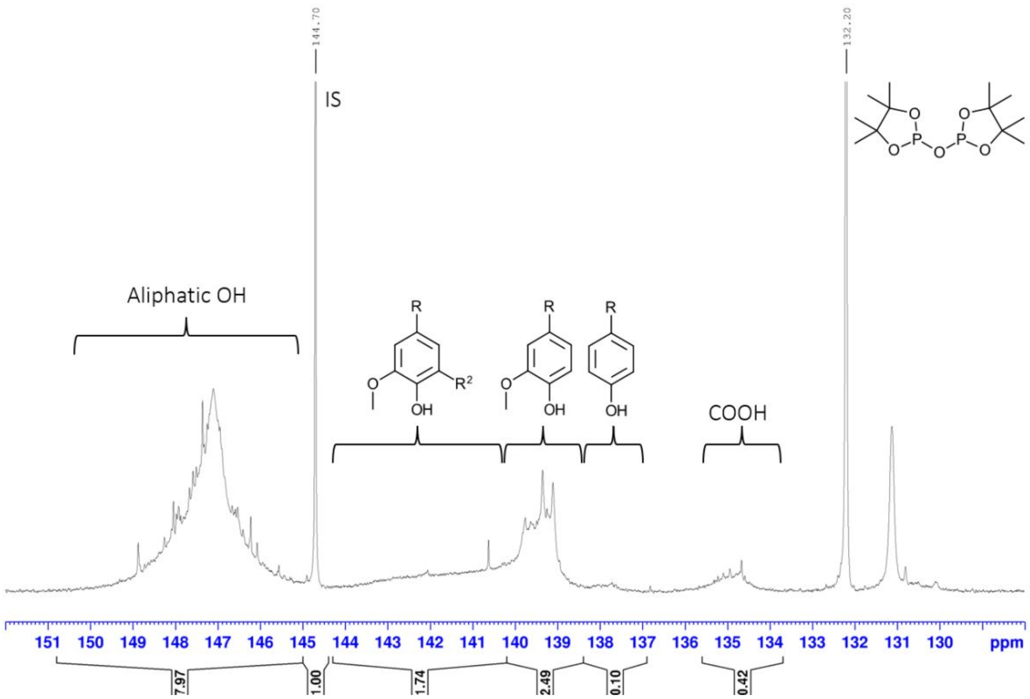

Figure S55. 202.46 MHz ^{31}P NMR spectrum of fraction F3 derivatized with Cl-TMDP.

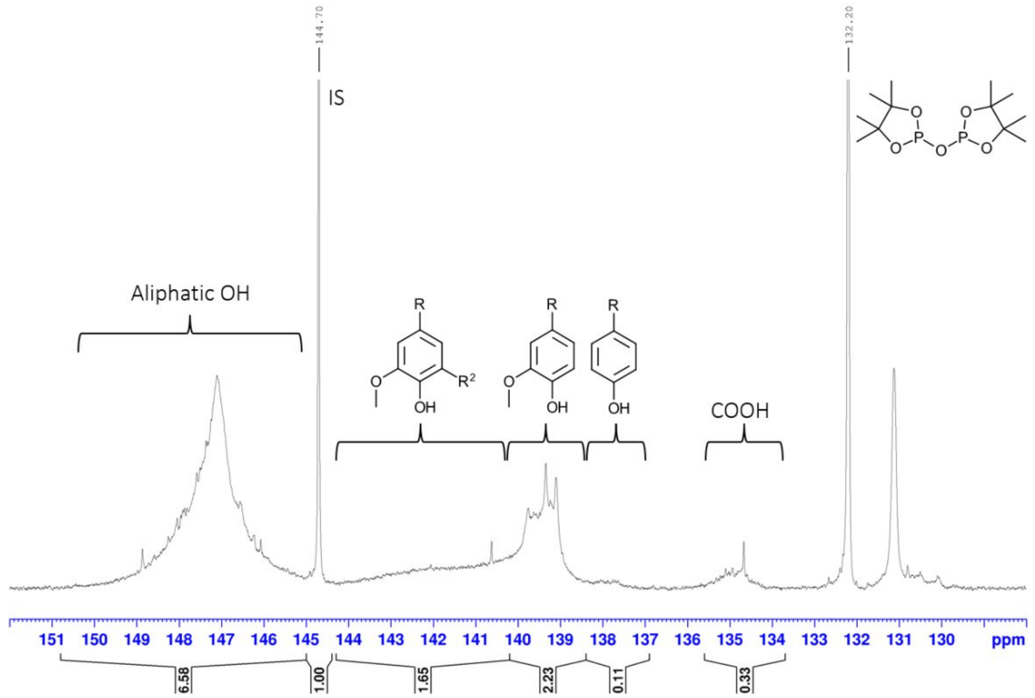

Figure S56. 202.46 MHz ^{31}P NMR spectrum of fraction F4 derivatized with Cl-TMDP.

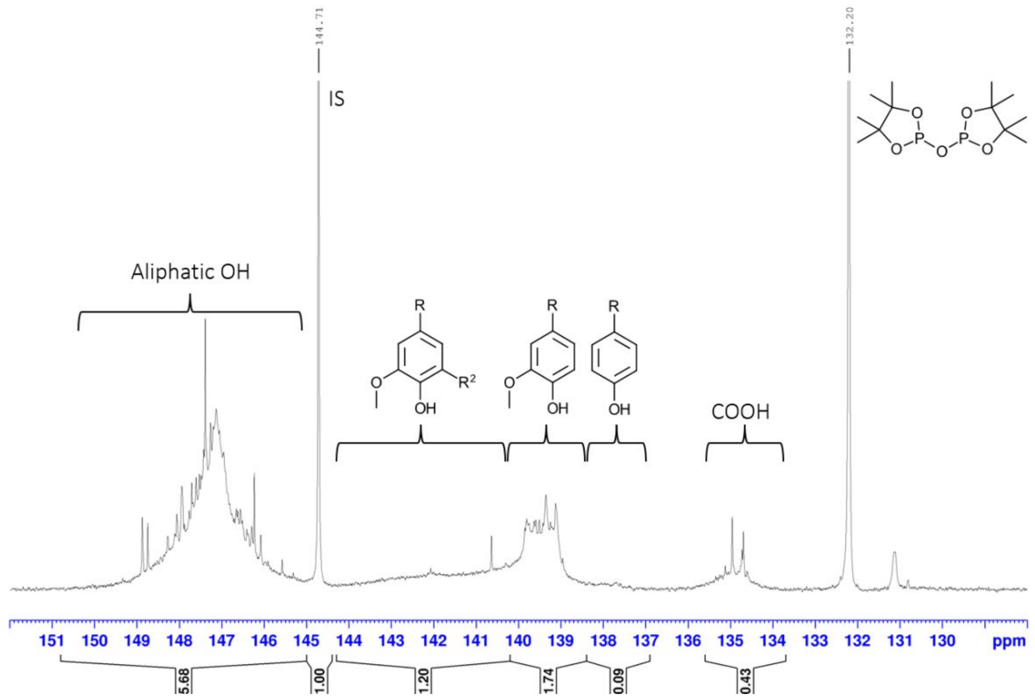

Figure S57. 202.46 MHz ^{31}P NMR spectrum of fraction F5 derivatized with Cl-TMDP.

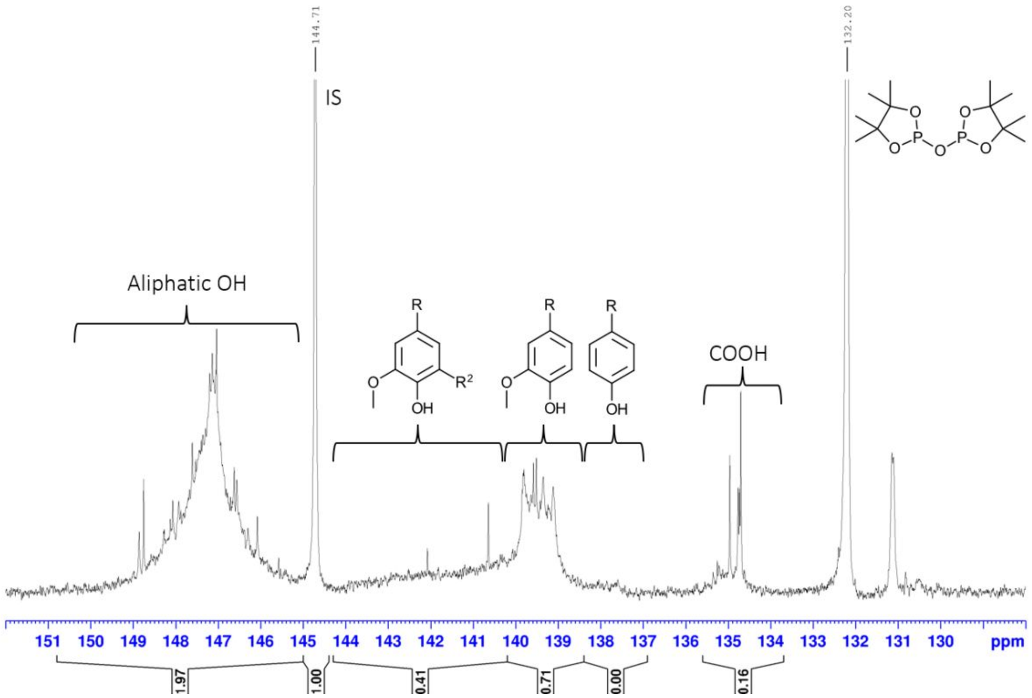

Figure S58. 202.46 MHz ^{31}P NMR spectrum of fraction F6 derivatized with Cl-TMDP.

9. Signal assignment from NMR data

Table S4. Signal assignment of bonding patterns from HSQC NMR spectra.

**Lignin interunit bonds**	**Chemical shift (ppm, δ_H/δ_C)**
A - β-O-4 sulfonate α	4.02/64.9 (syn)<br>4.01/66.0 (anti)
A - β-O-4 sulfonate β	4.94/79.6 (syn)<br>4.84/80.0 (anti)
A - β-O-4 sulfonate γ	4.04/61.5+3.09/61.5 (syn)<br>3.62/61.1+3.45/61.1 (anti)
A - β-O-4 sulfonate C1	127.7
A - β-O-4 sulfonate C2/C5	115.1

A - β-O-4 sulfonate C3	147.1
A - β-O-4 sulfonate C4	145.4
A - β-O-4 sulfonate C5/C2	115.1
A - β-O-4 sulfonate C6	123.9
B - β-5 sulfonate α	3.59/68.5
B - β-5 sulfonate β	4.04/45.0
B - β-5 sulfonate γ	4.60/76.3
C – Allyl sulfonate α	4.24/69.9
C – Allyl sulfonate β	6.17/136.9
C – Allyl sulfonate γ	5.05/117.3
D – 1,3-Disulfonate α	3.52/64.4
D – 1,3-Disulfonate β	2.47/27.7 + 2.06/27.7
D – 1,3-Disulfonate γ	2.37/49.7 + 2.20/49.7
E - β-O-4 α	4.47/71.3
E - β-O-4 β	4.27/83.7
E - β-O-4 γ	Overlapping region
F - β-5 α	5.42/87.0
F - β-5 β	3.40/53.4
F - β-5 γ	Overlapping region
G - β-β α	4.56/85.3
G - β-β β	4.11/71.1+3.74/71.1

G - β-β γ	3.00/53.8
H - Guaiacylpropanol α	2.49/31.5
H - Guaiacylpropanol β	1.66/34.5
H - Guaiacylpropanol γ	3.38/60.3
H – Guaiacylpropanol C1	133.5
H – Guaiacylpropanol C2	112.8
H – Guaiacylpropanol C3	146.9
H – Guaiacylpropanol C4	145.4
H – Guaiacylpropanol C5	115.6
H – Guaiacylpropanol C6	120.8
I - Guaiacylethanol α	2.60/38.6
I - Guaiacylethanol β	3.53/62.7
I – Guaiacylethanol C1	130.6
I – Guaiacylethanol C2	113.4
I – Guaiacylethanol C3	146.9
I – Guaiacylethanol C4	145.4
I – Guaiacylethanol C5	115.6
I – Guaiacylethanol C6	121.4
**Lignin functional groups**	**Chemical shift (ppm, δ_H/δ_C)**
Methoxy	3.67/55.6
**Lignin hydroxyl groups**	**Chemical shift (ppm, δ_P)**
Aliphatic OH	150.8-145.0
o-Disubstituted	144.3-140.3

o-Monosubstituted	140.2-138.4
Total phenolic OH	144.3-136-9
Carboxylic OH	135.6-133.7
**Monomer impurities**	**(ppm, δ_H/δ_C)**
Glycerol CH	3.44/72.3
Glycerol CH_2	3.36/62.9+3.30/62.9

Table S5. Integrals in absolute values from 2D HSQC NMR spectra of the α-sulfonated β-O-4 bonding pattern (**A**) with corresponding intensity ratios for CH_3O:β-signal and α-signal diastereomers.

**Integral identity**	**SM**	**F2**	**F4**	**F6**
Int. CH_3O	19316728832	3160045.94	2507404.12	1434182.31
Int. β-signal	921836544	257496.25	146092.66	76735
**CH_3O:β-signal ratio**	**95:5**	**92:8**	**94:6**	**95:5**
Int. α-signal major diastereomer	3016586240	591687.88	388131.44	104956.92
Int. α-signal major diastereomer	1320036352	273838.09	157275.5	51483.91
**α-signal diastereomeric ratio**	**70:30**	**68:32**	**71:29**	**67:33**

10. Hydrophobic interaction chromatography

Table S6. HIC peak values expressed as %area.

**Fraction**	**Peak 1**	**Peak 2**	**Peak 3**	**Peak 4**	**Peak 5**

SM	43	28	26	3	1
F1	88	7	4	1	0
F2	82	13	4	1	0
F3	66	26	7	2	0
F4	52	26	7	2	0
F5	42	42	14	14	2
F6	17	37	42	4	0

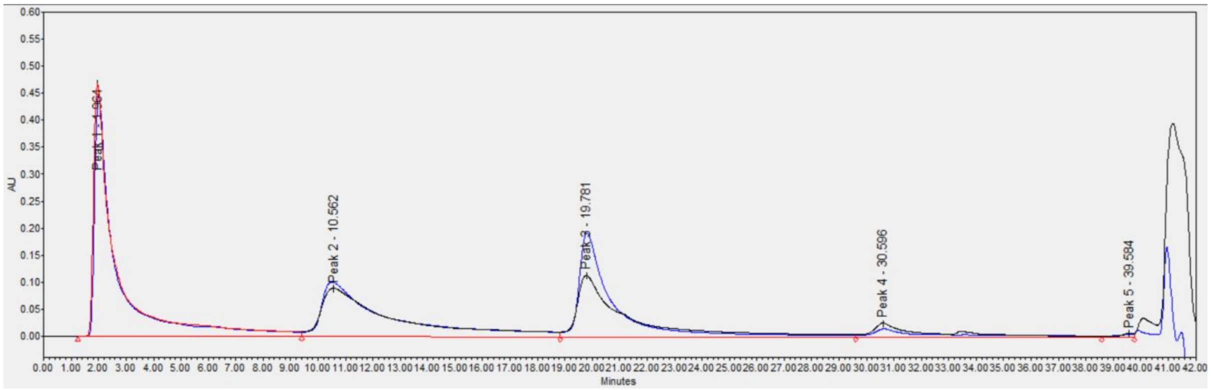

Figure S59. Chromatogram from HIC analysis of SM. Overlay of two parallel runs.

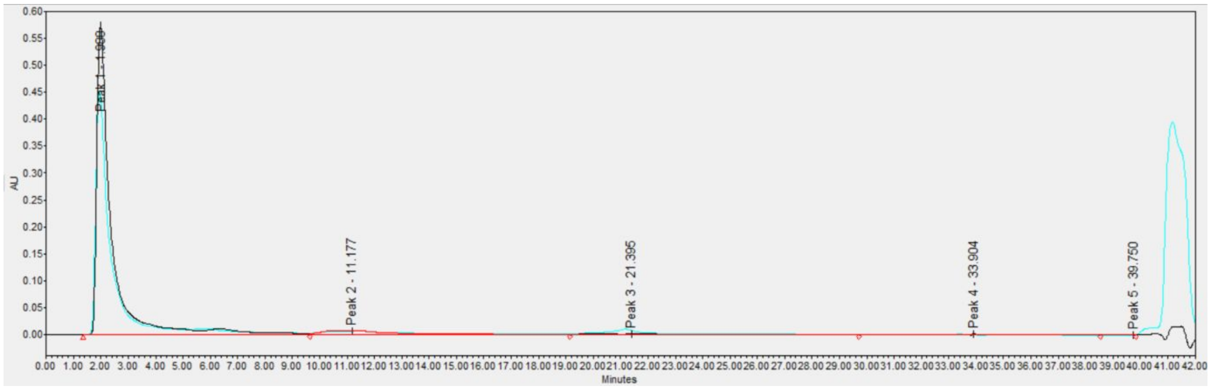

Figure S60. Chromatogram from HIC analysis of F1. Overlay of two parallel runs.

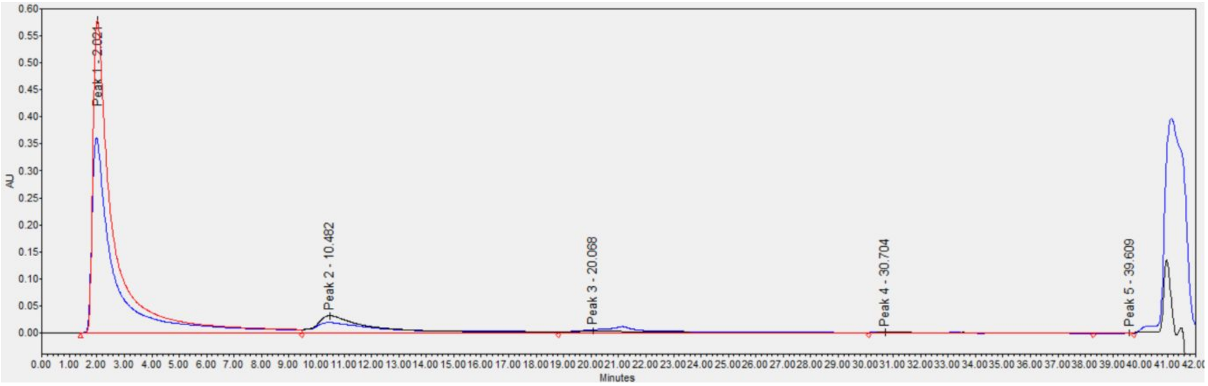

Figure S61. Chromatogram from HIC analysis of F2. Overlay of two parallel runs.

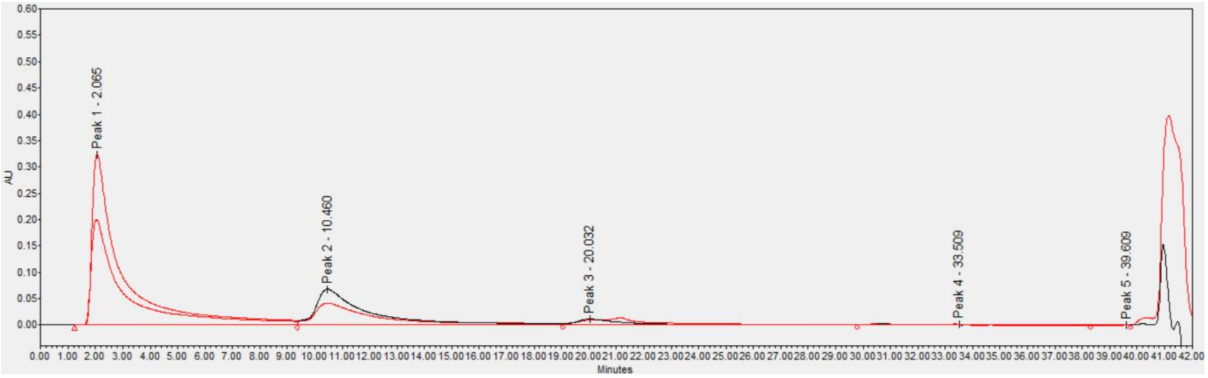

Figure S62. Chromatogram from HIC analysis of F3. Overlay of two parallel runs.

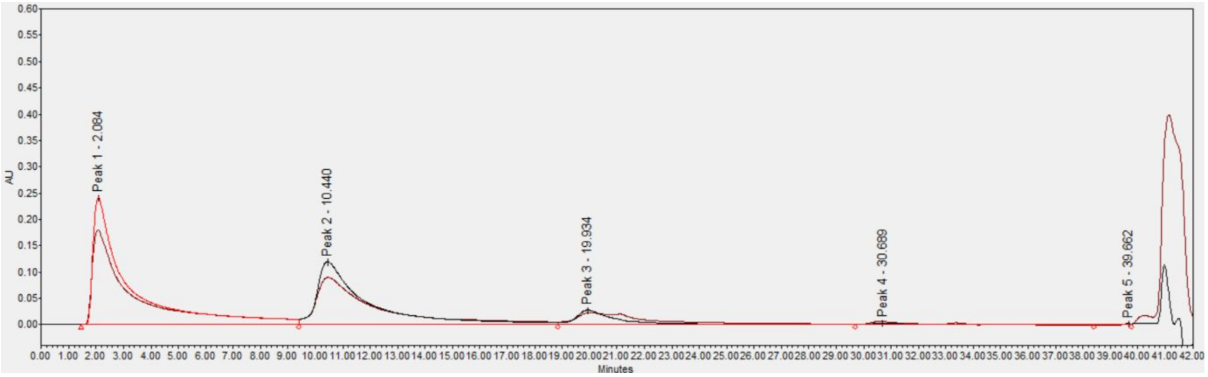

Figure S63. Chromatogram from HIC analysis of F4. Overlay of two parallel runs.

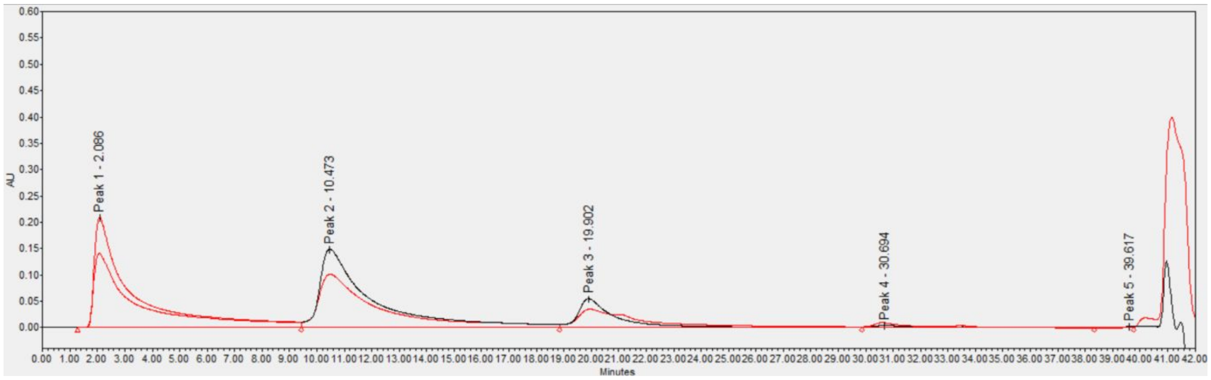

Figure S64. Chromatogram from HIC analysis of F5. Overlay of two parallel runs.

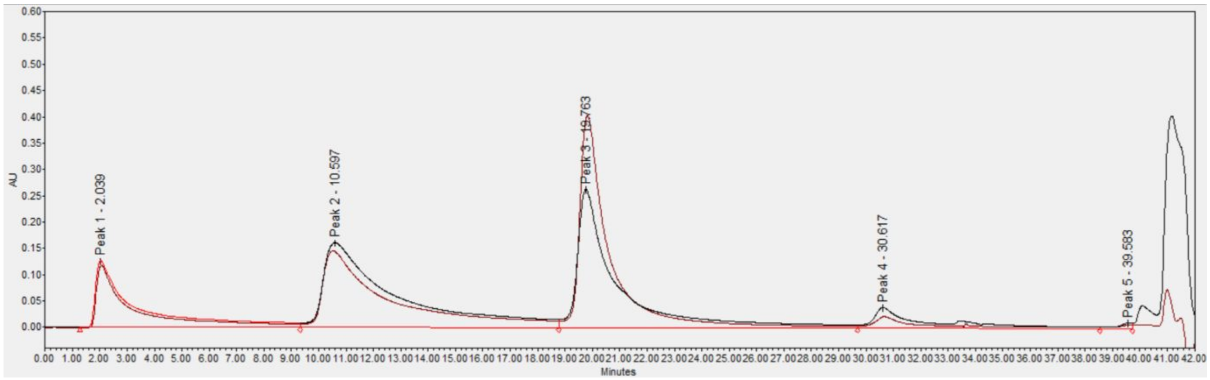

Figure S65. Chromatogram from HIC analysis of F6. Overlay of two parallel runs.
